# High-Yield Methods for Accurate Two-Alternative Visual Psychophysics in Head-Fixed Mice

**DOI:** 10.1016/j.celrep.2017.08.047

**Published:** 2017-09-05

**Authors:** Christopher P. Burgess, Armin Lak, Nicholas A. Steinmetz, Peter Zatka-Haas, Charu Bai Reddy, Elina A.K. Jacobs, Jennifer F. Linden, Joseph J. Paton, Adam Ranson, Sylvia Schröder, Sofia Soares, Miles J. Wells, Lauren E. Wool, Kenneth D. Harris, Matteo Carandini

**Affiliations:** 1 UCL Institute of Ophthalmology, University College London, London WC1E 6BT, UK; 2 UCL Institute of Neurology, University College London, London WC1E 6BT, UK; 3 Centre for Mathematics and Physics in the Life Sciences and Experimental Biology (CoMPLEX), University College London, London, UK; 4 Champalimaud Centre for the Unknown, Lisbon, Portugal; 5 UCL Ear Institute, University College London, London WC1X 8EE, UK

## Abstract

Research in neuroscience increasingly relies on the mouse, a mammalian species that affords unparalleled genetic tractability and brain atlases. Here, we introduce high-yield methods for probing mouse visual decisions. Mice are head-fixed, facilitating repeatable visual stimulation, eye tracking, and brain access. They turn a steering wheel to make two alternative choices, forced or unforced. Learning is rapid thanks to intuitive coupling of stimuli to wheel position. The mouse decisions deliver high-quality psychometric curves for detection and discrimination and conform to the predictions of a simple probabilistic observer model. The task is readily paired with two-photon imaging of cortical activity. Optogenetic inactivation reveals that the task requires mice to use their visual cortex. Mice are motivated to perform the task by fluid reward or optogenetic stimulation of dopamine neurons. This stimulation elicits a larger number of trials and faster learning. These methods provide a platform to accurately probe mouse vision and its neural basis.

## Highlights

• A platform to probe visual discrimination and its neural basis in head-fixed mice
• Extension of classic two-alternative choice design to unforced choices
• Probabilistic observer model for mouse decisions and effects of inactivation
• Optogenetic stimulation of dopaminergic neurons can replace water control

## Graphical Abstract

**Figure:**
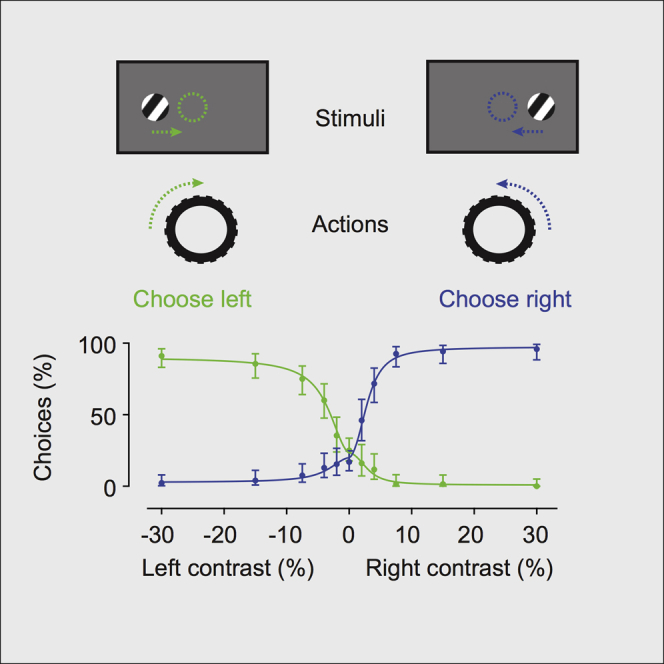

## Teaser

Burgess et al. introduce methods to probe visual discrimination and its neural basis in head-fixed mice. Mice turn a steering wheel to make two alternative choice, and their behavior matches a simple probabilistic observer. The task engages and requires the visual cortex. Optogenetic stimulation of dopaminergic neurons can replace water control.

## Introduction

Mice are increasingly used in research to understand the mammalian brain. The ease of husbandry, breeding, and handling has long been recognized, with the establishment of inbred lines to control for genetic variation (Beck et al., 2000). Today, the mouse offers an unrivaled arsenal of tools to the neuroscientist, from atlases of gene expression and connectivity (Lein et al., 2007, Oh et al., 2014, Zingg et al., 2014) to a plethora of genetic tools and transgenic lines (Harris et al., 2014, Heintz and Gerfen,, Huang and Zeng, 2013, Madisen et al., 2015, Madisen et al., 2012). Its lissencephalic cortex also makes it ideally accessible to imaging studies.

Mice are an excellent species for studying perception and cognition. They quickly learn to perform tasks based on touch (Guo et al., 2014a), olfaction (Liu et al., 2014, Resulaj and Rinberg, 2015), hearing (Hangya et al., 2015, Jaramillo and Zador, 2014, Pinto and Dan, 2015, Sanders and Kepecs, 2012), or vision (Andermann et al., 2010, Busse et al., 2011). Some of these tasks have been extended to probe not only perception but also cognition (Bussey et al., 2012, Nithianantharajah et al., 2015).

Contrary to past preconceptions, mice make major use of vision (Carandini and Churchland, 2013, Huberman and Niell, 2011). Their visual cortex comprises at least 12 retinotopic areas (Garrett et al., 2014, Glickfeld et al., 2014, Wang and Burkhalter, 2007). The division of labor across these areas and other general principles of visual function are likely to be conserved across species (Wang et al., 2011) and may be fruitfully investigated in the mouse.

Studying the neural activity underlying visually driven behavior, however, requires careful psychophysical means that constrain task design (Carandini and Churchland, 2013). An ideal task should (1) allow continuous control of visual stimulation and accurate measurement of eye position; (2) be easily paired with brain recordings or manipulations; (3) require a behavioral response that does not confound the neural activity related to sensory processing and decision-making; (4) be robust to changes in the observer’s tendency to respond; (5) be learned quickly and reliably by most subjects; (6) yield many trials per stimulus and session, to deliver precise psychometric curves relating task performance to visibility; (7) yield close to 100% accuracy on easy trials, to distinguish errors due to the limits of vision from those due to other sources (disengagement, confusion about the task rules, motor errors); and (8) be flexible, so that its design can be made more complex if needed. Finally, it would be ideal if the task could (9) involve only positive reward, without requiring controlled access to food or water.

These fundamental requirements are not met by existing techniques for mouse visual psychophysics.

The first two requirements—control of visual stimulation and the ability to record and manipulate neuron activity—strongly argue in favor of head fixation, ruling out techniques based on swimming (Prusky et al., 2000) or nose poking (Busse et al., 2011, Bussey et al., 2012, Long et al., 2015, Nithianantharajah et al., 2015). Some approaches available to study vision are compatible with head fixation, but they probe innate subcortical behaviors such as the optokinetic reflex (Cahill and Nathans, 2008).

The third requirement—a behavioral response that does not confound sensory activity—rules out behavioral reports such as locomotion or navigation (Harvey et al., 2012, Poort et al., 2015, Wekselblatt et al., 2016). These elicit strong responses in mouse visual cortex (Niell and Stryker, 2010), confounding sensory or decision-related signals.

The fourth requirement—robustness to the observer’s tendency to respond—argues for having the observer choose between concurrent stimuli (Carandini and Churchland, 2013), like in a two-alternative choice design. This rules out go/no-go designs such as those in which the mouse reports the presence of a visual stimulus by licking a single spout (Andermann et al., 2010, Glickfeld et al., 2013, Goard et al., 2016, Lee et al., 2012). Promising methods for two-alternative choices in head-fixed mice are available to probe audition, somatosensation, and olfaction (Guo et al., 2014a, Resulaj and Rinberg, 2015, Sanders and Kepecs, 2012), but not to study vision.

Finally, all existing techniques make use of implicit punishment: the reward redresses an unpleasant circumstance, such as swimming in deep water (Prusky et al., 2000) or having limited access to drinking water (Andermann et al., 2010, Busse et al., 2011, Bussey et al., 2012, Glickfeld et al., 2013, Lee et al., 2012, Long et al., 2015, Nithianantharajah et al., 2015).

We developed a task that meets the above requirements with a behavioral response based on turning a steering wheel left or right to make a two-alternative choice between visual stimuli. The choice of a steering wheel was inspired by tasks that probe hearing and olfaction, which involve a conveyor belt or a spherical ball (Resulaj and Rinberg, 2015, Sanders and Kepecs, 2012). To train the mice in this task, we introduced an intuitive coupling of the steering wheel to the position of the visual stimuli. Mice learn this task within weeks, they perform it proficiently, and their decisions conform to the predictions of a simple probabilistic observer model. The task can be paired with two-photon imaging, activates visual cortex, requires visual cortex, and can be flexibly extended to probe unforced choices, both for stimulus detection and discrimination.

Mice performed the task when rewarded with water or with stimulation of midbrain dopamine neurons. Optogenetic stimulation of these neurons is known to elicit coarse behavioral outcomes (Tsai et al., 2009) or repetitive actions (Kim et al., 2012). Here, we show that it acts as a powerful reward in precise actions driven by perceptual decisions.

## Results

We first introduce a basic version of the task: two-alternative forced-choice (2AFC) detection with a water reward. We then show that this task is compatible with cortical recordings, that it can be extended to unforced choices, that it elicits decisions that conform to a probabilistic model, and that these decisions require visual cortex. Finally, we illustrate a variation in which the reward is optogenetic stimulation of dopamine neurons and one that requires discrimination between two stimuli.

### The Basic Task: Two-Alternative Forced Choice

**Figure 1:**
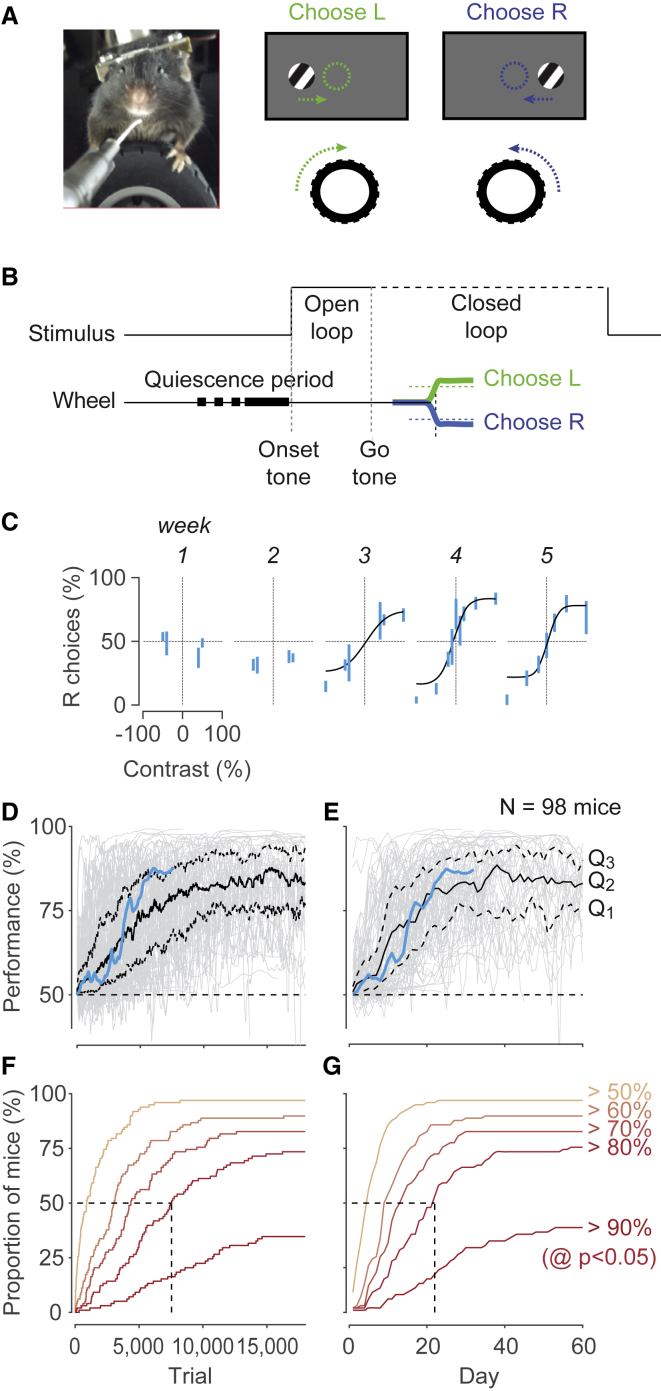
The 2AFC Version of the Stimulus Detection Task (A) Left: a head-fixed mouse with forepaws on a steering wheel used to make choices. Right: at onset, the grating is either on the left or on the right, and the mouse turns the wheel (arrows) to move the grating to the center (dashed circles). (B) Time course of the basic task. Mice start the trial by holding the wheel still (quiescence). An onset tone may be played. The stimulus appears. Its position is initially fixed (open loop). After an optional go tone, stimuli become coupled with wheel position (closed loop). Choices are registered when the stimulus reaches the center of the screen (correct) or an equal distance in the opposite direction (incorrect). (C) Psychometric data obtained in the first 5 weeks for an example mouse. Bars show the percentage of times the mouse chose the right stimulus (95% binomial confidence intervals), as a function of stimulus contrast. By convention, we plot contrast of left stimuli as negative. Curves are fits with a psychometric curve. (D) Learning rates for a population of 98 mice. Performance is assessed on easy stimuli (≥40% contrast), as a function of number of trials. Blue trace highlights the example mouse in (C). Gray traces indicate performance by individual mice. Black traces indicate the 3 quartiles: the median (Q2) and the 25th and 75th percentiles (Q1 and Q3). The approximate chance level is 50% (dashed line). (E) Same as in (D), as a function of training days. (F) Cumulative probability of proportion of mice surpassing a given performance level as a function of trial number. (G) Same as in (F), as a function of training days. See also Figures S1–S3.

The head-fixed mouse is trained to select one of two choices by turning a steering wheel placed under its front paws (Figure 1A). It was highly advantageous to couple wheel movements to the visual stimuli, so that turning the wheel would accordingly move the stimuli (Figure 1A, right; Movie S1). The mouse indicates its choice by bringing one stimulus to the center of the visual field.

The typical sequence of trial events was as follows (Figure 1B). First, the mouse kept the wheel still (quiescent period) to initiate the trial. Second, an onset tone signaled the appearance of the stimuli, and, during an “open loop” period, wheel movements were ignored. Mice generally continued to hold the wheel still in this period (and this could be enforced through training if desired). Third, a go tone was played (e.g., a 12-kHz pure tone lasting 100 ms), after which point the wheel turns resulted in movements of the visual stimuli (“closed loop”; Figure 1B). If the mouse turned the wheel such that the stimulus reached the center of the screen, the animal received water (1–3 μL). If instead the mouse moved the stimulus by the same distance in the opposite direction, this incorrect decision was penalized with a timeout (typically, 2 s) signaled by auditory noise. In either case, the grating remained locked in its response position for 1 s and then disappeared.

Depending on the experimental requirements, in many mice we slightly varied this sequence of events. For instance, if an experiment could tolerate motor actions prior to visual stimulation, we omitted the quiescent period. Similarly, we introduced the open loop period only if we wanted to delay motor actions or visual motion after stimulus presentation. Likewise, we played the onset and go tones only if we did not mind evoking auditory activity, and we shortened the inter-trial interval to maximize trial number. Our analyses here do not distinguish among these variations because other key factors covaried with them: experimenter, time of day, experimental rig, home cage, etc. A proper comparison would have to correct for these factors.

Training for a typical mouse proceeded in two main stages (Figures 1C–1E). We started mice on easy (high) contrasts, until they learned the association between turning the wheel, moving the stimulus, and receiving reward. This association was necessary for learning: in a few attempts in which we did not use the closed loop period, mice did not learn the task. When mice performed above chance for a day or two (which typically occurred by the first week), we introduced lower contrasts. A typical mouse (Figure 1C) reached 56% performance (with 95% confidence) on high contrast stimuli on day 5, after ∼2,300 trials (Figures 1D and 1E, blue), after which we introduced lower contrast stimuli. Psychometric functions of stimulus contrast and position were obtained by week 3 (Figure 1C). By week 4, this mouse had mastered the task.

These results were typical of our population (n = 98 mice; Figures 1D–1G). Most mice were above chance before ∼1,000 trials (Figure 1D), corresponding to a few days of training (Figure 1E). Mice then typically approached steady performance after 7,000–10,000 trials (Figure 1D), i.e., in 20–30 days (Figure 1E). Very few mice (6/98) failed to learn the rudiments of the task (performance significantly above 50%) by trial 5,000 or after 2 weeks (Figures 1F and 1G). Most animals surpassed 80% performance, but a sizeable fraction (38/98) also reached 90% performance (Figures 1F and 1G). This method worked even though different cohorts were trained by different experimenters using different subjective criteria for advancing a mouse from one stage of training to the next.

Once they mastered the task, mice typically produced stereotyped movements, with initial wheel deflections usually matching the final responses (Figure S1). The movements elicited by high contrast stimuli typically had shorter latency and higher peak velocity. Movements otherwise showed little variability across trials. If desired, we could then modify the task by removing the coupling between wheel position and stimulus position, so that the stimulus would stay fixed in its position (Figure S2), or disappear as soon as the movement started.

Some mice moved their eyes following stimulus onset or showed changes in pupil diameter associated with trial structure (Figure S3). These eye movements and pupil dilations, however, varied across trials and across mice, highlighting the importance of imaging the eye in all experiments.

### Simultaneous Recordings in the Visual Cortex

**Figure 2:**
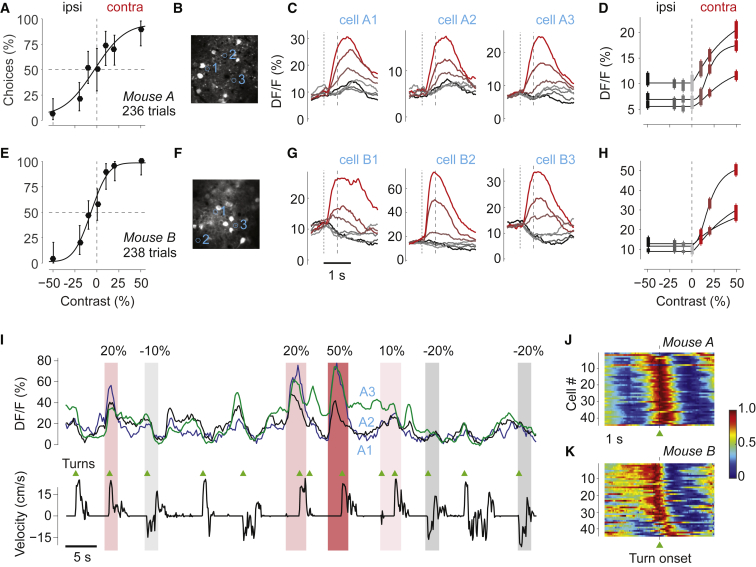
Imaging in V1 during the Task (A) Psychometric curve for an example mouse, measured during two-photon imaging in area V1. Error bars are 95% binomial confidence intervals. (B) Imaging field of view, with 3 cells circled and numbered. (C) Mean calcium activity averaged around the onset of the grating stimulus, grouped by stimulus condition (see color codes in next panel) for the 3 cells. Dotted line marks stimulus onset (preceded by a 2- to 3-s quiescence period). Dashed line marks the beginning of the closed-loop period, when the stimulus becomes movable. Data were taken from 181 trials (22–30 per condition). (D) Response amplitudes of each cell as a function of stimulus contrast. Positive and negative contrast denotes stimuli in the contralateral and ipsilateral visual fields. Amplitude is mean response at 1 s after grating onset. Curves indicates fits of the function p+qf(c), with f(c) defined in Equation 1. Error bars indicate SEM. (E–H) Same as (A)–(D), for a different mouse. Data were taken from 210 trials (24–43 per condition). (I) Example traces from the cells in (B)–(D) in the presence of stimuli of different contrasts (shaded areas) and in relation to wheel velocity (bottom trace). There are strong responses to the visual stimuli but also small responses synchronized with turn onsets (triangles). Onsets and offsets of wheel turns were identified by applying a dynamic threshold based on a Schmitt trigger to the wheel velocity traces. (J) Time course of movement-related activity in the absence of visual stimuli in 45 neurons from each of the 2 mice. Neurons were selected based on the quality of segmentation. We triggered calcium activity on wheel turn onsets, averaged across events, and normalized the results for each neuron (rows) to range from 0 to 1. Neurons were sorted by the amplitude 1 s before turn offset. (K) Same as in (J) for mouse B.

To pair this task with measurements of brain activity, we performed two-photon imaging in primary visual cortex (V1) (Figure 2). We expressed GCaMP6m in V1 neurons via virus injection. In this task version, mice had to hold the wheel still for a 2- to 3-s quiescence period, and the open-loop period lasted 1 s (Figure 1B). During this period, we could image neural responses without the stimulus moving. We chose a field of view with neurons whose receptive fields overlapped with the contralateral stimulus (Figure 2B).

As expected, most visually responsive neurons showed robust responses to contralateral stimuli and no responses to ipsilateral stimuli (Figures 2C and 2D). The amplitudes of these responses grew with the contrast c of contralateral gratings (Figure 2D). We fit these responses with the commonly used function

(1) $$f\left(c\right)=\frac{c^{n}}{c_{50}^{n}+c^{n}},$$

where $c_{50}$ and n are free parameters (Albrecht and Hamilton, 1982, Sclar et al., 1990). These results were robust across mice (e.g., Figures 2E and 2H) and demonstrate that the task can be readily paired with recording techniques requiring high stability and evoke contrast-dependent activity in cortex.

V1 activity also included small fluctuations that tended to precede wheel movements (Figures 2I–2K). Large responses to contralateral stimuli (Figure 2I) were not the sole activity observed. Even in the absence of visual stimuli, activity built up before wheel turns, perhaps reflecting increased alertness (Burgess, 2016), and decayed following the onset of wheel turns (Figures 2J and 2K). This buildup of activity, nonetheless, was dwarfed by visual responses. For instance, for the 6 example cells (Figures 2B–2D and 2F–2H), the build-up activity was 7.5 ± 0.8 times smaller (mean ± SEM) than the responses to 50% contrast contralateral stimuli.

### Two-Alternative Unforced Choice

Next, we extended the two-alternative tasks by adding a “no-go” response option when there was no stimulus. The result is the two-alternative unforced-choice (2AUC) task, which allows one to measure sensitivity and bias separately for the two stimulus locations. This is particularly useful following unilateral manipulations in task context or brain activity (Sridharan et al., 2014).

**Figure 3:**
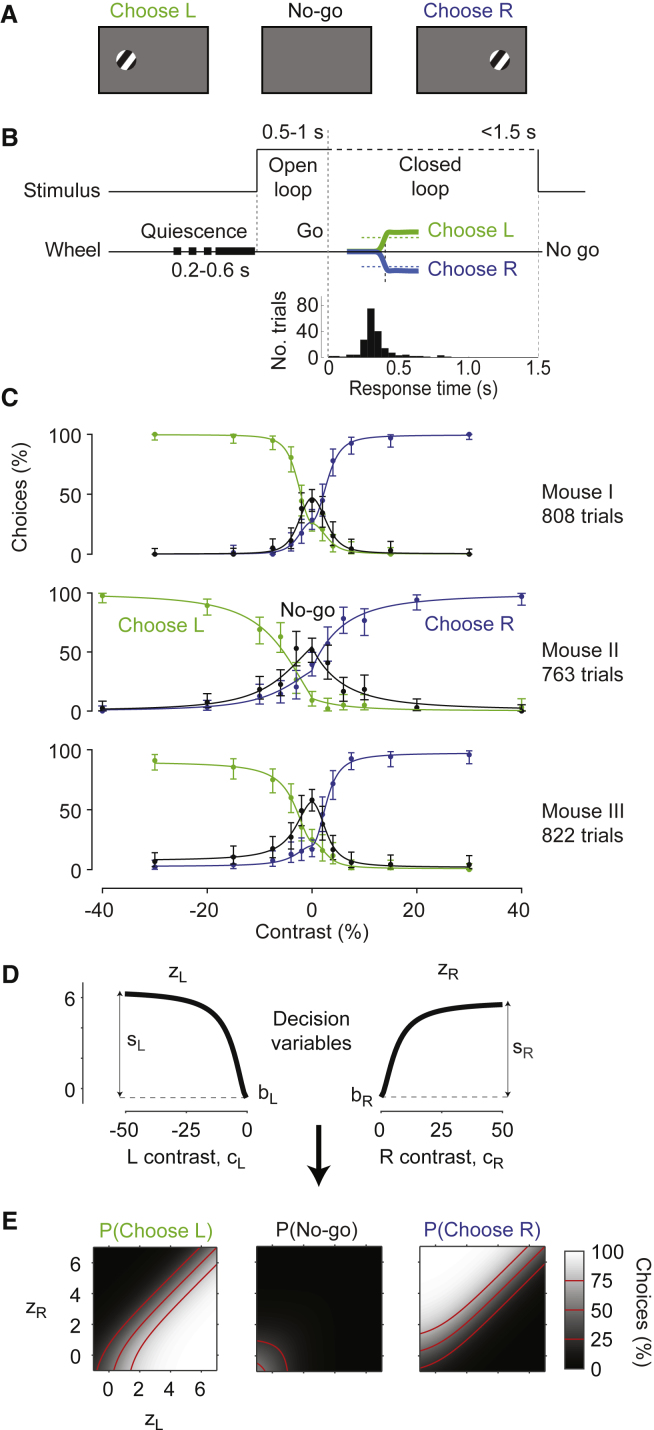
Elaboration of the Stimulus Detection Task in a 2AUC Version (A) In the 2AUC task, the mouse learns to choose left when the stimulus is on the left, choose right when the stimulus is on the right, and hold still (no-go) if the stimulus is absent. (B) Time course of the 2AUC task. At the go cue, the mouse has 1.5 s to move the wheel. Holding the wheel still for this period counts as a no-go choice. Histogram shows a typical distribution of response times in a session (time from go tone to response). (C) Choices as a function of stimulus contrast and position, for three sessions in 3 mice (rows). For each mouse, the data show the proportion of left (green), right (blue), and no-go choices (black) as a function of stimulus contrast. Negative contrast denotes stimuli appearing on the left side. Curves show fits of the probabilistic observer model. Error bars are 95% binomial confidence intervals. (D) The decision variables in the probabilistic observer model, with parameters obtained from mouse 1. The decision variables z_L_ and z_R_ grow with contrast presented on the left or on the right. Each function is defined by 2 parameters: bias, *b*, and sensitivity, *s* (Equations 1 and 2). (E) The probability of left, no-go, and right choices depends on the 2 decision variables. This dependence is parameter-free (Equation 3). See also Figure S4.

Mice were readily able to learn the 2AUC version of the task (Figures 3A–3C). Training started on the two-alternative forced-choice task, then we constrained the response window to 1.5 s and added the no-go condition: when the stimulus was absent (zero contrast), mice earned the reward by not turning the wheel (no-go; Figure 3A) for 1.5 s (Figure 3B). Mice typically learned this new response contingency in 5 or 6 sessions. Their reaction times for left (L) or right (R) responses were much faster than the 1.5-s response window (Figures 3B and S4), indicating that issuing a no-go response was distinct from simply being slow to respond. Mice correctly made most no-go choices at zero contrast, and made progressively fewer of them as stimulus contrast increased (Figure 3C).

This 2AUC version of the task thus yields 3 psychometric curves indicating probability of choosing L, of choosing R, and of choosing no-go (Figure 3C). Although this representation is redundant (the probabilities must sum to 1, so one curve is fully constrained by the other two), it helps to view all 3 to understand the data and to develop a simple observer model to interpret and fit the data.

### Probabilistic Observer Model

The decisions made by the mice closely matched the predictions of a simple probabilistic model. We present here the model for the 2AUC version of the task, which can be easily reduced to the 2AFC version.

In the model, choices depend on two decision variables, one for choosing L and one for choosing R, each depending on the contrast $c_{L}$ and $c_{R}$ on the left and right:

(2) $$\begin{array}{l} z_{L}=b_{L}+s_{L}f\left(c_{L}\right)\\ z_{R}=b_{R}+s_{R}f\left(c_{R}\right) \end{array}.$$

Here, f(c) is the function in Equation 1, $b_{L}$ and $b_{R}$ represent bias toward choosing L or R relative to no-go, and $s_{L}$ and $s_{R}$ measure the weight assigned to visual evidence on the left or right (Figure 3D).

The decision variables, in turn, determine the probabilities $p_{L}$, $p_{R}$, and $p_{0}$ of choosing L, R, or no-go (Figure 3E), and specifically the log odds of choosing L or R versus choosing no-go:

(3) $$\begin{array}{l} \log\left(p_{L}/p_{0}\right)=z_{L}\\ \log\left(p_{R}/p_{0}\right)=z_{R} \end{array}.$$

With 6 free parameters, the model provided good fits to the 22 response probabilities, explaining over 75% of individual choices (curves in Figure 3C). Cross-validation indicated that for these 3 datasets there would be no loss in fit quality if one imposed $s_{L}=s_{R}$, thus removing one free parameter. However, as we will see, these two parameters must be allowed to differ when evaluating the effects of unilateral inactivation.

### Inactivation in the Visual Cortex

**Figure 4:**
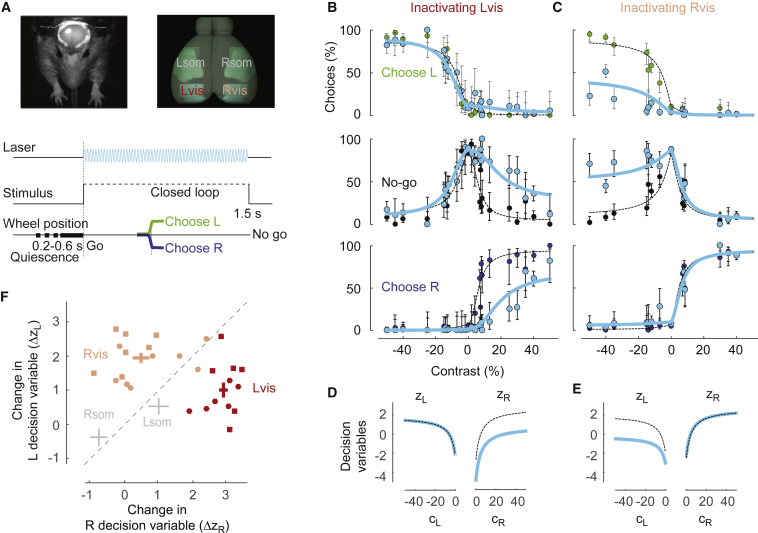
Effects of Optogenetic Inactivation of Visual Cortex (A) Methods of optogenetic inactivation during the 2AUC task. Top left: image of a mouse with the clear skull preparation, with laser spot on right hemisphere. Top right: illustration of the regions inactivated: left and right visual cortex (Lvis and Rvis) and, as a control, left and right somatosensory cortex (Lsom and Rsom). Inactivation of these regions was performed in different sessions. Bottom: time course of the task. In ∼33% of trials, stimuli were accompanied by laser illumination. (B) Effects of inactivation of left visual cortex. Proportion of left, no-go, and right choices as a function of stimulus contrast, under control conditions (green, black, and blue dots) and during optogenetic inactivation (cyan dots). Curves indicate fits of the probabilistic model under control conditions (dashed) and during optogenetic inactivation (cyan). Error bars show 95% binomial confidence intervals. Data were obtained in 6 sessions from 1 mouse. (C) Same as in (B), for inactivation of right visual cortex from the same mouse. Data were obtained in 7 sessions. (D) Decision variables obtained by the model fits in (B) as a function of contrast on the left and right in control condition (dashed) or during inactivation of left visual cortex (cyan). (E) Same as (D), for inactivation of right visual cortex. (F) Summary of the effects of optogenetic inactivation in the 4 regions outlined in (A). Effects are measured by the decrease in the left and right decision variables, $z_{L}$ or $z_{R}$, at 50% contrast. Dots indicate individual sessions from 2 mice (squares for the mouse in B–E, circles for another mouse) with inactivation of left visual cortex (red) or right visual cortex (pink). Crosses summarize the effects of inactivation in visual cortex (red and pink), and in somatosensory cortex (gray). The length of the crosses indicates ± SEM in the 2 dimensions. See also Figures S5 and S6.

To assess whether visual cortex was required for task performance, we silenced it optogenetically during individual trials (Figure 4A). We used 2 transgenic mice expressing channelrhodopsin-2 (ChR2) in *Pvalb*-positive inhibitory interneurons, implanted with clear skull caps (Lien and Scanziani, 2013, Guo et al., 2014b). We used a 473-nm laser to inactivate the left or right visual cortex (somatosensory cortex for control measurements) during visual stimulus presentation and wheel-turn responses. Electrophysiological measurements show that such inactivation was circumscribed to a radius of ∼1 mm (Figure S5).

Inactivation of visual cortex strongly suppressed the mouse’s ability to detect contralateral stimuli, but had little effect on the detection of ipsilateral stimuli (Figures 4B and 4C). To summarize these effects and compare them across experiments, we used the probabilistic model (Figures 4D–4F). In the example experiment, inactivating left visual cortex reduced only the decision variable for right stimuli (${\text{z}}_{\text{R}}$; Figure 4D), and inactivating right visual cortex reduced only the decision variable for left stimuli (${\text{z}}_{\text{L}}$; Figure 4E). Similar results were seen across experiments (Figure 4F): inactivating left visual cortex decreased ${\text{z}}_{\text{R}}$ by 2.9 ± 0.1, significantly more than ${\text{z}}_{\text{L}}$ (1.0 ± 0.2; paired t test, one-sided, p < 10^−5^), and inactivating right visual cortex decreased ${\text{z}}_{\text{L}}$ by 2.0 ± 0.2, significantly more than ${\text{z}}_{\text{R}}$ (0.5 ± 0.2; p < 10^−4^).

By comparison, in control experiments in which we inactivated the somatosensory cortex, we saw no such effects (Figures 4F and S6). Inactivating somatosensory cortex did not cause any significant change in decision variables (p = 0.17 and p = 0.25 for left and right somatosensory cortex; Figure S6). Indeed, the effect on the R decision variable was significantly weaker during inactivation of left somatosensory cortex than of left visual cortex (p = 0.00015, Wilcoxon rank-sum test). Similar effects were seen on the L decision variable following inactivation of right somatosensory versus visual cortex (p = 0.00012). We conclude that accurate performance on this task requires the visual cortex.

### Rewarding with Optogenetic Dopamine Stimulation

The conventional method to reward mice for performing perceptual decisions involves delivering fluids under conditions of water control. It would be ideal, however, if one could deliver reward without any water or food control. We sought to achieve this goal by stimulating brain centers that mediate the effects of positive reinforcement. We provided phasic optogenetic stimulation of midbrain dopamine neurons. Phasic stimulation of these neurons is known to be sufficient for simple behavioral conditioning, such as place preference, lever pressing or nose poking (Kim et al., 2012, Olds and Milner, 1954, Tsai et al., 2009). However, it is not known whether trial-by-trial stimulation of these neurons can act as an efficient reinforcer for perceptual choices.

**Figure 5:**
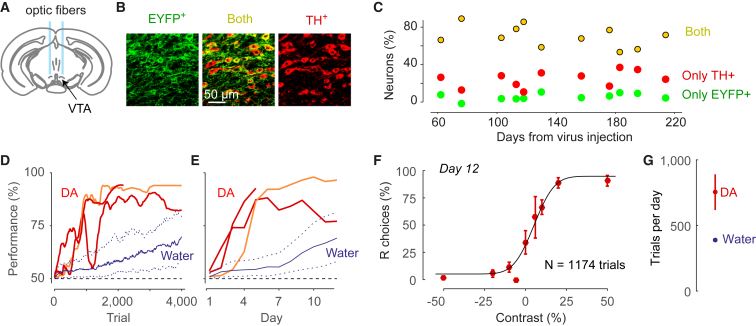
Using Optogenetic Phasic Dopamine Stimulation to Train Mice in the Task (A) Schematic coronal section of the mouse brain (at the bregma, 3.1 mm) showing ventral tegmental area (VTA) and fiber optics implanted above VTA to elicit release of dopamine (DA). (B) Confocal images showing expression of ChR2-EYFP (green) in TH^+^ (DA) neurons (red) and overlay showing both (yellow). The bars quantify the specificity of expression, showing statistics of ChR2-EYFP and TH^+^ expression in midbrain neurons (n = 1,460 neurons counted in 121 confocal images acquired from 11 mice). (C) Stability of ChR2 expression in DAergic neurons (n = 11 mice). (D) Rapid learning of the task in 3 mice receiving DA stimulation as a reward. Red and orange lines show rapid increase in the performance of naive mice that were solely trained with optogenetic DA stimulation. Blue curves show results for mice that trained with water reward (median and quartile ranges, replotted from Figure 1). (E) Same as in (D), as a function of training day. (F) Psychometric function obtained from example animal (orange line in C and D) on the 12^th^ day of behavioral training. Error bars show 95% binomial confidence intervals. (G) Mean trials per day of mice receiving DA stimulation (red) compared to water reward (blue). Error bars represent SEM (smaller than the dot for water reward).

We injected a viral construct containing Cre-dependent ChR2 into ventral tegmental area (VTA) and substantia nigra pars compacta (SNc) of DAT^IREScre^ mice, and implanted an optic fiber above VTA (Figure 5A). We confirmed specific expression of ChR2 in dopamine neurons using immunohistochemistry (Figure 5B). We identified dopamine neurons as those that stained for tyrosine hydroxylase (TH^+^). 71% of these neurons also expressed ChR2. On the other hand, only 5% of neurons that expressed ChR2 failed to react to TH staining, indicating that expression was highly selective to dopamine neurons. ChR2 expression was consistent across animals and was stable for months after virus injection (n = 1,460 neurons in 11 mice; Figure 5C).

We then trained 3 naive mice in our 2AFC task by reinforcing correct choices with only optogenetic dopamine stimulation and an associated click sound. Mice were not given water reward, and had free access to water in their home cage.

Mice trained with optogenetic dopamine stimulation rapidly learned the task, greatly outperforming animals trained for a water reward, both in learning speed and in number of trials per session (Figures 5D–5G). After only a few days of training with dopamine stimulation, mice often performed over 900 trials per session (in more than 50% of sessions), with high accuracy (>75%, Figures 5D and 5E), resulting in high-quality psychometric curves (Figure 5F). On average, mice rewarded with dopamine stimulation performed almost twice as many trials per session as those rewarded with water (Figure 5G). To assess the stability of dopamine stimulation as a means of providing reward, in one mouse we continued these measurements for 10 weeks, during which the method remained robust.

The click sound at the onset of the optogenetic stimulation may be important for the success of these experiments for two reasons. First, when we attempted to train a mouse with optogenetic stimulation but no click sound, the animal did not learn the task. Second, it is known that sensory stimuli can be powerful secondary reinforcers (Herrnstein, 1964), and click sounds are particularly effective in “clicker training” (Pryor, 1999).

### Stimulus Discrimination

A method for performing psychophysics should be flexible, so that it can be altered as needed. For instance, the basic tasks that we have described, whether 2AFC or 2AUC, involve detecting the position where a stimulus appears, either on the left or on the right. To study the mechanisms that combine information across hemispheres, however, it is useful to have the subject discriminate between stimuli that appear on both sides, as in contrast discrimination tasks commonly used with human observers (Boynton et al., 1999, Legge and Foley, 1980, Nachmias and Sansbury, 1974).

**Figure 6:**
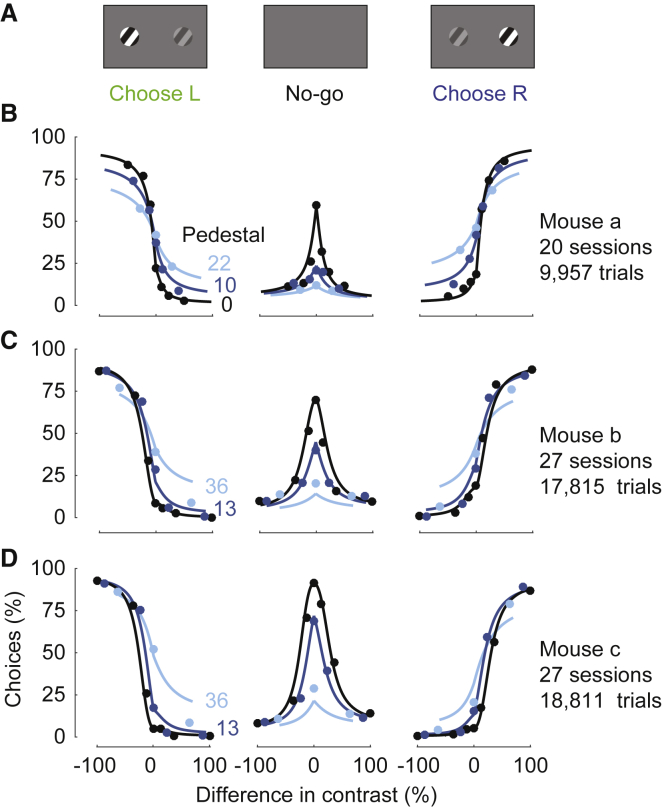
Extension of the 2AUC Task to the Study of Contrast Discrimination (A) Stimulus conditions used in the discrimination task. Gratings are presented on both sides and the mouse is rewarded for choosing the side with the highest contrast, or opting for no-go if both contrasts are zero. (B) Psychometric data from 1 mouse. Panels show left choices, no-go choices, and right choices, as a function of the difference between left and right contrast (c_R_-c_L_). Colors indicate the pedestal contrast, i.e., the minimum contrast present on the screen, min(c_L_,c_R_). (C and D) Same as in (B) for 2 more mice.

Mice that had already learned 2AUC contrast detection readily learned to perform contrast discrimination (Figure 6). In most trials, 2 stimuli appeared on the screen, and mice were rewarded with water for selecting the stimulus with higher contrast (Figure 6A). A no-go response was rewarded only when no grating was presented on either side. If contrasts were nonzero and equal, mice were rewarded randomly with 50% probability for left or right responses. Mice learned this task generalization, yielding high-quality psychometric curves (Figure 6B). When both gratings were present (a positive “pedestal contrast,” Legge and Foley, 1980), mice correctly gave fewer no-go responses, while finding it harder to indicate the side with higher contrast (Figures 6B–6D). Their decisions conformed closely to the predictions of the probabilistic observer model (Figure 3). With a fixed setting of its 6 parameters the model provided satisfactory fits to the 32 response probabilities measured across 3 pedestal contrasts.

These results illustrate this task’s suitability for bringing to the mouse methods that are traditional in human visual psychophysics. These can be useful both to probe mouse vision and to relate perceptual decisions to neural activity.

## Discussion

We describe a flexible task for assessing visual decision-making in head-fixed mice. The steering wheel allows mice to accurately report one of two alternative stimuli, and the task is readily extended to allow a no-go response option. The task is learned quickly and reliably: most mice master it within a few weeks. The task yields a large number of trials per session, providing high-quality psychometric curves within individual sessions.

Mice are head-fixed, which facilitates not only brain recordings and manipulations but also careful control of visual stimulation and measurement of eye position. Mice sometimes moved their eyes during the same epochs as wheel turns, and these eye movements would correlate with neural activity. Tracking these behaviors and understanding their relationship with neural activity is an important control and an interesting direction for further research.

The decisions made by mice in this task follow the predictions of a simple probabilistic observer model. We formulated the model in terms of log odds (multinomial logistic regression), inspired by an earlier formulation based on signal detection theory (Sridharan et al., 2014). Both formulations are two-dimensional: responses depend on the combination of two decision variables. This is essential to capture the effects of unilateral inactivation, which would not be captured by models with a single decision variable (García-Pérez and Alcalá-Quintana, 2011, García-Pérez and Alcalá-Quintana, 2013, Kiani and Shadlen, 2009). Our formulation has two advantages over the earlier one (Sridharan et al., 2014). The first is technical: having a functional dependence on stimulus contrast minimizes free parameters. The second has broader import: by recasting the model as a logistic regression, it is easier to modify the analysis to include other predictors such as choice history (Abrahamyan et al., 2016, Bak et al., 2016, Busse et al., 2011, Licata et al., 2017) or neural activity (Nienborg and Macke, 2014). Including a neural signal as a predictor provides a means to assess whether that signal is informative of the animal’s decisions.

We also demonstrated that transient optogenetic dopamine stimulation is sufficient for mice to learn a perceptual decision task. The combination of our task and dopamine stimulation may be useful for studying the effects of dopamine signals on perception and perceptual learning (Ding and Gold, 2013, Schultz et al., 1997). Our results show that dopamine stimulation is an attractive alternative to water reward, accelerating task acquisition and almost doubling trial counts. A large number of trials are particularly useful when relating perceptual decisions to neural activity. Moreover, the method is arguably less disruptive of normal mouse behavior and physiology, as it does not constrain water intake.

As currently implemented, however, our optogenetic method also carries limitations. First, it requires the use of DAT-Cre mice, which may not be feasible if Cre needs to be expressed in other cells for other experimental purposes. Second, it requires implantation of optic fibers, which take up valuable space on the mouse head.

An advantage of our task is that it is highly flexible, allowing for many extensions of the same basic design. We have modified the task depending on requirements, for example introducing a cue informing mice when to respond, and a no-go response option to report stimulus absence. We exploited this no-go response in inactivation experiments, finding that inactivation of visual cortex diminished reports of contralateral stimuli but left ipsilateral reports unaffected. We also modified the task in a variant requiring contrast discrimination between two stimuli, generating high-quality psychometric functions that were modulated by contrast difference and by the pedestal contrast. We also found that, once trained, mice continue to perform if the stimulus position is fixed or is only transiently presented, which can be exploited to address concerns about stimulus movement being related to choice, or of presentation duration being controlled by the mouse.

We believe that the coupling of wheel movements to stimulus properties is a particularly useful learning aid, and is further generalizable. For example, the task can be extended beyond the detection or discrimination of visual contrast. In preliminary results (data not shown), we have trained mice to use the wheel to rotate a grating to a target orientation or to modulate repeated tones toward a target pitch.

Moreover, the continuous readout available from the steering wheel may provide further insight into the nature of behavior. We used the wheel to obtain discrete reports, but the continuous readout may afford more sensitive assays, probing factors such as motivation, confidence (Lak et al., 2014), response vigor, and vacillation (Resulaj et al., 2009). These considerations suggest additional extensions of the task to a fully interactive, flexible, and accurate platform for probing mouse vision and visuomotor behavior and establish their neural basis.

## Experimental Procedures

All experiments complied with the law governing animal research, i.e., the Animals (Scientific Procedures) Act 1986 Amendment Regulations 2012, in the United Kingdom. Procedures were approved by the local Animal Welfare Ethical Review Body and by the Home Office (license 70/8021). Detailed methods are described in the Supplemental Experimental Procedures.

To allow head-fixing, mice (male and female, aged 8–24 weeks) were first anesthetized and implanted with metal head-plates. After at least 4 recovery days, mice were acclimatized with head-fixing and then trained in a simplified version of the task involving only stimuli with high contrast and no timing requirements. As performance improved, lower contrasts and more stringent timings were introduced. Training criteria were qualitative and differed across experimenters and mice.

Most mice were trained using water as a reward. After the task, they received top-up fluids to achieve a minimum daily amount of 40 ml/kg/day. Body weight and potential signs of dehydration were monitored daily.

Stimuli were presented on 1 LCD monitor or on 3 monitors placed around the animal. Intensity values were linearized with a photodiode. In some experiments, we covered the monitors with plastic Fresnel lenses to make intensity spatially uniform. The response wheel was a Lego rubber tire, whose angle was measured using a rotary encoder. A detailed parts list is available at http://www.ucl.ac.uk/cortexlab/tools/wheel.

Stimuli were typically sinusoidal gratings in a Gaussian window, but the specifics of this stimulus generally differed by mice. To measure pupil position and dilation, we used a camera focused on one eye, illuminated by an infrared LED, and fitted a 2D ellipse to the pupil.

Imaging was performed in three 10- to 12-week-old C57BL/6J female mice. During the initial surgery, we performed a craniotomy centered on the right primary visual cortex and injected a GCaMP6m virus (AAV2/1-*syn*-GCaMP6m-WPRE). We sealed the craniotomy with coverslips and dental cement. We began calcium imaging 3 weeks after virus injection. Imaging was performed using a Sutter two-photon microscope controlled by ScanImage, with a Coherent Chameleon laser (1,000 nm) and Olympus 20× objective. We chose a field of view with good GCaMP expression and mapped the preferred stimulus position of the field of view, using this for the position of the task stimulus during behavior. We registered the raw calcium movies by aligning each frame to a reference frame and found neurons through a semi-automated algorithm that selected nearby pixels significantly correlated with each other. We obtained a baseline *F*_*0*_ by smoothing the calcium trace *F* in time and finding the minimum over a 20-s sliding window. We then computed Δ*F/F* by applying a causal exponentially weighted filter (τ = 0.2 s) to the fractional change *(F − F*_*0*_*)/F* (Jia et al., 2011).

To characterize psychometric performance in the 2AFC task, we calculated the proportions of trials with rightward choices (ignoring repeat trials that were sometimes introduced after errors) and fitted them with a psychometric function (e.g., Busse et al., 2011).

To measure task performance as a function of trial number, we considered easy trials (contrast ≥ 40%) and estimated the probability of a correct response as a function of trial as well as its confidence intervals (Smith et al., 2004). Daily performance was estimated by averaging across each day’s easy trials.

In the 2AUC version of the task, the mouse was required to be still for 0.5–1 s after stimulus onset. This period of no movement was followed by an auditory go cue. Lack of movement within 1.5 s of the go cue was considered a no-go response, which was met with a reward for trials with zero contrast stimuli or with a 2-s white noise burst for all other stimuli. We trained mice in this 2AUC version by first training them in the 2AFC version (at least with high contrast) and then introducing zero contrast (no-go) trials.

To fit 2AUC data, we used the model in Equations 1, 2, and 3. We fit the 4 parameters of Equation 2 through multinomial logistic regression and optimized the 2 parameters in Equation 1. When measuring the effects of inactivation, we fitted the different inactivation conditions independently, while holding constant the parameters of Equation 1. This allowed us to capture the effects of inactivation with changes in the parameters of Equation 2.

Inactivation experiments were performed with mice expressing ChR2 in *Pvalb*-positive inhibitory neurons (B6;129P2-*Pvalb*^*tm1(cre)Arbr*^/J crossed with Ai32). Mice were prepared with a clear skullcap similar to that used by Guo et al. (2014b) but with UV-curing optical adhesive. Inactivation light was produced by a 473-nm diode laser coupled to a fiber, producing ∼1.5 mW in a spot of ∼0.3 mm diameter, positioned over visual cortex (3.3–3.7 mm posterior, 2.1 mm lateral) or somatosensory cortex (0.8 mm posterior, 2.5 mm lateral). Inactivation was performed randomly in ∼30% of trials. Light was delivered as a 40-Hz sinusoid beginning 33 ms before visual stimulus onset and lasting until the response. The task was 2AUC detection, but responses could be immediately made on stimulus onset.

For optogenetic dopamine stimulation, we used DAT-Cre mice (Jax 006660) backcrossed with C57BL/6J mice. We injected 1 μL of diluted virus (AAV5.EF1a.DIO.hChr2(H134R)-eYFP.WPRE) into VTA and SNc and implanted an optic fiber with tip 0.5 mm above the injection site. We waited 3 weeks for virus expression and then started behavioral training. On making a correct choice, animals received a short train of laser stimulation (473 nm, 12 pulses each lasting 10 ms and separated by 40 ms, power 10–15 mW measured at the fiber tip) and a simultaneous click sound.

To quantify ChR2 expression in dopamine neurons, 50-μm coronal sections were collected and immunostained with antibodies to EYFP and TH and secondary antibodies labeled with Alexa Fluor 488 and 594 (Tsai et al., 2009).

The contrast discrimination task is based on the 2AUC task, but gratings could be presented on both sides of the screen simultaneously, and mice were rewarded for choosing the grating with the highest contrast. Mice were first trained in the 2AUC detection task, and discriminations were introduced incrementally. Mice learned this discrimination task within a few days after learning the detection task.

## Author Contributions

Conceptualization: C.P.B., A.L., N.A.S., P.Z.-H., J.F.L., K.D.H., and M.C. Methodology: C.P.B., A.L., N.A.S., P.Z.-H., S. Schröder, S. Soares, J.J.P., L.E.W., K.D.H., and M.C. Software: C.P.B., A.L., N.AS., and P.Z.-H. Formal Analysis: C.P.B., A.L., N.A.S., P.Z.-H., and M.J.W. Investigation: C.P.B., A.L., N.A.S., P.Z.-H., C.B.R., E.A.K.J., A.R., S. Schröder, and M.J.W. Writing – Original Draft: C.P.B., A.L., N.A.S., P.Z.-H., and M.C. Writing – Reviewing and Editing: C.P.B., A.L., N.A.S., P.Z.-H., E.A.K.J., J.J.P., S. Schröder, S. Soares, L.E.W., K.D.H., and M.C. Funding Acquisition: C.P.B., A.L., N.A.S., S. Schröder, E.A.K.J., K.D.H., and M.C. Supervision: K.D.H. and M.C. Project Administration: M.C.

## Supplemental Information

Document S1. Supplemental Experimental Procedures and Figures S1–S6

Movie S1. A Mouse Performing the Two-Alternative Unforced-Choice Contrast Discrimination Task The inset to the main panel shows the image taken by the eye camera. The panel below the main panel illustrates the images that appear on the three screens around the animal.

Document S2. Article plus Supplemental Information
