# Supplementary material for: High-Yield Methods for Accurate Two-Alternative Visual Psychophysics in Head-Fixed Mice
Source: Cell Rep. 2017 Sep 5;20(10):2513–24. doi: 10.1016/j.celrep.2017.08.047 (PMC5603732; doi:10.1016/j.celrep.2017.08.047)
Supplement: Document S1. Supplemental Experimental Procedures and Figures S1–S6 [file mmc1.pdf]

Cell Reports, Volume *20*

Supplemental Information

High-Yield Methods for Accurate Two-Alternative Visual Psychophysics in Head-Fixed Mice

Christopher P. Burgess, Armin Lak, Nicholas A. Steinmetz, Peter Zatka-Haas, Charu Bai Reddy, Elina A.K. Jacobs, Jennifer F. Linden, Joseph J. Paton, Adam Ranson, Sylvia Schröder, Sofia Soares, Miles J. Wells, Lauren E. Wool, Kenneth D. Harris, and Matteo Carandini

Supplementary Figures

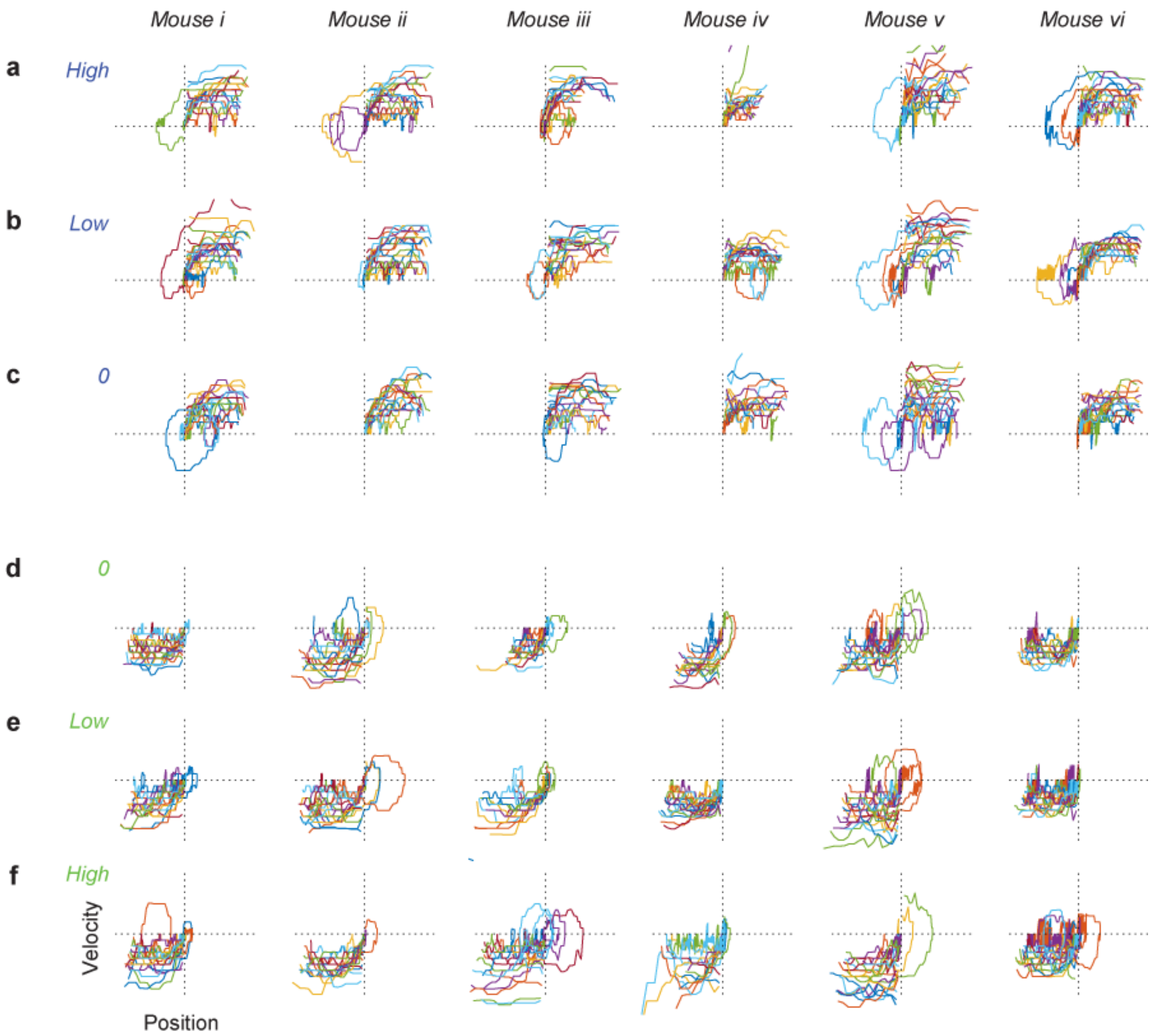

Supplementary Figure 1. Trajectories of wheel turns made by mice in response to stimuli. Related to Figure 1. Traces show evolution of position and velocity during trajectories for turns made between stimulus onset and attainment of choice threshold.

a-**c**: Trajectories that ended with a choice to the left, for stimuli that had high contrast on the left (a), low contrast on the left (b), or zero contrast (c). Any trials where the initial choice direction is inconsistent with the final choice must cross from one quadrant to the other (lower-left to upper-right), which is uncommon.

d-**f**: Same as **a**-**c**, for trajectories that ended with a choice to the right.

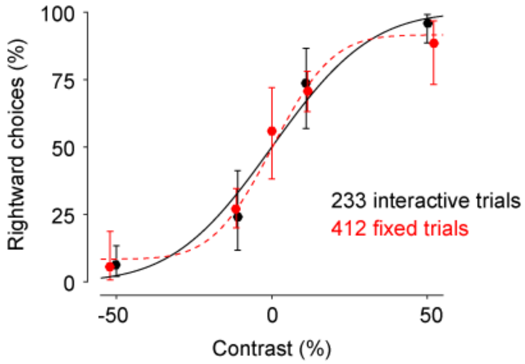

Supplementary Figure 2. Comparison of psychophysical performance in interactive trials vs. fixed-stimulus trials. Related to Figure 1.These data were obtained in a single session in which two types of trial were randomly interleaved. In normal interactive trials, the steering wheel moved the stimulus (black). In the remaining trials, the mouse completes choices by turning the wheel as normal, but the stimulus remains fixed at the onset position (red). The ordinate plots the percentage of times the mouse chose the stimulus on the right (R), as a function of stimulus contrast (positive for R stimuli, negative for L stimuli). The psychometric curves fit across the two sets of trials (curves) are similar.

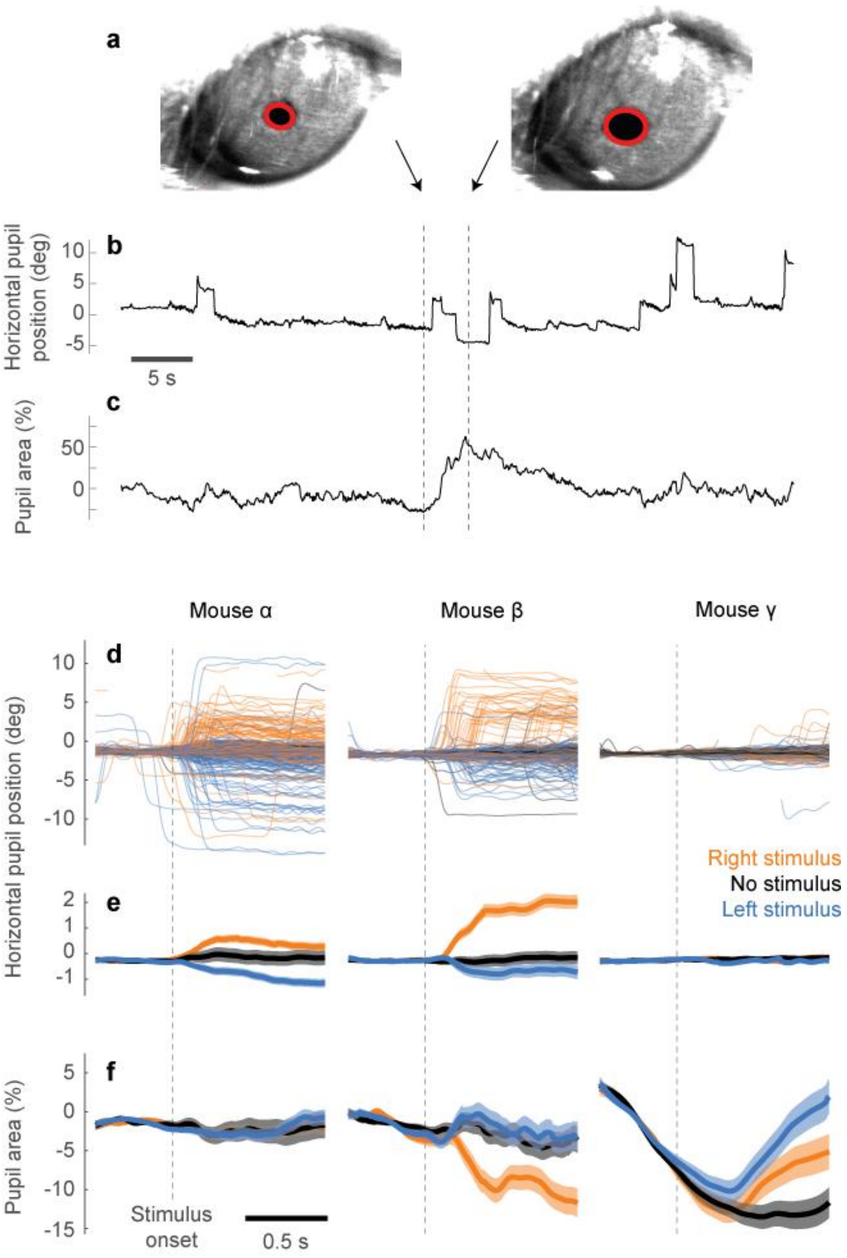

Supplementary Figure 3. Eye movements during task performance. Related to Figure 1.

a: Two example frames showing ellipses fit to the pupil.

b: Example traces of horizontal position calculated from movies of the eye. Saccades as small as ~2 deg are clearly visible. Dashed lines indicate the times of the two frames in **a**.

c: Same as **b**, for the pupil area (proportion change relative to the mean).

d: Traces of pupil position for each trial from three example mice. Traces are aligned to stimulus onset and colored according to stimulus condition: stimulus on left (blue), right (orange), or no stimulus (black).

e: Average of the traces in **d**. Notice different y-scale. Shaded area represents two s.e.m.

f: Same as **e**, for the pupil area.

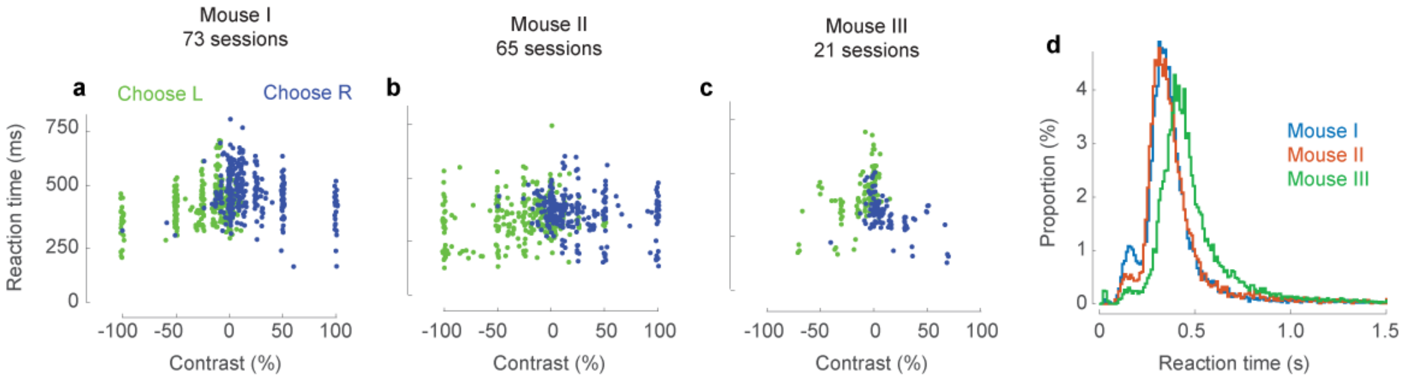

Supplementary Figure 4 Reaction times in the 2AUC task. Related to Figure 3.

a-c. Median reaction time for the example mice in Figure 3. Each dot indicates the median reaction time measured for stimuli of a given contrast in a single session, grouped by decisions made on the left (*green*) or on the right (*blue*).

d: Distribution of reaction times for all trials in the three mice. The standard deviations of the reaction time distribution were 206, 181, and 198 ms respectively. The proportions of trials with reaction times longer than 1 s were 3.6%, 2.1%, and 2.8% respectively. The vast majority of reaction times, therefore, are much shorter than the 1,500 ms that would result in a No-go response

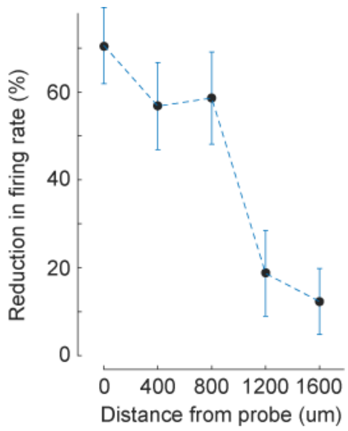

Supplementary Figure 5. Control electrophysiological measurements show optogenetic inactivation of visual cortex was spatially focused, with a radius of ~1 mm. Related to Figure 4. We inserted custom multisite electrodes in visual cortex, and pooled responses from n = 110 single-unit and multiunit clusters with broad waveforms. We moved the laser at different distances from the electrode (abscissa) and measured the reduction in firing rate relative to control firing rate (modulation index, ordinate). The spot size and laser power (1.5 mW) were the same as in the behavioral experiments.

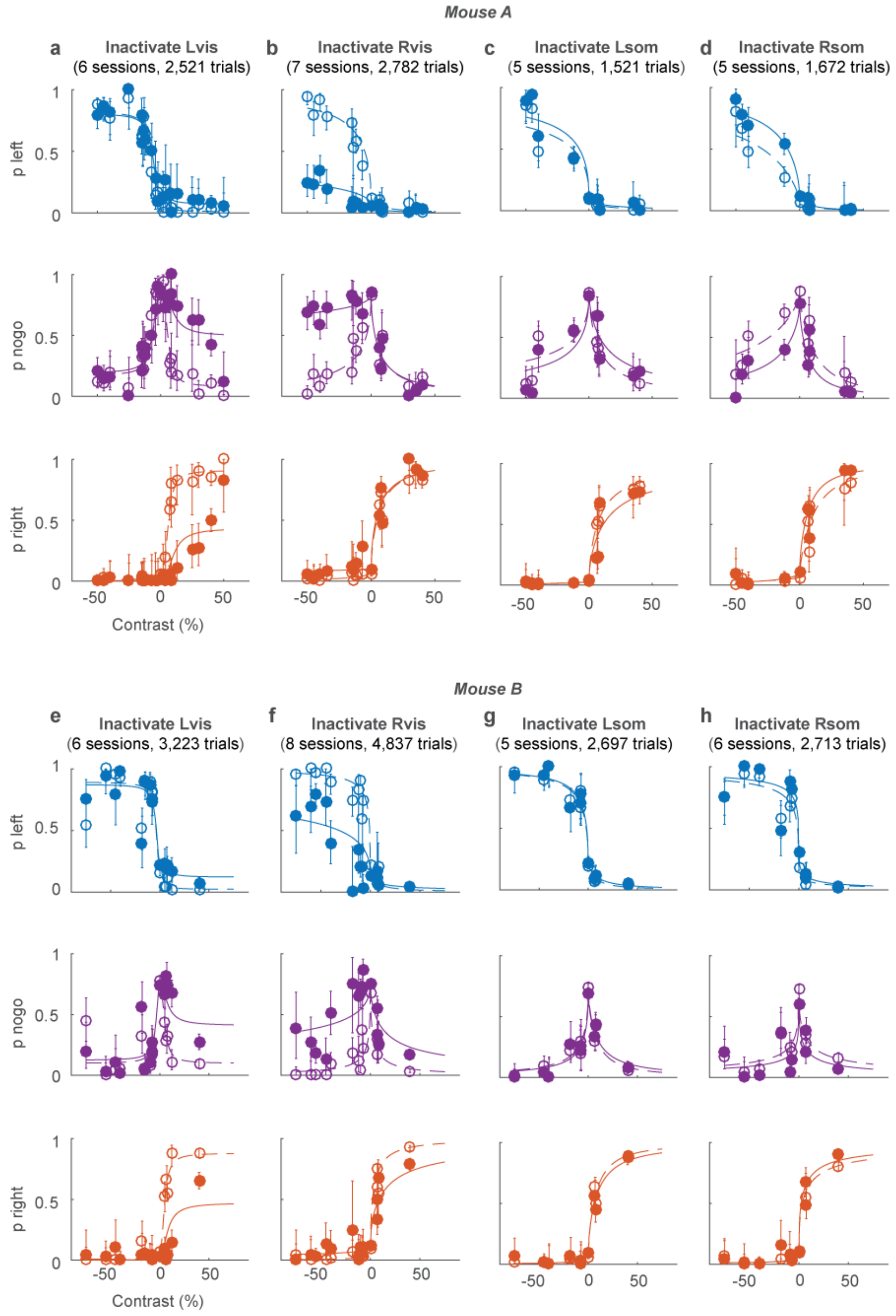

Supplementary Figure 6. Results of inactivation in 4 regions in two mice. Related to Figure 4.

a,**b**: Effects of inactivation of left and right visual cortex in mouse A (same data as Figure 4**b**,**c**).

c,**d**: Effects of inactivation of left and right somatosensory cortex in mouse A.

e-h: Same as **a-d**, for mouse B.

Supplemental Experimental Procedures

All experiments were conducted according to the UK Animals Scientific Procedures Act (1986). Male and female mice between the ages of 8-24 weeks were used for all experiments. Mice were C57BL/6J or transgenics with a C57BL/6J background. Measurements were made during the day (9 am to 8 pm). The daylight cycle for the mice was normal (8 am - 8 pm) except for some mice in the basic task (Figure 1), where it was inverted (9 pm – 9 am). We did not investigate the effect of daylight cycle on performance. Mice were housed on their own or in pairs.

Head-plate implant

Mice were implanted with metal plate on the cranium to enable their heads to be fixed. To perform this surgery, mice were injected with an anti-inflammatory drug (4 mg/kg Carprofen, subcutaneously) and anaesthetized using isoflurane (1–2%). Body temperature was maintained at 37°C using a heating pad and the eyes were protected with artificial tears to prevent drying (Viscotears). The head - plate was implanted chronically by fixing it to the cranium with dental cement (Sun Medical). After surgery, mice were allowed at least 4 days to recover before water control and behavioral training began.

Apparatus

The response wheel was a Lego part with a rubber tire (a cylinder 19 mm wide and 31 mm in diameter). Its angle was measured using a rotary encoder (typically, a Kübler 05.2400.1122.0100, with resolution 0.9° or about 0.5 mm of wheel circumference) whose signal was acquired using a data acquisition device (National Instruments USB-6212). Water was dispensed by opening a solenoid valve (Neptune Research 161T011) for a calibrated duration of time.

Stimuli were presented on an LCD monitor (refresh rate 60 Hz) placed in front of the animal. Monitor intensity values for each color channel were linearized by using measurements from a photodiode. This procedure, however, was generally carried out in experimental rigs but not in training rigs. Moreover, LCD panels are difficult to linearize because intensity varies strongly with viewing angle: if the line of sight is orthogonal to the screen at the center of the screen, the sides of the screen, and especially the corners, are substantially darker. Only towards the end of this project we realized how to overcome this difficulty: by placing plastic Fresnel lenses in front of the screens.

The initial apparatus used for these experiments involved multiple parts custom-built by a machine shop. Later versions rely entirely on off-the-shelf components and 3D-printed parts. The design for the latest version, together with a detailed parts list, is described at www.ucl.ac.uk/cortexlab/tools/wheel.

The task was managed by custom MATLAB software, through an open-source package called Signals (github.com/dendritic/signals). It uses a dataflow-style paradigm to allow concise and intuitive specification of stimulus presentation, task structure, and control of data acquisition. To control graphics presentation for visual stimulation, we used the Psychophysics Toolbox (Brainard, 1997; Pelli, 1997).

Stimuli were typically Gabor patches, i.e. sinusoidal gratings (typically, vertical, with wavelength 10°) in a Gaussian window. The Gaussian typically had standard deviation of ~10°. However, there was great variation across mice in these and other parameters, including position. In different mice we generally used different visual stimuli (different spatial frequency, size, temporal frequency, position, etc.) and all these factors contribute to the visibility of the stimuli. A future study could vary these attributes in a controlled manner and use our techniques to measure properties of mouse vision.

Training procedure

Before training, mice were acclimatized daily with being handled and with being head-fixed in the steering wheel rig, with its forepaws resting upon the wheel. The mouse was able to turn the wheel with left or right movements of its forepaws. It was able to consume droplets of water dispensed via a spout close to its mouth. This acclimatization phase lasted for 3 days, with the duration of restraint gradually increasing from 10-30 min in the first day to up to 3 hours in the third day.

Mice were then trained in the task typically in daily one -hour sessions over a period of weeks. During the first few sessions mice were trained on a simplified version of the task, with 100% or 50% contrast, no inter-trial delays, quiescent period, or open loop period. Once they began to start turning the wheel in both directions, the delays were increased to their final values. Once performance was

above chance level, lower contrasts were gradually introduced. Typically, mice were running on the final task parameters by week 2-3.

The default distance to move the wheel at the start of training was ~2 cm, or about a 45° turn. This was adjusted during training when it appeared that a mouse made a consistent movement in the correct direction but the movement was too short. This could happen, for instance, if the mouse's position relative to the wheel was inadvertently set differently one day than on previous days.

In this study, however, we used no quantitative criteria for advancement from one stage to the next. Different experimenters used different methods based on personal intuition and experience. For instance, some experimenters found it useful to move the wheel by hand in occasional trials on the first day of training, in case the mouse was making no effort to turn it on its own. Moreover, to break possible patterns of stereotyped responses, many experimenters introduced "correction trials": if an animal failed to give the correct response to a high-contrast stimulus, the stimulus was presented in the same location in all subsequent trials until the animal gave the correct answer. The responses given during correction trials were not used to calculate psychometric curves or fit the probabilistic model. Some experimenters also found it useful to provide stimuli more frequently on one side than on the other, to correct for side biases (i.e. provide more stimuli on the side where the mouse performs worse). In a future study, it would be useful to develop an automated training schedule, perhaps using the quantitative measures of performance shown in Figure 1 (which were made post-hoc, not during training), and perhaps tailoring the stimuli to defeat superstitious strategies that weigh past decisions and outcomes (Abrahamyan et al., 2016; Bak et al., 2016; Busse et al., 2011; Licata et al., 2017).

Most mice were trained using water as a reward. They were placed on a water control schedule in which they received a minimum daily amount of 40 ml/kg/day (1 ml/day for a typical 25 g mouse). For this purpose, however, it would not be appropriate to use the mouse's actual weight, because animals on water control tend to lose weight. Similarly, it would not be appropriate to use the weight on the first day of training, because animals grow with age. We thus estimated the weight that the animal would have had if it had not been on water control. To do this, we weighted the mouse on the first day of the water control schedule and referenced this weight to a standard curve *W(sex,age)* relating sex and age to body weight in animals that are not on water control (www.jax.org/jax-mice-and-services/strain-data-sheet-pages/body-weight-chart-000664). This procedure established that the mouse's weight was a fraction x of the mean of other mice of same sex and age (with $x<1$ or $x>1$ for mice lighter or heavier than average). From then on, the mouse's age- and animal-adjusted weight was taken to be $w = x\ W(sex,age)$. The minimum required water was estimated based on w.

The mouse was then weighted again on each training/testing day (typically 5-7 days/week), and signs of dehydration were monitored: skin tension, sunken eyes, and marked variations in general behavior (no mice showed any of these signs). The animal spent at most 3 hours/day in training/testing, typically in a single session/day (occasionally, two sessions/day). At the end of each session, the amount of water received was logged by software and controlled visually by the experimenter. At the end of the day, the animal received top-up fluids (in the form of appropriately weighted Hydrogel packages, to prevent accidental spilling and to minimize the perceived equivalence to the fluids received during the task) to ensure that it received the minimum daily amount. On days in which no training/testing took place, the mouse received the entire minimum daily amount in the form of Hydrogel. If the mouse weight dropped below 80% of the age- and animal-adjusted weight w, the minimum daily amount was increased, and if the weight dropped below 70% of w (a very rare event), the animal was given ad-lib water until weight recovered. Similarly, if signs of dehydration had ever been positive, the mouse would have been placed on ad-lib water until recovered.

Task reward was also calibrated throughout the training process. When mice were naïve and did few trials they would be given more per correct trial (~3 µL), and as they became proficient and were completing >300 trials they would typically be given ~2 µL.

Eye tracking

On many sessions (typically imaging, inactivation, and some training sessions) we recorded eye position. We used a camera (DMK 21BU04.H or DMK 23U618, The Imaging Source) with a zoom lens (ThorLabs MVL7000) focused on one of the eyes. When fully zoomed and placed ~20 cm from the mouse, this setup provided ~73 pixels/mm. To avoid contamination of the image by reflected monitor light relating to visual stimuli, the eye was illuminated with a focused infrared LED (SLS-0208A, Mightex; driven with LEDD1B, ThorLabs) and an infrared filter was used on the camera (FEL0750, ThorLabs; with adapters SM2A53, SM2A6, and SM1L03, ThorLabs). We acquired videos with MATLAB's Image Acquisition Toolbox (MathWorks).

For each video frame, we determined pupil size and location with the following steps: 1) Smooth the image with a 2D Gaussian filter of manually-selected width; 2) Manually select an intensity threshold that discriminates between pixels inside vs. outside the pupil; 3) Find the contour corresponding to this intensity value; 4) Fit a 2D ellipse to this contour by minimizing the mean squared error of:

$$Ax_i^2 + Bx_iy_i + Cy_i^2 + Dx_i + Ey_i = 1$$

where $(x_{i,}y_i)$ are coordinates of points on the contour. Pupil area and center position were calculated directly from this fit ellipse. Frames for which no contour could be detected or for which the fit ellipse was outside the range of possible values (typically due to blinks or grooming) were assigned NaN values. Relative pupil area A was then quantified as a proportion change relative to mean: $A = (a - \hat{a})/\hat{a},$ where a is the absolute area (in pixels) and $\hat{a}$ is the mean area across all frames. Position values were converted to deg of visual angle α by first converting from pixels to mm, then assuming that the pixel center moved on the surface of a sphere: $\alpha = 360m/\pi d$. Here, m m is the position in mm and d is the diameter of the eye. We did not measure this diameter but rather assumed it to be the customary $d = 3.4$ mm (Remtulla and Hallett, 1985).

Imaging V1 responses

The imaging experiments were performed in three 10-12 week old C57BL/6J female mice. During the initial surgery, in addition to implanting the head -plate we performed a 1 mm^2 craniotomy in the middle of a circular aperture in the head -plate. The craniotomy was centered in the right primary visual cortex. We then injected them with a GCaMP6m virus under the human synapsin promoter (AAV2/1-*syn*-GCaMP6m-WPRE, 50 nL undiluted $2x10^{13}$ genome copy/ml) from Penn Vector Core (Chen et al., 2013) into the center of the craniotomy (stereotaxic coordinates 2.8 mm lateral and 3.3 mm caudal to Bregma) at a depth of 250 µm beneath the dura. We then covered the craniotomy with a two-layer glass coverslip construction, and sealed it with dental cement. The mice were allowed to recover for 1 week before water control and head-fixed training began.

We began calcium imaging 3 weeks after virus injection. Imaging was performed using a Sutter two-photon movable objective microscope controlled by ScanImage (Pologruto et al. 2003). A Coherent Chameleon laser running at 1000 nm provided excitation, with power level controlled by a Conoptics Pockels cell. Images were acquired continuously at 12 Hz with a resolution of 128×128 pixels. An Olympus 20X objective was used for focusing. Imaging data was synchronized with behavioral and stimulus events by simultaneously acquiring signals with imaging frame events and screen refresh events. The latter were measured using a photodiode directly measuring the screen.

In each mouse, we chose a field of view with good GCaMP expression and mapped the preferred stimulus position of the field of view by repeatedly presenting a grating stimulus on a gray screen for 1 s with 1-2 s inter-stimulus intervals. Stimuli were presented at random positions in a 5×5 grid in the left hemifield. The mean stimulus response across the field of view was calculated at each stimulus position. The position evoking the largest response was taken as the field of view's position preference. Before behavioral imaging commenced, we shifted the position of the task stimulus to the preferred position of the chosen field of view (the shift was typically < 10°). Stimulus orientation and size were not optimized.

We first registered the raw calcium movies using an algorithm that aligns each frame to the peak cross-correlation with a reference frame using the discrete Fourier transform (Guizar-Sicairos et al., 2008). We found cell regions of interest (ROIs) by using a semi-automated algorithm that selected nearby pixels that are significantly correlated with each other. $\Delta F/F$ calcium signals of ROI traces were computed as in Jia et al. (2011). Briefly, from calcium traces F, we obtained a measure of baseline F_0 by smoothing F in time (0.75 s causal moving average) and finding the minimum over a (causal) sliding window (20 s). $\Delta F/F$ is computed by applying a causal exponentially weighted filter (τ = 0.2 s) to the fractional change $(F-F_0)/F$.

Measures of 2AFC performance

To characterize psychometric performance in the 2-alternative forced-choice task (2AFC, Figure 1**c**, Figure 2**a**,**e**, and Figure 5**e**) we fitted a classical psychometric functions of contrast. We calculated the proportion of trials with rightward choices (ignoring repeat trials that were sometimes introduced after errors), and we fitted them with a standard psychometric function (e.g. Busse et al., 2011):

$$\Psi(c) = \lambda + (1 - 2\lambda)Erf(\frac{c - \mu}{\sigma})$$

where c is signed stimulus contrast (positive values for stimuli on the right, negative for stimuli on the left), and Erf is the cumulative Gaussian function. The parameters μ and σ are the bias and slope of the psychometric function, and λ is the lapse rate, i.e. the fraction of trials that are guessed independently of contrast. In this formulation, we used similar lapse rates for left and right choices. In other cases it was preferable to allow two different biases. We performed the fitting via maximum likelihood estimation, using the MATLAB function *fminsearch* over the log likelihood function.

To measure task performance as a function of trial number (Figure 1**d**,**e**) we used the model of Smith et al. (2004). This model prescribes a state-space smoothing algorithm to characterize a learning curve (probability of a correct response as a function of trial) and its confidence intervals. We applied this analysis to easier (contrast ≥ 40%) trials. Daily performance was estimated by taking the mean performance across each day's trials. This procedure was performed after the experiments, to analyze performance, but could in principle also be integrated into an automated system that advances the mouse to subsequent training stages based on estimates of learning.

2AUC version

In the 2AUC version of the task we did not use an auditory cue at stimulus onset, and the mouse was required to be still for 0.5-1 s after stimulus onset. This period of no movement was followed by an auditory Go cue (12 kHz pure tone lasting 100 ms with a 10 ms onset and offset ramp (Figure 3**b**). If the animal did not respond within 1.5 s of the go cue, this was considered a No-go response. No go responses were rewarded for trials with zero contrast stimuli or were met with a 2 s white noise burst for all other stimuli.

Zero-contrast stimuli were presented in ~20% of the trials. A series of ~5 consecutive No-go responses drew the attention of the experimenter. If the animal had stopped turning the wheel even following high-contrast stimuli, this was taken to indicate that the session was finished.

Training mice in this 2AUC version was done by first training them in the 2AFC version (at least with high contrast), and then introducing zero-contrast (No-go) trials. This was done only after the mouse's reaction times were mostly <1 s (so that, if there really is a stimulus, the mouse responds in time). In 3 of 37 mice (8%), reaction times stayed too long so we did not attempt to train the 2AUC version. No-go trials were repeated when incorrect but the repeats are not included in further analyses.

Of 34 mice trained on the 2AUC task, five (15%) initially had difficulty with the No-go trials, and this difficulty suggested that they monitored only one side of the screen. Indeed, these mice chose the ignored side (instead of giving a No-go response) also when there were no stimuli. To overcome this difficulty, we typically increased the proportion of zero-contrast trials and of trials with stimulus on the ignored side, even to the point of entirely removing trials with stimuli in the monitored side. Once performance improved, we progressively rebalanced the stimulus presentation. This approach worked well in 4 of the 5 mice with this initial difficulty. In the fifth mouse, a major bias persisted even after 15 sessions, and training was abandoned.

In general we presented stimuli with probability 1/3 to appear on the left, 1/3 to appear on the right, and 1/3 to be zero contrast (requiring a no-Go response). In some cases, we wished to reduce the number of no-Go responses, so that the mice would incorrectly choose left or right when in fact the stimulus was absent. We achieved this by reducing the proportion of zero-contrast stimuli. This made the mouse less likely to give a no-Go response.

Fits of the probabilistic model

To fit 2AUC data (Figure 3, Figure 4, and Figure 6) we used the probabilistic model defined in Equations 1-3. We fit the 4 parameters of the decision variables (Equation 2) to the data obtained in individual sessions through multinomial logistic regression, and optimized the additional two parameters describing contrast sensitivity (Equation 1). The resulting model has 6 parameters. For the data in Figure 3, these values are listed in Table 1.

	b_L	b_R	s_L	s_R	c_{50}	n	Classified
Mouse I	-0.6	-0.6	7.1	6.3	6	1.6	79%
Mouse II	-1.6	-0.5	9.5	6.9	25	0.8	76%
Mouse III	-0.9	-1.1	3.4	5.0	4	1.6	75%

Table 1. Fit parameters and fit quality for the three data sets illustrated in Figure 3. The first six columns are the parameters of the model. The seventh column is the percentage of trials that was correctly classified by the model.

Cross-validation indicated that for those data sets there would be no loss in fit quality if one imposed $s_L = s_R = s$, thus removing one free parameter. In those fits the bias parameters b_L and b_R changed by <0.3 and the values for s for the 3 mice were 6.6, 8.4, and 4.0, intermediate between the values found for s_L and s_R.

The model was fit by maximum likelihood estimation, using either MATLAB's inbuilt *fmincon* function or the *GLMNET* package (Qian,et al. 2013). The parameters c_{50} and n in Equation 1 were constrained to the ranges 0.1-80%, and 0-3.

In a logistic model, there is no established method to quantify fit quality. The natural approach would be to compare alternative models, which is not our goal here. As an alternative, one can simply calculate how well the model classifies the choice of each trial by taking the model's "choice" in each trial to be the one for which it predicts the maximum probability. By this measure, the model did well, correctly predicting >75% of the choices (Table 1).

When measuring the effects of inactivation (Figure 4 and Supplementary Figure 6), we fitted the different inactivation conditions independently, while imposing that the parameters of Equation 1, c_{50} and n, were constant across conditions. This allowed us to capture the effects of inactivation with changes in the 4 parameters of Equation 2.

Cortical inactivation

Inactivation experiments were performed with transgenic mice expressing ChR2 in Pvalb-positive inhibitory interneurons, obtained by crossing a $Pvalb^{tm1(cre)Arbr}$ driver (Jax #008069) with an Ai32 reporter (Jax #012569). Mice were prepared with a clear skull cap similar to that of Guo et al. (2014b) but with UV-curing optical adhesive (Norland Optical Adhesives #81, Norland Products Inc., Cranbury, NJ; from ThorLabs) instead of clear dental acrylic, and metal head -plate for head-fixation. In brief, the implantation surgery proceeded as follows. The dorsal surface of the skull was cleared of skin and periosteum and prepared with a brief application of green activator (Super-Bond C&B, Sun Medical Co, Ltd, Japan). A thin layer of cyanoacrylate was applied to the skull and allowed to dry. Two to four thin layers of UV-curing optical glue were applied to the skull and cured (~10 s per layer) until the exposed skull was covered (thin layers were used to prevent excessive heat production). Super-Bond polymer was applied around the edges to join the skin and the clear skull cap and enhance stability. A head-plate was attached to the skull over the interparietal bone with Super-Bond polymer.

Light for inactivation was produced by a 473 nm diode laser (LuxX diode laser, Photon Lines Ltd) coupled to a fiber and collimated to a circle of approximately 0.3 mm diameter on the skull. Total laser power at the surface of the skull was ~1.5 mW. The laser was mounted on a manipulandum, which was manually positioned at stereotaxic coordinates for the cortical regions, defined relative to Bregma: 3.3-3.7 mm posterior, 2.1 mm lateral for visual cortex; 0.8 mm posterior, 2.5 mm lateral for somatosensory cortex. Light was delivered as a 40 Hz sinusoid beginning 33.2±5.5ms (mean ± standard deviation) before the visual stimulus onset and lasting until the mouse made a response. The task was the 2AUC detection variant, but responses could be made immediately upon stimulus onset. During individual sessions, inactivation was performed on approximately 30% of trials, randomly selected. One session out of 34 was excluded because performance on trials without laser inactivation was poor (max percent correct <50% for highest contrast stimuli on one side).

Optogenetic dopamine stimulation

For optogenetic dopamine stimulation we used DAT-Cre mice that were heterozygous for Cre recombinase under the control of DAT gene (B6.SJLSlc6a3tm1.1(cre)Bkmn/J, Jackson Laboratory) backcrossed with C57/BL6J mice. We injected 1 µL of diluted virus (AAV5.EF1a.DIO.hChr2(H134R)-eYFP.WPRE, 2.8x10^{12} unit/ml) into VTA and SNc (injection coordinates, from Bregma: AP = -3 mm, lateral: 0.5 mm and dorsal-ventral: 4.4 mm). An optic fiber was implanted over the same stereotaxic coordinate but with the fiber tips 0.5 mm above the virus injection site. The fiber and the head -plate were secured with dental cement. We waited 3 weeks for virus expression before starting behavioral training. These mice had free access to food and water in their home cages and were trained in the 2AFC version of the task. In each trial, upon making a correct choice, animals received a short train of laser stimulation (473 nm, 12 pulses, pulse duration: 10 ms, inter pulse interval: 40 ms, laser power: 10-15 mW, measured at the tip of the fiber that was implanted in the brain) and a simultaneous click sound.

To quantify the specificity of ChR2 expression in dopamine neurons, animals were anesthetized (with sodium Pentobarbital) and perfused with 1X PBS followed by 4% formaldehyde in PBS. The brains were post-fixed in the same solution overnight and then kept in PBS containing 30% sucrose until settling. 50 µm coronal sections were collected and washed in PBS. Localization of fiber optic, DA cell bodies as well as ChR2-EYFP was confirmed using immunohistochemical methods (Tsai et al., 2009). Sections were immunostained with antibodies to TH (New Market Scientific, catalog No. 22941) and EYFP (Abcam, catalog No. AB6556) and secondary antibodies labeled with Alexa Fluor 488 and 594, respectively (Life Tech, catalog Nos. A11034 and A11032). We quantified infection efficiency and specificity by counting cells (1,460 neurons) from 121 confocal images collected from 11 animals.

Contrast discrimination task

This task is based on the 2AUC task above, but gratings could be presented on both sides of the screen simultaneously, and the mice were rewarded for choosing (i.e. centering) the grating with the highest contrast, or rewarded 50% of the time if grating contrasts were equal. As in the 2AUC task, no response after 1.5 seconds was registered as a no-go response and rewarded only if no stimulus was present.

Discriminations are introduced incrementally starting with easy discriminations and ending with equal contrasts on both sides. Adding harder discriminations was done at the discretion of the experimenter, typically on the very next session; no quantitative criteria were used. All mice that we attempted to train on this version learned it within a few days, starting from the 2AUC detection task.

References

Brainard, D.H. (1997). The Psychophysics Toolbox. Spatial Vision 10, 433-436.;

Chen, T.W., Wardill, T.J., Sun, Y., Pulver, S.R., Renninger, S.L., Baohan, A., Schreiter, E.R., Kerr, R.A., Orger, M.B., Jayaraman, V., et al. (2013). Ultrasensitive fluorescent proteins for imaging neuronal activity. Nature 499, 295-300.

Guizar-Sicairos, M., Thurman, S.T., and Fienup, J.R. (2008). Efficient subpixel image registration algorithms. Opt Lett 33, 156-158.

Pelli, D.G. (1997). The VideoToolbox software for visual psychophysics: Transforming numbers into movies. Spatial Vision 10, 437-442.

Pologruto, T.A., Sabatini, B.L., and Svoboda, K. (2003). ScanImage: flexible software for operating laser scanning microscopes. Biomed Eng Online 2, 13.

Qian, J., Hastie, T., Friedman, J., Tibshirani, R., and Simon, N. (2013) Glmnet for Matlab.

Remtulla, S., and Hallett, P.E. (1985). A schematic eye for the mouse, and comparisons with the rat. Vision Res 25, 21-31.
