# Supplementary figures and images for: High-Yield Methods for Accurate Two-Alternative Visual Psychophysics in Head-Fixed Mice
Source: Cell Rep. 2017 Sep 5;20(10):2513–24. doi: 10.1016/j.celrep.2017.08.047 (PMC5603732; doi:10.1016/j.celrep.2017.08.047)

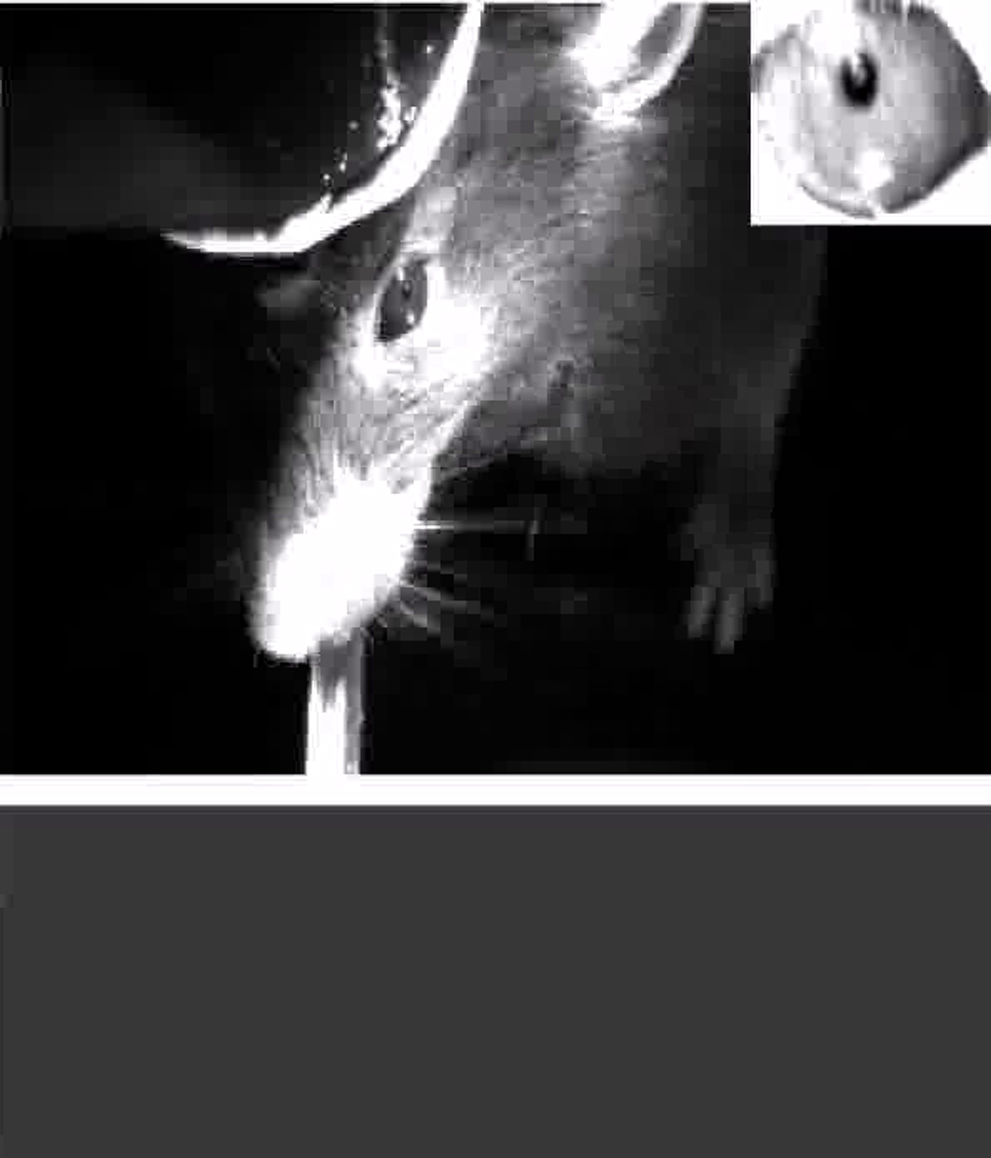

Supplement: Movie S1. A Mouse Performing the Two-Alternative Unforced-Choice Contrast Discrimination Task — The inset to the main panel shows the image taken by the eye camera. The panel below the main panel illustrates the images that appear on the three screens around the animal. [file mmc2.jpg]
