# Supplementary material for: High-Yield Methods for Accurate Two-Alternative Visual Psychophysics in Head-Fixed Mice
Source: Cell Rep. 2017 Sep 5;20(10):2513–24. doi: 10.1016/j.celrep.2017.08.047 (PMC5603732; doi:10.1016/j.celrep.2017.08.047)
Supplement: Document S2. Article plus Supplemental Information [file mmc3.pdf]

Cell Reports

Resource

High-Yield Methods for Accurate Two-Alternative Visual Psychophysics in Head-Fixed Mice

Graphical Abstract

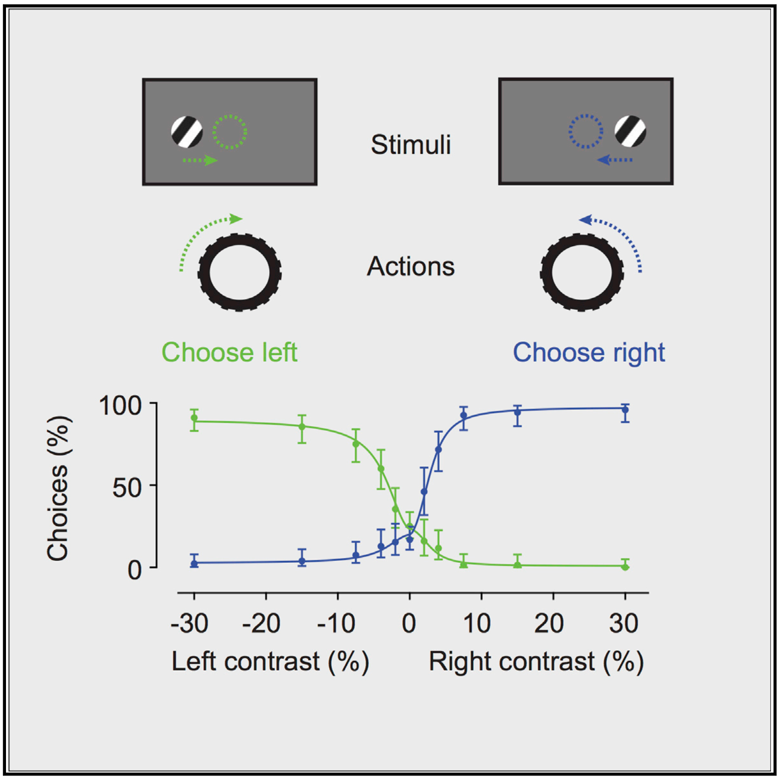

Highlights

- A platform to probe visual discrimination and its neural basis in head-fixed mice
- Extension of classic two-alternative choice design to unforced choices
- Probabilistic observer model for mouse decisions and effects of inactivation
- Optogenetic stimulation of dopaminergic neurons can replace water control

Authors

Christopher P. Burgess, Armin Lak, Nicholas A. Steinmetz, Peter Zatka-Haas, ..., Kenneth D. Harris, Matteo Carandini

Correspondence

m.carandini@ucl.ac.uk

In Brief

Burgess et al. introduce methods to probe visual discrimination and its neural basis in head-fixed mice. Mice turn a steering wheel to make two alternative choice, and their behavior matches a simple probabilistic observer. The task engages and requires the visual cortex. Optogenetic stimulation of dopaminergic neurons can replace water control.

Burgess et al., 2017, Cell Reports *20*, 2513–2524
September 5, 2017 © 2017 The Author(s).
http://dx.doi.org/10.1016/j.celrep.2017.08.047

Cell Reports
Resource

OPEN ACCESS
CellPress

High-Yield Methods for Accurate Two-Alternative Visual Psychophysics in Head-Fixed Mice

Christopher P. Burgess,[1,6,7] Armin Lak,[1,6] Nicholas A. Steinmetz,[2,6] Peter Zatka-Haas,[2,3,6] Charu Bai Reddy,[1] Elina A.K. Jacobs,[2] Jennifer F. Linden,[5] Joseph J. Paton,[4] Adam Ranson,[1,8] Sylvia Schröder,[1] Sofia Soares,[4] Miles J. Wells,[2] Lauren E. Wool,[2] Kenneth D. Harris,[2] and Matteo Carandini[1,9,*]
[1]UCL Institute of Ophthalmology, University College London, London WC1E 6BT, UK
[2]UCL Institute of Neurology, University College London, London WC1E 6BT, UK
[3]Centre for Mathematics and Physics in the Life Sciences and Experimental Biology (CoMPLEX), University College London, London, UK
[4]Champalimaud Centre for the Unknown, Lisbon, Portugal
[5]UCL Ear Institute, University College London, London WC1X 8EE, UK
[6]These authors contributed equally
[7]Present address: Google DeepMind, 5 New Street Square, London EC4A 3TW, UK
[8]Present address: Neurosciences & Mental Health Research Institute, Cardiff University School of Medicine, Cardiff CF24 4HQ, UK
[9]Lead Contact
*Correspondence: m.carandini@ucl.ac.uk
http://dx.doi.org/10.1016/j.celrep.2017.08.047

SUMMARY

Research in neuroscience increasingly relies on the mouse, a mammalian species that affords unparalleled genetic tractability and brain atlases. Here, we introduce high-yield methods for probing mouse visual decisions. Mice are head-fixed, facilitating repeatable visual stimulation, eye tracking, and brain access. They turn a steering wheel to make two alternative choices, forced or unforced. Learning is rapid thanks to intuitive coupling of stimuli to wheel position. The mouse decisions deliver high-quality psychometric curves for detection and discrimination and conform to the predictions of a simple probabilistic observer model. The task is readily paired with two-photon imaging of cortical activity. Optogenetic inactivation reveals that the task requires mice to use their visual cortex. Mice are motivated to perform the task by fluid reward or optogenetic stimulation of dopamine neurons. This stimulation elicits a larger number of trials and faster learning. These methods provide a platform to accurately probe mouse vision and its neural basis.

INTRODUCTION

Mice are increasingly used in research to understand the mammalian brain. The ease of husbandry, breeding, and handling has long been recognized, with the establishment of inbred lines to control for genetic variation (Beck et al., 2000). Today, the mouse offers an unrivaled arsenal of tools to the neuroscientist, from atlases of gene expression and connectivity (Lein et al., 2007; Oh et al., 2014; Zingg et al., 2014) to a plethora of genetic tools and transgenic lines (Harris et al., 2014; Heintz and Gerfen; Huang and Zeng, 2013; Madisen et al., 2015, 2012). Its lissencephalic cortex also makes it ideally accessible to imaging studies.

Mice are an excellent species for studying perception and cognition. They quickly learn to perform tasks based on touch (Guo et al., 2014a), olfaction (Liu et al., 2014; Resulaj and Rinberg, 2015), hearing (Hangya et al., 2015; Jaramillo and Zador, 2014; Pinto and Dan, 2015; Sanders and Kepecs, 2012), or vision (Andermann et al., 2010; Busse et al., 2011). Some of these tasks have been extended to probe not only perception but also cognition (Bussey et al., 2012; Nithianantharajah et al., 2015).

Contrary to past preconceptions, mice make major use of vision (Carandini and Churchland, 2013; Huberman and Niell, 2011). Their visual cortex comprises at least 12 retinotopic areas (Garrett et al., 2014; Glickfeld et al., 2014; Wang and Burkhalter, 2007). The division of labor across these areas and other general principles of visual function are likely to be conserved across species (Wang et al., 2011) and may be fruitfully investigated in the mouse.

Studying the neural activity underlying visually driven behavior, however, requires careful psychophysical means that constrain task design (Carandini and Churchland, 2013). An ideal task should (1) allow continuous control of visual stimulation and accurate measurement of eye position; (2) be easily paired with brain recordings or manipulations; (3) require a behavioral response that does not confound the neural activity related to sensory processing and decision-making; (4) be robust to changes in the observer's tendency to respond; (5) be learned quickly and reliably by most subjects; (6) yield many trials per stimulus and session, to deliver precise psychometric curves relating task performance to visibility; (7) yield close to 100% accuracy on easy trials, to distinguish errors due to the limits of vision from those due to other sources (disengagement, confusion about the task rules, motor errors); and (8) be flexible, so that its design can be made more complex if needed. Finally, it would be ideal if the task could (9) involve only positive reward, without requiring controlled access to food or water.

These fundamental requirements are not met by existing techniques for mouse visual psychophysics.

The first two requirements—control of visual stimulation and the ability to record and manipulate neuron activity—strongly argue in favor of head fixation, ruling out techniques based on

This is an open access article under the CC BY license (http://creativecommons.org/licenses/by/4.0/).

swimming (Prusky et al., 2000) or nose poking (Busse et al., 2011; Bussey et al., 2012; Long et al., 2015; Nithianantharajah et al., 2015). Some approaches available to study vision are compatible with head fixation, but they probe innate subcortical behaviors such as the optokinetic reflex (Cahill and Nathans, 2008).

The third requirement—a behavioral response that does not confound sensory activity—rules out behavioral reports such as locomotion or navigation (Harvey et al., 2012; Poort et al., 2015; Wekselblatt et al., 2016). These elicit strong responses in mouse visual cortex (Niell and Stryker, 2010), confounding sensory or decision-related signals.

The fourth requirement—robustness to the observer's tendency to respond—argues for having the observer choose between concurrent stimuli (Carandini and Churchland, 2013), like in a two-alternative choice design. This rules out go/no-go designs such as those in which the mouse reports the presence of a visual stimulus by licking a single spout (Andermann et al., 2010; Glickfeld et al., 2013; Goard et al., 2016; Lee et al., 2012). Promising methods for two-alternative choices in head-fixed mice are available to probe audition, somatosensation, and olfaction (Guo et al., 2014a; Resulaj and Rinberg, 2015; Sanders and Kepecs, 2012), but not to study vision.

Finally, all existing techniques make use of implicit punishment: the reward redresses an unpleasant circumstance, such as swimming in deep water (Prusky et al., 2000) or having limited access to drinking water (Andermann et al., 2010; Busse et al., 2011; Bussey et al., 2012; Glickfeld et al., 2013; Lee et al., 2012; Long et al., 2015; Nithianantharajah et al., 2015).

We developed a task that meets the above requirements with a behavioral response based on turning a steering wheel left or right to make a two-alternative choice between visual stimuli. The choice of a steering wheel was inspired by tasks that probe hearing and olfaction, which involve a conveyor belt or a spherical ball (Resulaj and Rinberg, 2015; Sanders and Kepecs, 2012). To train the mice in this task, we introduced an intuitive coupling of the steering wheel to the position of the visual stimuli. Mice learn this task within weeks, they perform it proficiently, and their decisions conform to the predictions of a simple probabilistic observer model. The task can be paired with two-photon imaging, activates visual cortex, requires visual cortex, and can be flexibly extended to probe unforced choices, both for stimulus detection and discrimination.

Mice performed the task when rewarded with water or with stimulation of midbrain dopamine neurons. Optogenetic stimulation of these neurons is known to elicit coarse behavioral outcomes (Tsai et al., 2009) or repetitive actions (Kim et al., 2012). Here, we show that it acts as a powerful reward in precise actions driven by perceptual decisions.

RESULTS

We first introduce a basic version of the task: two-alternative forced-choice (2AFC) detection with a water reward. We then show that this task is compatible with cortical recordings, that it can be extended to unforced choices, that it elicits decisions that conform to a probabilistic model, and that these decisions require visual cortex. Finally, we illustrate a variation in which the reward is optogenetic stimulation of dopamine neurons and one that requires discrimination between two stimuli.

The Basic Task: Two-Alternative Forced Choice

The head-fixed mouse is trained to select one of two choices by turning a steering wheel placed under its front paws (Figure 1A). It was highly advantageous to couple wheel movements to the visual stimuli, so that turning the wheel would accordingly move the stimuli (Figure 1A, right; Movie S1). The mouse indicates its choice by bringing one stimulus to the center of the visual field.

The typical sequence of trial events was as follows (Figure 1B). First, the mouse kept the wheel still (quiescent period) to initiate the trial. Second, an onset tone signaled the appearance of the stimuli, and, during an "open loop" period, wheel movements were ignored. Mice generally continued to hold the wheel still in this period (and this could be enforced through training if desired). Third, a go tone was played (e.g., a 12-kHz pure tone lasting 100 ms), after which point the wheel turns resulted in movements of the visual stimuli ("closed loop"; Figure 1B). If the mouse turned the wheel such that the stimulus reached the center of the screen, the animal received water (1–3 μL). If instead the mouse moved the stimulus by the same distance in the opposite direction, this incorrect decision was penalized with a timeout (typically, 2 s) signaled by auditory noise. In either case, the grating remained locked in its response position for 1 s and then disappeared.

Depending on the experimental requirements, in many mice we slightly varied this sequence of events. For instance, if an experiment could tolerate motor actions prior to visual stimulation, we omitted the quiescent period. Similarly, we introduced the open loop period only if we wanted to delay motor actions or visual motion after stimulus presentation. Likewise, we played the onset and go tones only if we did not mind evoking auditory activity, and we shortened the inter-trial interval to maximize trial number. Our analyses here do not distinguish among these variations because other key factors covaried with them: experimenter, time of day, experimental rig, home cage, etc. A proper comparison would have to correct for these factors.

Training for a typical mouse proceeded in two main stages (Figures 1C–1E). We started mice on easy (high) contrasts, until they learned the association between turning the wheel, moving the stimulus, and receiving reward. This association was necessary for learning: in a few attempts in which we did not use the closed loop period, mice did not learn the task. When mice performed above chance for a day or two (which typically occurred by the first week), we introduced lower contrasts. A typical mouse (Figure 1C) reached 56% performance (with 95% confidence) on high contrast stimuli on day 5, after ~2,300 trials (Figures 1D and 1E, blue), after which we introduced lower contrast stimuli. Psychometric functions of stimulus contrast and position were obtained by week 3 (Figure 1C). By week 4, this mouse had mastered the task.

These results were typical of our population (n = 98 mice; Figures 1D–1G). Most mice were above chance before ~1,000 trials (Figure 1D), corresponding to a few days of training (Figure 1E). Mice then typically approached steady performance after 7,000–10,000 trials (Figure 1D), i.e., in 20–30 days (Figure 1E).

Very few mice (6/98) failed to learn the rudiments of the task (performance significantly above 50%) by trial 5,000 or after 2 weeks (Figures 1F and 1G). Most animals surpassed 80% performance, but a sizeable fraction (38/98) also reached 90% performance (Figures 1F and 1G). This method worked even though different cohorts were trained by different experimenters using different subjective criteria for advancing a mouse from one stage of training to the next.

Once they mastered the task, mice typically produced stereotyped movements, with initial wheel deflections usually matching the final responses (Figure S1). The movements elicited by high contrast stimuli typically had shorter latency and higher peak velocity. Movements otherwise showed little variability across trials. If desired, we could then modify the task by removing the coupling between wheel position and stimulus position, so that the stimulus would stay fixed in its position (Figure S2), or disappear as soon as the movement started.

Some mice moved their eyes following stimulus onset or showed changes in pupil diameter associated with trial structure (Figure S3). These eye movements and pupil dilations, however, varied across trials and across mice, highlighting the importance of imaging the eye in all experiments.

Simultaneous Recordings in the Visual Cortex

To pair this task with measurements of brain activity, we performed two-photon imaging in primary visual cortex (V1) (Figure 2). We expressed GCaMP6m in V1 neurons via virus injection. In this task version, mice had to hold the wheel still for a 2- to 3-s quiescence period, and the open-loop period lasted 1 s (Figure 1B). During this period, we could image neural responses without the stimulus moving. We chose a field of view with neurons whose receptive fields overlapped with the contralateral stimulus (Figure 2B).

As expected, most visually responsive neurons showed robust responses to contralateral stimuli and no responses to ipsilateral stimuli (Figures 2C and 2D). The amplitudes of these responses grew with the contrast c of contralateral gratings (Figure 2D). We fit these responses with the commonly used function

$$f(c) = \frac{c^n}{c_{50}^n + c^n}, \tag{1}$$

where c_{50} and n are free parameters (Albrecht and Hamilton, 1982; Sclar et al., 1990). These results were robust across

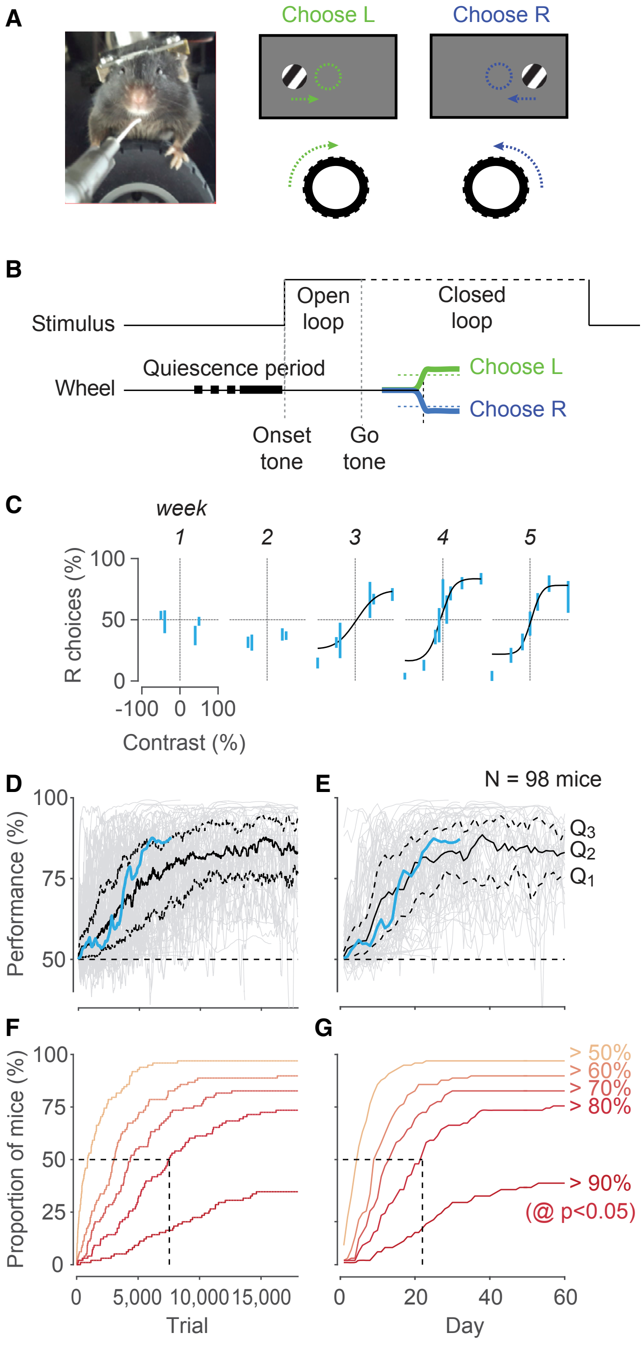

Figure 1. The 2AFC Version of the Stimulus Detection Task

(A) Left: a head-fixed mouse with forepaws on a steering wheel used to make choices. Right: at onset, the grating is either on the left or on the right, and the mouse turns the wheel (arrows) to move the grating to the center (dashed circles).

(B) Time course of the basic task. Mice start the trial by holding the wheel still (quiescence). An onset tone may be played. The stimulus appears. Its position is initially fixed (open loop). After an optional go tone, stimuli become coupled with wheel position (closed loop). Choices are registered when the stimulus reaches the center of the screen (correct) or an equal distance in the opposite direction (incorrect).

(C) Psychometric data obtained in the first 5 weeks for an example mouse. Bars show the percentage of times the mouse chose the right stimulus (95% binomial confidence intervals), as a function of stimulus contrast. By convention, we plot contrast of left stimuli as negative. Curves are fits with a psychometric curve.

(D) Learning rates for a population of 98 mice. Performance is assessed on easy stimuli ($\geq$40% contrast), as a function of number of trials. Blue trace highlights the example mouse in (C). Gray traces indicate performance by individual mice. Black traces indicate the 3 quartiles: the median (Q2) and the 25th and 75th percentiles (Q1 and Q3). The approximate chance level is 50% (dashed line).

(E) Same as in (D), as a function of training days.

(F) Cumulative probability of proportion of mice surpassing a given performance level as a function of trial number.

(G) Same as in (F), as a function of training days.

See also Figures S1–S3.

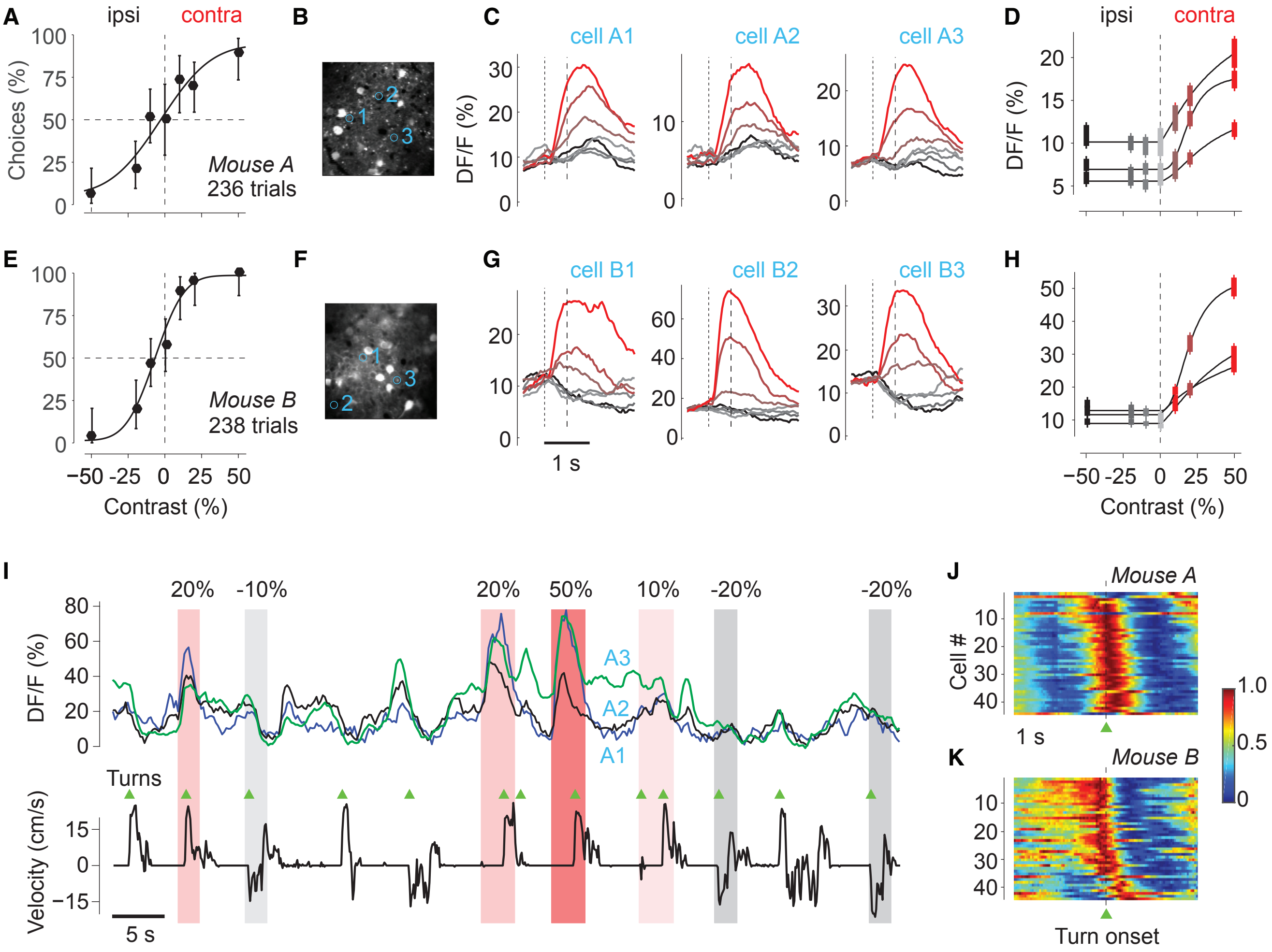

Figure 2. Imaging in V1 during the Task

(A) Psychometric curve for an example mouse, measured during two-photon imaging in area V1. Error bars are 95% binomial confidence intervals.

(B) Imaging field of view, with 3 cells circled and numbered.

(C) Mean calcium activity averaged around the onset of the grating stimulus, grouped by stimulus condition (see color codes in next panel) for the 3 cells. Dotted line marks stimulus onset (preceded by a 2- to 3-s quiescence period). Dashed line marks the beginning of the closed-loop period, when the stimulus becomes movable. Data were taken from 181 trials (22–30 per condition).

(D) Response amplitudes of each cell as a function of stimulus contrast. Positive and negative contrast denotes stimuli in the contralateral and ipsilateral visual fields. Amplitude is mean response at 1 s after grating onset. Curves indicates fits of the function $p + qf(c)$, with $f(c)$ defined in Equation 1. Error bars indicate SEM.

(E–H) Same as (A)–(D), for a different mouse. Data were taken from 210 trials (24–43 per condition).

(I) Example traces from the cells in (B)–(D) in the presence of stimuli of different contrasts (shaded areas) and in relation to wheel velocity (bottom trace). There are strong responses to the visual stimuli but also small responses synchronized with turn onsets (triangles). Onsets and offsets of wheel turns were identified by applying a dynamic threshold based on a Schmitt trigger to the wheel velocity traces.

(J) Time course of movement-related activity in the absence of visual stimuli in 45 neurons from each of the 2 mice. Neurons were selected based on the quality of segmentation. We triggered calcium activity on wheel turn onsets, averaged across events, and normalized the results for each neuron (rows) to range from 0 to 1. Neurons were sorted by the amplitude 1 s before turn offset.

(K) Same as in (J) for mouse B.

mice (e.g., Figures 2E and 2H) and demonstrate that the task can be readily paired with recording techniques requiring high stability and evoke contrast-dependent activity in cortex.

V1 activity also included small fluctuations that tended to precede wheel movements (Figures 2I–2K). Large responses to contralateral stimuli (Figure 2I) were not the sole activity observed. Even in the absence of visual stimuli, activity built up before wheel turns, perhaps reflecting increased alertness (Burgess, 2016), and decayed following the onset of wheel turns (Figures 2J and 2K). This buildup of activity, nonetheless, was dwarfed by visual responses. For instance, for the 6 example cells (Figures 2B–2D and 2F–2H), the build-up activity was 7.5 ± 0.8 times smaller (mean ± SEM) than the responses to 50% contrast contralateral stimuli.

OPEN ACCESS
CellPress

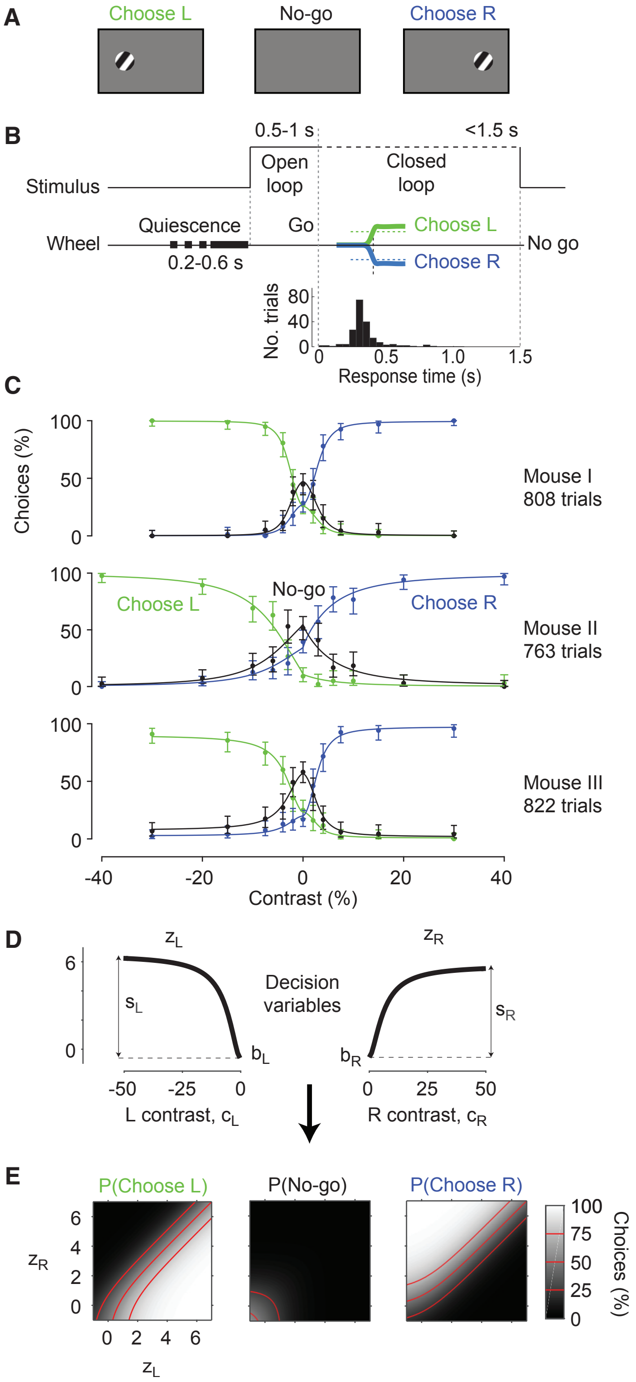

Figure 3. Elaboration of the Stimulus Detection Task in a 2AUC Version

(A) In the 2AUC task, the mouse learns to choose left when the stimulus is on the left, choose right when the stimulus is on the right, and hold still (no-go) if the stimulus is absent.

(B) Time course of the 2AUC task. At the go cue, the mouse has 1.5 s to move the wheel. Holding the wheel still for this period counts as a no-go choice. Histogram shows a typical distribution of response times in a session (time from go tone to response).

(C) Choices as a function of stimulus contrast and position, for three sessions in 3 mice (rows). For each mouse, the data show the proportion of left (green), right (blue), and no-go choices (black) as a function of stimulus contrast. Negative contrast denotes stimuli appearing on the left side. Curves show fits of the probabilistic observer model. Error bars are 95% binomial confidence intervals.

(D) The decision variables in the probabilistic observer model, with parameters obtained from mouse 1. The decision variables z_L and z_R grow with contrast presented on the left or on the right. Each function is defined by 2 parameters: bias, b, and sensitivity, s (Equations 1 and 2).

(E) The probability of left, no-go, and right choices depends on the 2 decision variables. This dependence is parameter-free (Equation 3).

See also Figure S4.

Two-Alternative Unforced Choice

Next, we extended the two-alternative tasks by adding a "no-go" response option when there was no stimulus. The result is the two-alternative unforced-choice (2AUC) task, which allows one to measure sensitivity and bias separately for the two stimulus locations. This is particularly useful following unilateral manipulations in task context or brain activity (Sridharan et al., 2014).

Mice were readily able to learn the 2AUC version of the task (Figures 3A–3C). Training started on the two-alternative forced-choice task, then we constrained the response window to 1.5 s and added the no-go condition: when the stimulus was absent (zero contrast), mice earned the reward by not turning the wheel (no-go; Figure 3A) for 1.5 s (Figure 3B). Mice typically learned this new response contingency in 5 or 6 sessions. Their reaction times for left (L) or right (R) responses were much faster than the 1.5-s response window (Figures 3B and S4), indicating that issuing a no-go response was distinct from simply being slow to respond. Mice correctly made most no-go choices at zero contrast, and made progressively fewer of them as stimulus contrast increased (Figure 3C).

This 2AUC version of the task thus yields 3 psychometric curves indicating probability of choosing L, of choosing R, and of choosing no-go (Figure 3C). Although this representation is redundant (the probabilities must sum to 1, so one curve is fully constrained by the other two), it helps to view all 3 to understand the data and to develop a simple observer model to interpret and fit the data.

Probabilistic Observer Model

The decisions made by the mice closely matched the predictions of a simple probabilistic model. We present here the model for the 2AUC version of the task, which can be easily reduced to the 2AFC version.

In the model, choices depend on two decision variables, one for choosing L and one for choosing R, each depending on the contrast c_L and c_R on the left and right:

$$\begin{aligned} z_L &= b_L + s_L f(c_L) \\ z_R &= b_R + s_R f(c_R) \end{aligned} . \qquad (2)$$

Here, $f(c)$ is the function in Equation 1, b_L and b_R represent bias toward choosing L or R relative to no-go, and s_L and s_R measure the weight assigned to visual evidence on the left or right (Figure 3D).

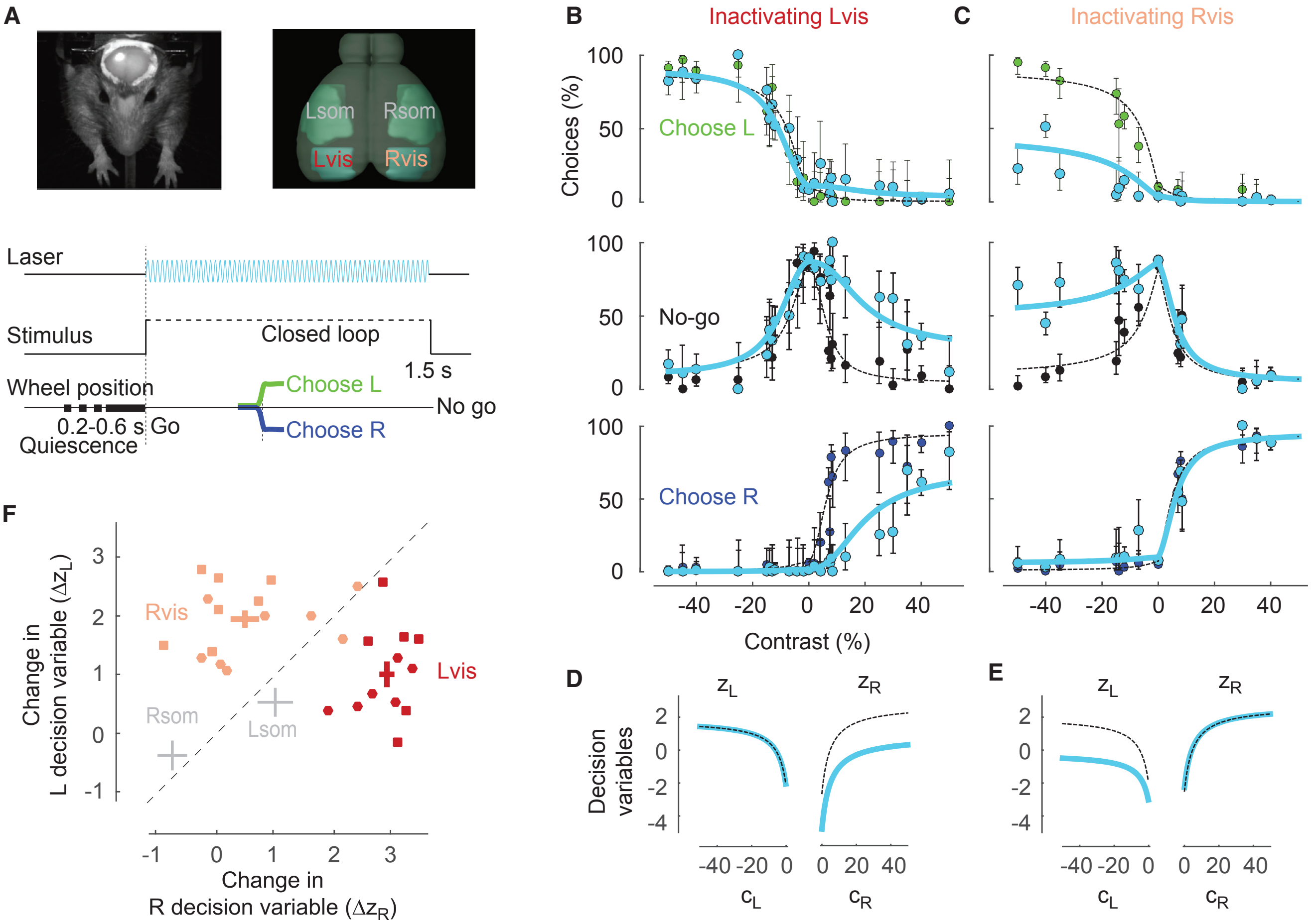

Figure 4. Effects of Optogenetic Inactivation of Visual Cortex

(A) Methods of optogenetic inactivation during the 2AUC task. Top left: image of a mouse with the clear skull preparation, with laser spot on right hemisphere. Top right: illustration of the regions inactivated: left and right visual cortex (Lvis and Rvis) and, as a control, left and right somatosensory cortex (Lsom and Rsom). Inactivation of these regions was performed in different sessions. Bottom: time course of the task. In ~33% of trials, stimuli were accompanied by laser illumination.

(B) Effects of inactivation of left visual cortex. Proportion of left, no-go, and right choices as a function of stimulus contrast, under control conditions (green, black, and blue dots) and during optogenetic inactivation (cyan dots). Curves indicate fits of the probabilistic model under control conditions (dashed) and during optogenetic inactivation (cyan). Error bars show 95% binomial confidence intervals. Data were obtained in 6 sessions from 1 mouse.

(C) Same as in (B), for inactivation of right visual cortex from the same mouse. Data were obtained in 7 sessions.

(D) Decision variables obtained by the model fits in (B) as a function of contrast on the left and right in control condition (dashed) or during inactivation of left visual cortex (cyan).

(E) Same as (D), for inactivation of right visual cortex.

(F) Summary of the effects of optogenetic inactivation in the 4 regions outlined in (A). Effects are measured by the decrease in the left and right decision variables, z_L or z_R, at 50% contrast. Dots indicate individual sessions from 2 mice (squares for the mouse in B–E, circles for another mouse) with inactivation of left visual cortex (red) or right visual cortex (pink). Crosses summarize the effects of inactivation in visual cortex (red and pink), and in somatosensory cortex (gray). The length of the crosses indicates ± SEM in the 2 dimensions.

See also Figures S5 and S6.

The decision variables, in turn, determine the probabilities p_L, p_R, and p_0 of choosing L, R, or no-go (Figure 3E), and specifically the log odds of choosing L or R versus choosing no-go:

$$\begin{aligned} \log(p_L/p_0) &= z_L \\ \log(p_R/p_0) &= z_R \end{aligned}. \tag{3}$$

With 6 free parameters, the model provided good fits to the 22 response probabilities, explaining over 75% of individual choices (curves in Figure 3C). Cross-validation indicated that for these 3 datasets there would be no loss in fit quality if one imposed $s_L = s_R$, thus removing one free parameter. However, as we will see, these two parameters must be allowed to differ when evaluating the effects of unilateral inactivation.

Inactivation in the Visual Cortex

To assess whether visual cortex was required for task performance, we silenced it optogenetically during individual trials (Figure 4A). We used 2 transgenic mice expressing

channelrhodopsin-2 (ChR2) in *Pvalb*-positive inhibitory interneurons, implanted with clear skull caps (Lien and Scanziani, 2013; Guo et al., 2014b). We used a 473-nm laser to inactivate the left or right visual cortex (somatosensory cortex for control measurements) during visual stimulus presentation and wheel-turn responses. Electrophysiological measurements show that such inactivation was circumscribed to a radius of ~1 mm (Figure S5).

Inactivation of visual cortex strongly suppressed the mouse's ability to detect contralateral stimuli, but had little effect on the detection of ipsilateral stimuli (Figures 4B and 4C). To summarize these effects and compare them across experiments, we used the probabilistic model (Figures 4D–4F). In the example experiment, inactivating left visual cortex reduced only the decision variable for right stimuli (z_R; Figure 4D), and inactivating right visual cortex reduced only the decision variable for left stimuli (z_L; Figure 4E). Similar results were seen across experiments (Figure 4F): inactivating left visual cortex decreased z_R by 2.9 ± 0.1, significantly more than z_L (1.0 ± 0.2; paired t test, one-sided, $p < 10^{-5}$), and inactivating right visual cortex decreased z_L by 2.0 ± 0.2, significantly more than z_R (0.5 ± 0.2; $p < 10^{-4}$).

By comparison, in control experiments in which we inactivated the somatosensory cortex, we saw no such effects (Figures 4F and S6). Inactivating somatosensory cortex did not cause any significant change in decision variables (p = 0.17 and p = 0.25 for left and right somatosensory cortex; Figure S6). Indeed, the effect on the R decision variable was significantly weaker during inactivation of left somatosensory cortex than of left visual cortex (p = 0.00015, Wilcoxon rank-sum test). Similar effects were seen on the L decision variable following inactivation of right somatosensory versus visual cortex (p = 0.00012). We conclude that accurate performance on this task requires the visual cortex.

Rewarding with Optogenetic Dopamine Stimulation

The conventional method to reward mice for performing perceptual decisions involves delivering fluids under conditions of water control. It would be ideal, however, if one could deliver reward without any water or food control. We sought to achieve this goal by stimulating brain centers that mediate the effects of positive reinforcement. We provided phasic optogenetic stimulation of midbrain dopamine neurons. Phasic stimulation of these neurons is known to be sufficient for simple behavioral conditioning, such as place preference, lever pressing or nose poking (Kim et al., 2012; Olds and Milner, 1954; Tsai et al., 2009). However, it is not known whether trial-by-trial stimulation of these neurons can act as an efficient reinforcer for perceptual choices.

We injected a viral construct containing Cre-dependent ChR2 into ventral tegmental area (VTA) and substantia nigra pars compacta (SNc) of $DAT^{IREScre}$ mice, and implanted an optic fiber above VTA (Figure 5A). We confirmed specific expression of ChR2 in dopamine neurons using immunohistochemistry (Figure 5B). We identified dopamine neurons as those that stained for tyrosine hydroxylase (TH^+). 71% of these neurons also expressed ChR2. On the other hand, only 5% of neurons that expressed ChR2 failed to react to TH staining, indicating that expression was highly selective to dopamine neurons. ChR2 expression was consistent across animals and was stable for months after virus injection (n = 1,460 neurons in 11 mice; Figure 5C).

We then trained 3 naive mice in our 2AFC task by reinforcing correct choices with only optogenetic dopamine stimulation and an associated click sound. Mice were not given water reward, and had free access to water in their home cage.

Mice trained with optogenetic dopamine stimulation rapidly learned the task, greatly outperforming animals trained for a water reward, both in learning speed and in number of trials per session (Figures 5D–5G). After only a few days of training with dopamine stimulation, mice often performed over 900 trials per session (in more than 50% of sessions), with high accuracy (>75%, Figures 5D and 5E), resulting in high-quality psychometric curves (Figure 5F). On average, mice rewarded with dopamine stimulation performed almost twice as many trials per session as those rewarded with water (Figure 5G). To assess the stability of dopamine stimulation as a means of providing reward, in one mouse we continued these measurements for 10 weeks, during which the method remained robust.

The click sound at the onset of the optogenetic stimulation may be important for the success of these experiments for two reasons. First, when we attempted to train a mouse with optogenetic stimulation but no click sound, the animal did not learn the task. Second, it is known that sensory stimuli can be powerful secondary reinforcers (Herrnstein, 1964), and click sounds are particularly effective in "clicker training" (Pryor, 1999).

Stimulus Discrimination

A method for performing psychophysics should be flexible, so that it can be altered as needed. For instance, the basic tasks that we have described, whether 2AFC or 2AUC, involve detecting the position where a stimulus appears, either on the left or on the right. To study the mechanisms that combine information across hemispheres, however, it is useful to have the subject discriminate between stimuli that appear on both sides, as in contrast discrimination tasks commonly used with human observers (Boynton et al., 1999; Legge and Foley, 1980; Nachmias and Sansbury, 1974).

Mice that had already learned 2AUC contrast detection readily learned to perform contrast discrimination (Figure 6). In most trials, 2 stimuli appeared on the screen, and mice were rewarded with water for selecting the stimulus with higher contrast (Figure 6A). A no-go response was rewarded only when no grating was presented on either side. If contrasts were nonzero and equal, mice were rewarded randomly with 50% probability for left or right responses. Mice learned this task generalization, yielding high-quality psychometric curves (Figure 6B). When both gratings were present (a positive "pedestal contrast," Legge and Foley, 1980), mice correctly gave fewer no-go responses, while finding it harder to indicate the side with higher contrast (Figures 6B–6D). Their decisions conformed closely to the predictions of the probabilistic observer model (Figure 3). With a fixed setting of its 6 parameters the model provided satisfactory fits to the 32 response probabilities measured across 3 pedestal contrasts.

These results illustrate this task's suitability for bringing to the mouse methods that are traditional in human visual

OPEN
ACCESS
CellPress

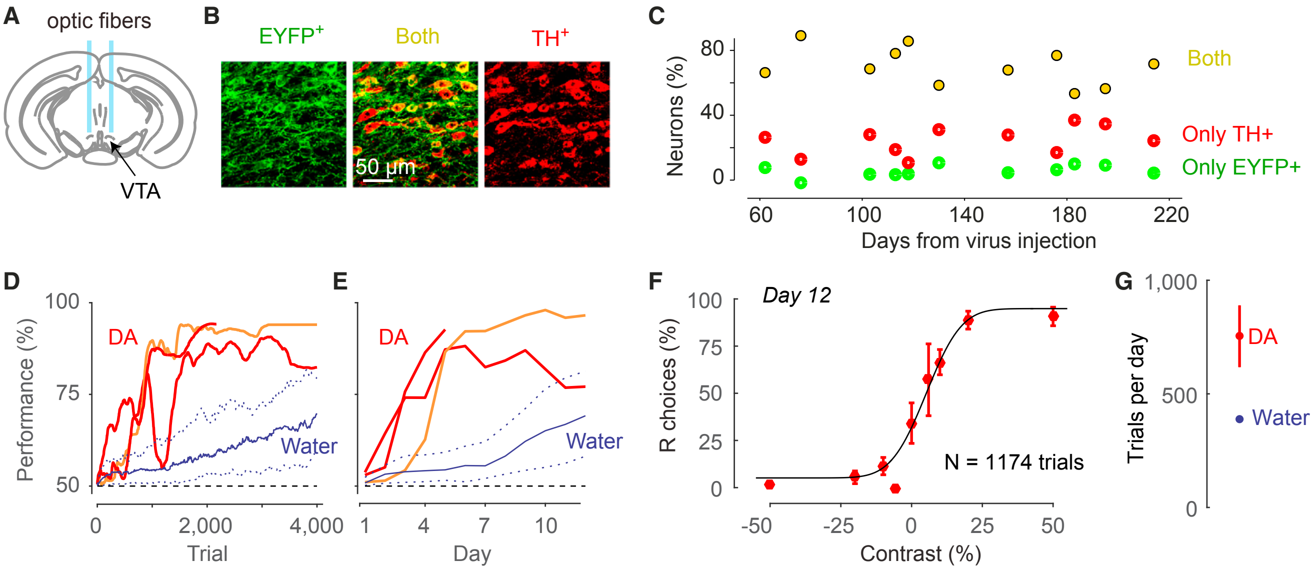

Figure 5. Using Optogenetic Phasic Dopamine Stimulation to Train Mice in the Task

(A) Schematic coronal section of the mouse brain (at the bregma, 3.1 mm) showing ventral tegmental area (VTA) and fiber optics implanted above VTA to elicit release of dopamine (DA).

(B) Confocal images showing expression of ChR2-EYFP (green) in TH^+ (DA) neurons (red) and overlay showing both (yellow). The bars quantify the specificity of expression, showing statistics of ChR2-EYFP and TH^+ expression in midbrain neurons (n = 1,460 neurons counted in 121 confocal images acquired from 11 mice).

(C) Stability of ChR2 expression in DAergic neurons (n = 11 mice).

(D) Rapid learning of the task in 3 mice receiving DA stimulation as a reward. Red and orange lines show rapid increase in the performance of naive mice that were solely trained with optogenetic DA stimulation. Blue curves show results for mice that trained with water reward (median and quartile ranges, replotted from Figure 1).

(E) Same as in (D), as a function of training day.

(F) Psychometric function obtained from example animal (orange line in C and D) on the 12th day of behavioral training. Error bars show 95% binomial confidence intervals.

(G) Mean trials per day of mice receiving DA stimulation (red) compared to water reward (blue). Error bars represent SEM (smaller than the dot for water reward).

psychophysics. These can be useful both to probe mouse vision and to relate perceptual decisions to neural activity.

DISCUSSION

We describe a flexible task for assessing visual decision-making in head-fixed mice. The steering wheel allows mice to accurately report one of two alternative stimuli, and the task is readily extended to allow a no-go response option. The task is learned quickly and reliably: most mice master it within a few weeks. The task yields a large number of trials per session, providing high-quality psychometric curves within individual sessions.

Mice are head-fixed, which facilitates not only brain recordings and manipulations but also careful control of visual stimulation and measurement of eye position. Mice sometimes moved their eyes during the same epochs as wheel turns, and these eye movements would correlate with neural activity. Tracking these behaviors and understanding their relationship with neural activity is an important control and an interesting direction for further research.

The decisions made by mice in this task follow the predictions of a simple probabilistic observer model. We formulated the model in terms of log odds (multinomial logistic regression), inspired by an earlier formulation based on signal detection theory (Sridharan et al., 2014). Both formulations are two-dimensional: responses depend on the combination of two decision variables. This is essential to capture the effects of unilateral inactivation, which would not be captured by models with a single decision variable (García-Pérez and Alcalá-Quintana, 2011, 2013; Kiani and Shadlen, 2009). Our formulation has two advantages over the earlier one (Sridharan et al., 2014). The first is technical: having a functional dependence on stimulus contrast minimizes free parameters. The second has broader import: by recasting the model as a logistic regression, it is easier to modify the analysis to include other predictors such as choice history (Abrahamyan et al., 2016; Bak et al., 2016; Busse et al., 2011; Licata et al., 2017) or neural activity (Nienborg and Macke, 2014). Including a neural signal as a predictor provides a means to assess whether that signal is informative of the animal's decisions.

We also demonstrated that transient optogenetic dopamine stimulation is sufficient for mice to learn a perceptual decision task. The combination of our task and dopamine stimulation may be useful for studying the effects of dopamine signals on perception and perceptual learning (Ding and Gold, 2013; Schultz et al., 1997). Our results show that dopamine stimulation is an attractive alternative to water reward, accelerating task acquisition and almost doubling trial counts. A large number of

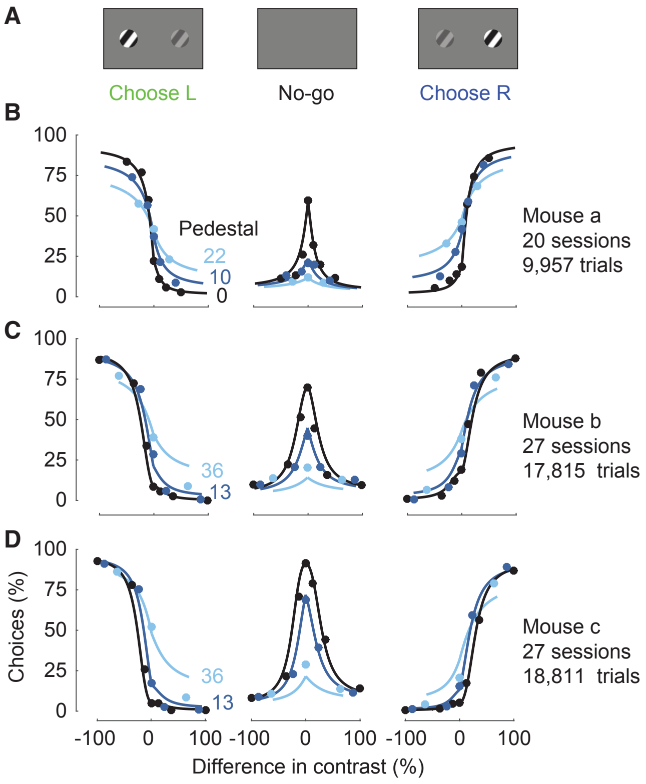

Figure 6. Extension of the 2AUC Task to the Study of Contrast Discrimination

(A) Stimulus conditions used in the discrimination task. Gratings are presented on both sides and the mouse is rewarded for choosing the side with the highest contrast, or opting for no-go if both contrasts are zero.

(B) Psychometric data from 1 mouse. Panels show left choices, no-go choices, and right choices, as a function of the difference between left and right contrast (c_R-c_L). Colors indicate the pedestal contrast, i.e., the minimum contrast present on the screen, min(c_L,c_R).

(C and D) Same as in (B) for 2 more mice.

trials are particularly useful when relating perceptual decisions to neural activity. Moreover, the method is arguably less disruptive of normal mouse behavior and physiology, as it does not constrain water intake.

As currently implemented, however, our optogenetic method also carries limitations. First, it requires the use of DAT-Cre mice, which may not be feasible if Cre needs to be expressed in other cells for other experimental purposes. Second, it requires implantation of optic fibers, which take up valuable space on the mouse head.

An advantage of our task is that it is highly flexible, allowing for many extensions of the same basic design. We have modified the task depending on requirements, for example introducing a cue informing mice when to respond, and a no-go response option to report stimulus absence. We exploited this no-go response in inactivation experiments, finding that inactivation of visual cortex diminished reports of contralateral stimuli but left ipsilateral reports unaffected. We also modified the task in a variant requiring contrast discrimination between two stimuli, generating high-quality psychometric functions that were modulated by contrast difference and by the pedestal contrast. We also found that, once trained, mice continue to perform if the stimulus position is fixed or is only transiently presented, which can be exploited to address concerns about stimulus movement being related to choice, or of presentation duration being controlled by the mouse.

We believe that the coupling of wheel movements to stimulus properties is a particularly useful learning aid, and is further generalizable. For example, the task can be extended beyond the detection or discrimination of visual contrast. In preliminary results (data not shown), we have trained mice to use the wheel to rotate a grating to a target orientation or to modulate repeated tones toward a target pitch.

Moreover, the continuous readout available from the steering wheel may provide further insight into the nature of behavior. We used the wheel to obtain discrete reports, but the continuous readout may afford more sensitive assays, probing factors such as motivation, confidence (Lak et al., 2014), response vigor, and vacillation (Resulaj et al., 2009). These considerations suggest additional extensions of the task to a fully interactive, flexible, and accurate platform for probing mouse vision and visuomotor behavior and establish their neural basis.

EXPERIMENTAL PROCEDURES

All experiments complied with the law governing animal research, i.e., the Animals (Scientific Procedures) Act 1986 Amendment Regulations 2012, in the United Kingdom. Procedures were approved by the local Animal Welfare Ethical Review Body and by the Home Office (license 70/8021). Detailed methods are described in the Supplemental Experimental Procedures.

To allow head-fixing, mice (male and female, aged 8–24 weeks) were first anesthetized and implanted with metal head-plates. After at least 4 recovery days, mice were acclimatized with head-fixing and then trained in a simplified version of the task involving only stimuli with high contrast and no timing requirements. As performance improved, lower contrasts and more stringent timings were introduced. Training criteria were qualitative and differed across experimenters and mice.

Most mice were trained using water as a reward. After the task, they received top-up fluids to achieve a minimum daily amount of 40 ml/kg/day. Body weight and potential signs of dehydration were monitored daily.

Stimuli were presented on 1 LCD monitor or on 3 monitors placed around the animal. Intensity values were linearized with a photodiode. In some experiments, we covered the monitors with plastic Fresnel lenses to make intensity spatially uniform. The response wheel was a Lego rubber tire, whose angle was measured using a rotary encoder. A detailed parts list is available at http://www.ucl.ac.uk/cortexlab/tools/wheel.

Stimuli were typically sinusoidal gratings in a Gaussian window, but the specifics of this stimulus generally differed by mice. To measure pupil position and dilation, we used a camera focused on one eye, illuminated by an infrared LED, and fitted a 2D ellipse to the pupil.

Imaging was performed in three 10- to 12-week-old C57BL/6J female mice. During the initial surgery, we performed a craniotomy centered on the right primary visual cortex and injected a GCaMP6m virus (AAV2/1-*syn*-GCaMP6m-WPRE). We sealed the craniotomy with coverslips and dental cement. We began calcium imaging 3 weeks after virus injection. Imaging was performed using a Sutter two-photon microscope controlled by ScanImage, with a Coherent Chameleon laser (1,000 nm) and Olympus 20× objective. We chose a field of view with good GCaMP expression and mapped the preferred stimulus position of the field of view, using this for the position of the task stimulus during behavior. We registered the raw calcium movies by aligning each frame to a reference frame and found neurons through a semi-automated algorithm that selected nearby pixels significantly correlated with each other.

We obtained a baseline F_0 by smoothing the calcium trace F in time and finding the minimum over a 20-s sliding window. We then computed $\Delta F/F$ by applying a causal exponentially weighted filter (τ = 0.2 s) to the fractional change $(F - F_0)/F$ (Jia et al., 2011).

To characterize psychometric performance in the 2AFC task, we calculated the proportions of trials with rightward choices (ignoring repeat trials that were sometimes introduced after errors) and fitted them with a psychometric function (e.g., Busse et al., 2011).

To measure task performance as a function of trial number, we considered easy trials (contrast $\geq$ 40%) and estimated the probability of a correct response as a function of trial as well as its confidence intervals (Smith et al., 2004). Daily performance was estimated by averaging across each day's easy trials.

In the 2AUC version of the task, the mouse was required to be still for 0.5–1 s after stimulus onset. This period of no movement was followed by an auditory go cue. Lack of movement within 1.5 s of the go cue was considered a no-go response, which was met with a reward for trials with zero contrast stimuli or with a 2-s white noise burst for all other stimuli. We trained mice in this 2AUC version by first training them in the 2AFC version (at least with high contrast) and then introducing zero contrast (no-go) trials.

To fit 2AUC data, we used the model in Equations 1, 2, and 3. We fit the 4 parameters of Equation 2 through multinomial logistic regression and optimized the 2 parameters in Equation 1. When measuring the effects of inactivation, we fitted the different inactivation conditions independently, while holding constant the parameters of Equation 1. This allowed us to capture the effects of inactivation with changes in the parameters of Equation 2.

Inactivation experiments were performed with mice expressing ChR2 in *Pvalb*-positive inhibitory neurons (B6;129P2-*Pvalb*$^{tm1(cre)Arbr}$/J crossed with Ai32). Mice were prepared with a clear skullcap similar to that used by Guo et al. (2014b) but with UV-curing optical adhesive. Inactivation light was produced by a 473-nm diode laser coupled to a fiber, producing ~1.5 mW in a spot of ~0.3 mm diameter, positioned over visual cortex (3.3–3.7 mm posterior, 2.1 mm lateral) or somatosensory cortex (0.8 mm posterior, 2.5 mm lateral). Inactivation was performed randomly in ~30% of trials. Light was delivered as a 40-Hz sinusoid beginning 33 ms before visual stimulus onset and lasting until the response. The task was 2AUC detection, but responses could be immediately made on stimulus onset.

For optogenetic dopamine stimulation, we used DAT-Cre mice (Jax 006660) backcrossed with C57BL/6J mice. We injected 1 μL of diluted virus (AAV5.EF1a.DIO.hChr2(H134R)-eYFP.WPRE) into VTA and SNc and implanted an optic fiber with tip 0.5 mm above the injection site. We waited 3 weeks for virus expression and then started behavioral training. On making a correct choice, animals received a short train of laser stimulation (473 nm, 12 pulses each lasting 10 ms and separated by 40 ms, power 10–15 mW measured at the fiber tip) and a simultaneous click sound.

To quantify ChR2 expression in dopamine neurons, 50-μm coronal sections were collected and immunostained with antibodies to EYFP and TH and secondary antibodies labeled with Alexa Fluor 488 and 594 (Tsai et al., 2009).

The contrast discrimination task is based on the 2AUC task, but gratings could be presented on both sides of the screen simultaneously, and mice were rewarded for choosing the grating with the highest contrast. Mice were first trained in the 2AUC detection task, and discriminations were introduced incrementally. Mice learned this discrimination task within a few days after learning the detection task.

SUPPLEMENTAL INFORMATION

Supplemental Information includes Supplemental Experimental Procedures, six figures, and one movie and can be found with this article online at http://dx.doi.org/10.1016/j.celrep.2017.08.047.

AUTHOR CONTRIBUTIONS

Conceptualization: C.P.B., A.L., N.A.S., P.Z.-H., J.F.L., K.D.H., and M.C. Methodology: C.P.B., A.L., N.A.S., P.Z.-H., S. Schröder, S. Soares, J.J.P., L.E.W., K.D.H., and M.C. Software: C.P.B., A.L., N.AS., and P.Z.-H. Formal Analysis: C.P.B., A.L., N.A.S., P.Z.-H., and M.J.W. Investigation: C.P.B., A.L., N.A.S., P.Z.-H., C.B.R., E.A.K.J., A.R., S. Schröder, and M.J.W. Writing – Original Draft: C.P.B., A.L., N.A.S., P.Z.-H., and M.C. Writing – Reviewing and Editing: C.P.B., A.L., N.A.S., P.Z.-H., E.A.K.J., J.J.P., S. Schröder, S. Soares, L.E.W., K.D.H., and M.C. Funding Acquisition: C.P.B., A.L., N.A.S., S. Schröder, E.A.K.J., K.D.H., and M.C. Supervision: K.D.H. and M.C. Project Administration: M.C.

ACKNOWLEDGMENTS

This work was supported by Senior Investigator Awards from the Wellcome Trust (095668 and 095669, to M.C. and K.D.H.), a Medical Research Council Doctoral Training Award (to C.P.B.), a Human Frontiers Science Program Fellowship (LT001071, to N.S.), a Sir Henry Wellcome Fellowship (106101, to A.L.), two Marie Skłodowska-Curie fellowships (656528 and 623872, to N.S. and to S. Schröder), a grant from the Simons Foundation (SCGB 325476) (to J.J.P.), Champalimaud Foundation internal support (to J.J.P.), a graduate fellowship from the Fundação para Ciência e Tecnologia (SFRH/BD/51895/2012, to S. Soares), and a Wellcome Trust PhD Studentship (to E.J.). M.C. holds the GlaxoSmithKline/Fight for Sight Chair in Visual Neuroscience.

Received: May 16, 2016
Revised: June 8, 2017
Accepted: August 14, 2017
Published: September 5, 2017

REFERENCES

Abrahamyan, A., Silva, L.L., Dakin, S.C., Carandini, M., and Gardner, J.L. (2016). Adaptable history biases in human perceptual decisions. Proc. Natl. Acad. Sci. USA *113*, E3548–E3557.

Albrecht, D.G., and Hamilton, D.B. (1982). Striate cortex of monkey and cat: contrast response function. J. Neurophysiol. *48*, 217–237.

Andermann, M.L., Kerlin, A.M., and Reid, R.C. (2010). Chronic cellular imaging of mouse visual cortex during operant behavior and passive viewing. Front. Cell. Neurosci. *4*, 3.

Bak, J.H., Choi, J.Y., Akrami, A., Witten, I.B., and Pillow, J. (2016). Adaptive optimal training of animal behavior. In Advances in Neural Information Processing Systems, D.D. Lee, M. Sugiyama, U.V. Luxburg, I. Guyon, and R. Garnett, eds. (MIT Press), pp. 1947–1955.

Beck, J.A., Lloyd, S., Hafezparast, M., Lennon-Pierce, M., Eppig, J.T., Festing, M.F., and Fisher, E.M. (2000). Genealogies of mouse inbred strains. Nat. Genet. *24*, 23–25.

Boynton, G.M., Demb, J.B., Glover, G.H., and Heeger, D.J. (1999). Neuronal basis of contrast discrimination. Vision Res. *39*, 257–269.

Burgess, C.P. (2016). Measuring correlates of perceptual decisions in mouse visual cortex. PhD thesis (University College London).

Busse, L., Ayaz, A., Dhruv, N.T., Katzner, S., Saleem, A.B., Schölvinck, M.L., Zaharia, A.D., and Carandini, M. (2011). The detection of visual contrast in the behaving mouse. J. Neurosci. *31*, 11351–11361.

Bussey, T.J., Holmes, A., Lyon, L., Mar, A.C., McAllister, K.A., Nithianantharajah, J., Oomen, C.A., and Saksida, L.M. (2012). New translational assays for preclinical modelling of cognition in schizophrenia: the touchscreen testing method for mice and rats. Neuropharmacology *62*, 1191–1203.

Cahill, H., and Nathans, J. (2008). The optokinetic reflex as a tool for quantitative analyses of nervous system function in mice: application to genetic and drug-induced variation. PLoS ONE *3*, e2055.

Carandini, M., and Churchland, A.K. (2013). Probing perceptual decisions in rodents. Nat. Neurosci. *16*, 824–831.

Ding, L., and Gold, J.I. (2013). The basal ganglia's contributions to perceptual decision making. Neuron *79*, 640–649.

García-Pérez, M.A., and Alcalá-Quintana, R. (2011). Improving the estimation of psychometric functions in 2AFC discrimination tasks. Front. Psychol. *2*, 96.

García-Pérez, M.A., and Alcalá-Quintana, R. (2013). Shifts of the psychometric function: distinguishing bias from perceptual effects. Q J Exp Psychol (Hove) *66*, 319–337.

Garrett, M.E., Nauhaus, I., Marshel, J.H., and Callaway, E.M. (2014). Topography and areal organization of mouse visual cortex. J. Neurosci. *34*, 12587–12600.

Glickfeld, L.L., Histed, M.H., and Maunsell, J.H. (2013). Mouse primary visual cortex is used to detect both orientation and contrast changes. J. Neurosci. *33*, 19416–19422.

Glickfeld, L.L., Reid, R.C., and Andermann, M.L. (2014). A mouse model of higher visual cortical function. Curr. Opin. Neurobiol. *24*, 28–33.

Goard, M.J., Pho, G.N., Woodson, J., and Sur, M. (2016). Distinct roles of visual, parietal, and frontal motor cortices in memory-guided sensorimotor decisions. eLife *5*, 5.

Guo, Z.V., Hires, S.A., Li, N., O'Connor, D.H., Komiyama, T., Ophir, E., Huber, D., Bonardi, C., Morandell, K., Gutnisky, D., et al. (2014a). Procedures for behavioral experiments in head-fixed mice. PLoS ONE *9*, e88678.

Guo, Z.V., Li, N., Huber, D., Ophir, E., Gutnisky, D., Ting, J.T., Feng, G., and Svoboda, K. (2014b). Flow of cortical activity underlying a tactile decision in mice. Neuron *81*, 179–194.

Hangya, B., Ranade, S.P., Lorenc, M., and Kepecs, A. (2015). Central cholinergic neurons are rapidly recruited by reinforcement feedback. Cell *162*, 1155–1168.

Harris, J.A., Hirokawa, K.E., Sorensen, S.A., Gu, H., Mills, M., Ng, L.L., Bohn, P., Mortrud, M., Ouellette, B., Kidney, J., et al. (2014). Anatomical characterization of Cre driver mice for neural circuit mapping and manipulation. Front. Neural Circuits *8*, 76.

Harvey, C.D., Coen, P., and Tank, D.W. (2012). Choice-specific sequences in parietal cortex during a virtual-navigation decision task. Nature *484*, 62–68.

Heintz, N., and Gerfen, C. The Gene Expression Nervous System Atlas (GENSAT) Project. http://www.gensat.org/index.html.

Herrnstein, R.J. (1964). Secondary reinforcement and rate of primary reinforcement. J. Exp. Anal. Behav. *7*, 27–36.

Huang, Z.J., and Zeng, H. (2013). Genetic approaches to neural circuits in the mouse. Annu. Rev. Neurosci. *36*, 183–215.

Huberman, A.D., and Niell, C.M. (2011). What can mice tell us about how vision works? Trends Neurosci. *34*, 464–473.

Jaramillo, S., and Zador, A.M. (2014). Mice and rats achieve similar levels of performance in an adaptive decision-making task. Front. Syst. Neurosci. *8*, 173.

Jia, H., Rochefort, N.L., Chen, X., and Konnerth, A. (2011). In vivo two-photon imaging of sensory-evoked dendritic calcium signals in cortical neurons. Nat. Protoc. *6*, 28–35.

Kiani, R., and Shadlen, M.N. (2009). Representation of confidence associated with a decision by neurons in the parietal cortex. Science *324*, 759–764.

Kim, K.M., Baratta, M.V., Yang, A., Lee, D., Boyden, E.S., and Fiorillo, C.D. (2012). Optogenetic mimicry of the transient activation of dopamine neurons by natural reward is sufficient for operant reinforcement. PLoS ONE *7*, e33612.

Lak, A., Costa, G.M., Romberg, E., Koulakov, A.A., Mainen, Z.F., and Kepecs, A. (2014). Orbitofrontal cortex is required for optimal waiting based on decision confidence. Neuron *84*, 190–201.

Lee, S.H., Kwan, A.C., Zhang, S., Phoumthipphavong, V., Flannery, J.G., Masmanidis, S.C., Taniguchi, H., Huang, Z.J., Zhang, F., Boyden, E.S., et al. (2012). Activation of specific interneurons improves V1 feature selectivity and visual perception. Nature *488*, 379–383.

Legge, G.E., and Foley, J.M. (1980). Contrast masking in human vision. J. Opt. Soc. Am. *70*, 1458–1471.

Lein, E.S., Hawrylycz, M.J., Ao, N., Ayres, M., Bensinger, A., Bernard, A., Boe, A.F., Boguski, M.S., Brockway, K.S., Byrnes, E.J., et al. (2007). Genome-wide atlas of gene expression in the adult mouse brain. Nature *445*, 168–176.

Licata, A.M., Kaufman, M.T., Raposo, D., Ryan, M.B., Sheppard, J.P., and Churchland, A.K. (2017). Posterior parietal cortex guides visual decisions in rats. J. Neurosci. *37*, 4954–4966.

Lien, A.D., and Scanziani, M. (2013). Tuned thalamic excitation is amplified by visual cortical circuits. Nat Neurosci *16*, 1315–1323.

Liu, D., Gu, X., Zhu, J., Zhang, X., Han, Z., Yan, W., Cheng, Q., Hao, J., Fan, H., Hou, R., et al. (2014). Medial prefrontal activity during delay period contributes to learning of a working memory task. Science *346*, 458–463.

Long, M., Jiang, W., Liu, D., and Yao, H. (2015). Contrast-dependent orientation discrimination in the mouse. Sci. Rep. *5*, 15830.

Madisen, L., Mao, T., Koch, H., Zhuo, J.M., Berenyi, A., Fujisawa, S., Hsu, Y.W., Garcia, A.J., 3rd, Gu, X., Zanella, S., et al. (2012). A toolbox of Cre-dependent optogenetic transgenic mice for light-induced activation and silencing. Nat. Neurosci. *15*, 793–802.

Madisen, L., Garner, A.R., Shimaoka, D., Chuong, A.S., Klapoetke, N.C., Li, L., van der Bourg, A., Niino, Y., Egolf, L., Monetti, C., et al. (2015). Transgenic mice for intersectional targeting of neural sensors and effectors with high specificity and performance. Neuron *85*, 942–958.

Nachmias, J., and Sansbury, R.V. (1974). Letter: Grating contrast: discrimination may be better than detection. Vision Res. *14*, 1039–1042.

Niell, C.M., and Stryker, M.P. (2010). Modulation of visual responses by behavioral state in mouse visual cortex. Neuron *65*, 472–479.

Nienborg, H., and Macke, J.H. (2014). Using Sequential Dependencies in Neural Activity and Behavior to Dissect Choice Related Activity in V2 (Society for Neuroscience).

Nithianantharajah, J., McKechanie, A.G., Stewart, T.J., Johnstone, M., Blackwood, D.H., St Clair, D., Grant, S.G., Bussey, T.J., and Saksida, L.M. (2015). Bridging the translational divide: identical cognitive touchscreen testing in mice and humans carrying mutations in a disease-relevant homologous gene. Sci. Rep. *5*, 14613.

Oh, S.W., Harris, J.A., Ng, L., Winslow, B., Cain, N., Mihalas, S., Wang, Q., Lau, C., Kuan, L., Henry, A.M., et al. (2014). A mesoscale connectome of the mouse brain. Nature *508*, 207–214.

Olds, J., and Milner, P. (1954). Positive reinforcement produced by electrical stimulation of septal area and other regions of rat brain. J. Comp. Physiol. Psychol. *47*, 419–427.

Pinto, L., and Dan, Y. (2015). Cell-type-specific activity in prefrontal cortex during goal-directed behavior. Neuron *87*, 437–450.

Poort, J., Khan, A.G., Pachitariu, M., Nemri, A., Orsolic, I., Krupic, J., Bauza, M., Sahani, M., Keller, G.B., Mrsic-Flogel, T.D., and Hofer, S.B. (2015). Learning enhances sensory and multiple non-sensory representations in primary visual cortex. Neuron *86*, 1478–1490.

Prusky, G.T., West, P.W., and Douglas, R.M. (2000). Behavioral assessment of visual acuity in mice and rats. Vision Res. *40*, 2201–2209.

Pryor, K. (1999). Don't Shoot the Dog! The New Art of Teaching and Training (Bantam).

Resulaj, A., and Rinberg, D. (2015). Novel behavioral paradigm reveals lower temporal limits on mouse olfactory decisions. J. Neurosci. *35*, 11667–11673.

Resulaj, A., Kiani, R., Wolpert, D.M., and Shadlen, M.N. (2009). Changes of mind in decision-making. Nature *461*, 263–266.

Sanders, J.I., and Kepecs, A. (2012). Choice ball: a response interface for two-choice psychometric discrimination in head-fixed mice. J. Neurophysiol. *108*, 3416–3423.

Schultz, W., Dayan, P., and Montague, P.R. (1997). A neural substrate of prediction and reward. Science *275*, 1593–1599.

Sclar, G., Maunsell, J.H., and Lennie, P. (1990). Coding of image contrast in central visual pathways of the macaque monkey. Vision Res. *30*, 1–10.

Smith, A.C., Frank, L.M., Wirth, S., Yanike, M., Hu, D., Kubota, Y., Graybiel, A.M., Suzuki, W.A., and Brown, E.N. (2004). Dynamic analysis of learning in behavioral experiments. J. Neurosci. *24*, 447–461.

Sridharan, D., Steinmetz, N.A., Moore, T., and Knudsen, E.I. (2014). Distinguishing bias from sensitivity effects in multialternative detection tasks. J. Vis. *14*, 14.

Tsai, H.C., Zhang, F., Adamantidis, A., Stuber, G.D., Bonci, A., de Lecea, L., and Deisseroth, K. (2009). Phasic firing in dopaminergic neurons is sufficient for behavioral conditioning. Science *324*, 1080–1084.

Wang, Q., and Burkhalter, A. (2007). Area map of mouse visual cortex. J. Comp. Neurol. *502*, 339–357.

Wang, Q., Gao, E., and Burkhalter, A. (2011). Gateways of ventral and dorsal streams in mouse visual cortex. J. Neurosci. *31*, 1905–1918.

Wekselblatt, J.B., Flister, E.D., Piscopo, D.M., and Niell, C.M. (2016). Large-scale imaging of cortical dynamics during sensory perception and behavior. J. Neurophysiol. *115*, 2852–2866.

Zingg, B., Hintiryan, H., Gou, L., Song, M.Y., Bay, M., Bienkowski, M.S., Foster, N.N., Yamashita, S., Bowman, I., Toga, A.W., and Dong, H.W. (2014). Neural networks of the mouse neocortex. Cell *156*, 1096–1111.

Cell Reports, Volume *20*

Supplemental Information

High-Yield Methods for Accurate Two-Alternative Visual Psychophysics in Head-Fixed Mice

Christopher P. Burgess, Armin Lak, Nicholas A. Steinmetz, Peter Zatka-Haas, Charu Bai Reddy, Elina A.K. Jacobs, Jennifer F. Linden, Joseph J. Paton, Adam Ranson, Sylvia Schröder, Sofia Soares, Miles J. Wells, Lauren E. Wool, Kenneth D. Harris, and Matteo Carandini

Supplementary Figures

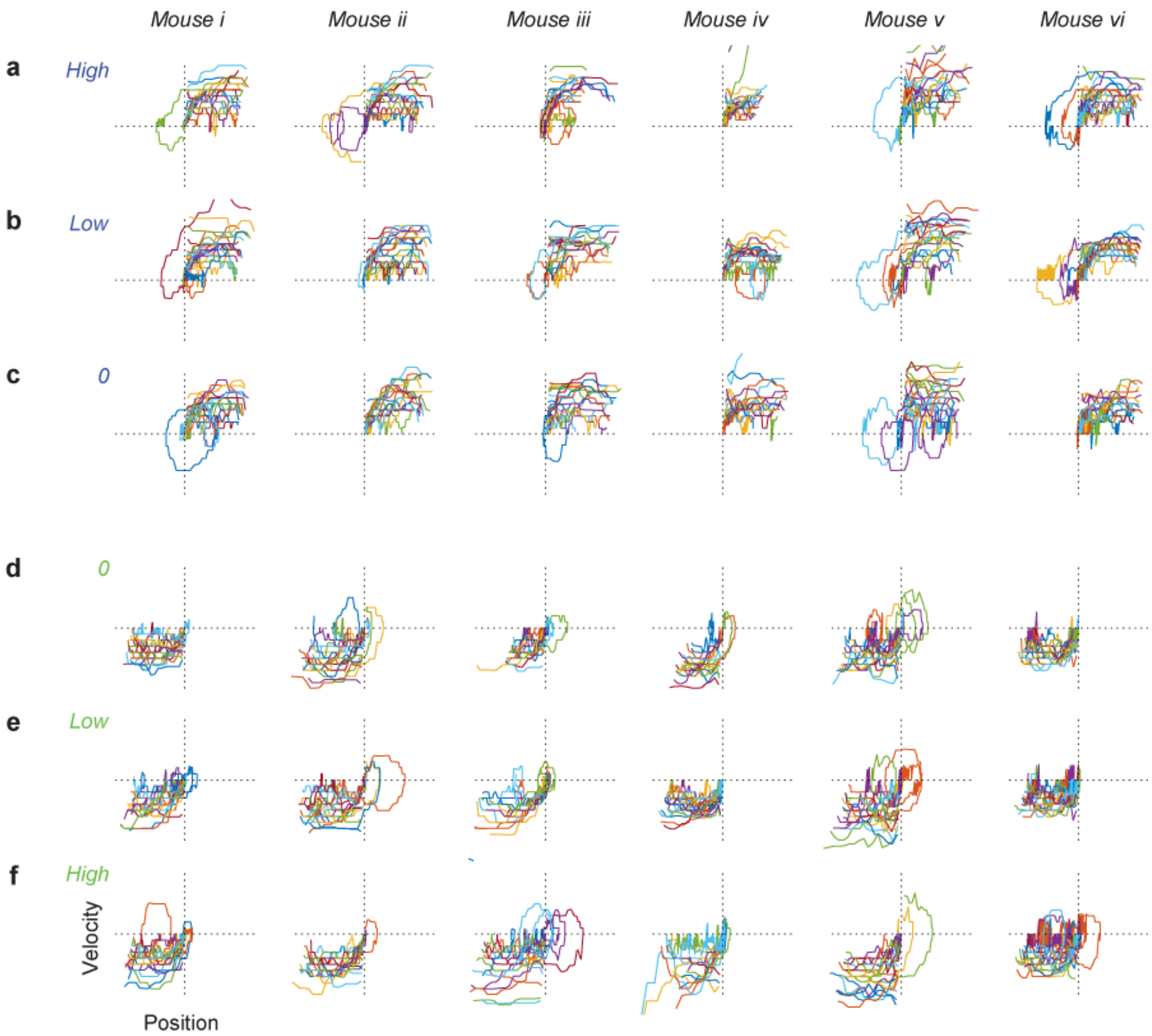

Supplementary Figure 1. Trajectories of wheel turns made by mice in response to stimuli. Related to Figure 1. Traces show evolution of position and velocity during trajectories for turns made between stimulus onset and attainment of choice threshold.

a-c: Trajectories that ended with a choice to the left, for stimuli that had high contrast on the left (a), low contrast on the left (b), or zero contrast (c). Any trials where the initial choice direction is inconsistent with the final choice must cross from one quadrant to the other (lower-left to upper-right), which is uncommon.

d-f: Same as **a-c**, for trajectories that ended with a choice to the right.

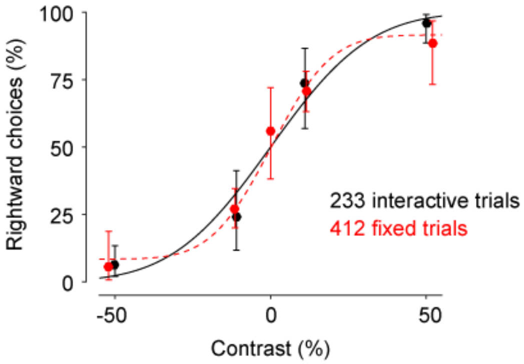

Supplementary Figure 2. Comparison of psychophysical performance in interactive trials vs. fixed-stimulus trials. Related to Figure 1.These data were obtained in a single session in which two types of trial were randomly interleaved. In normal interactive trials, the steering wheel moved the stimulus (black). In the remaining trials, the mouse completes choices by turning the wheel as normal, but the stimulus remains fixed at the onset position (red). The ordinate plots the percentage of times the mouse chose the stimulus on the right (R), as a function of stimulus contrast (positive for R stimuli, negative for L stimuli). The psychometric curves fit across the two sets of trials (curves) are similar.

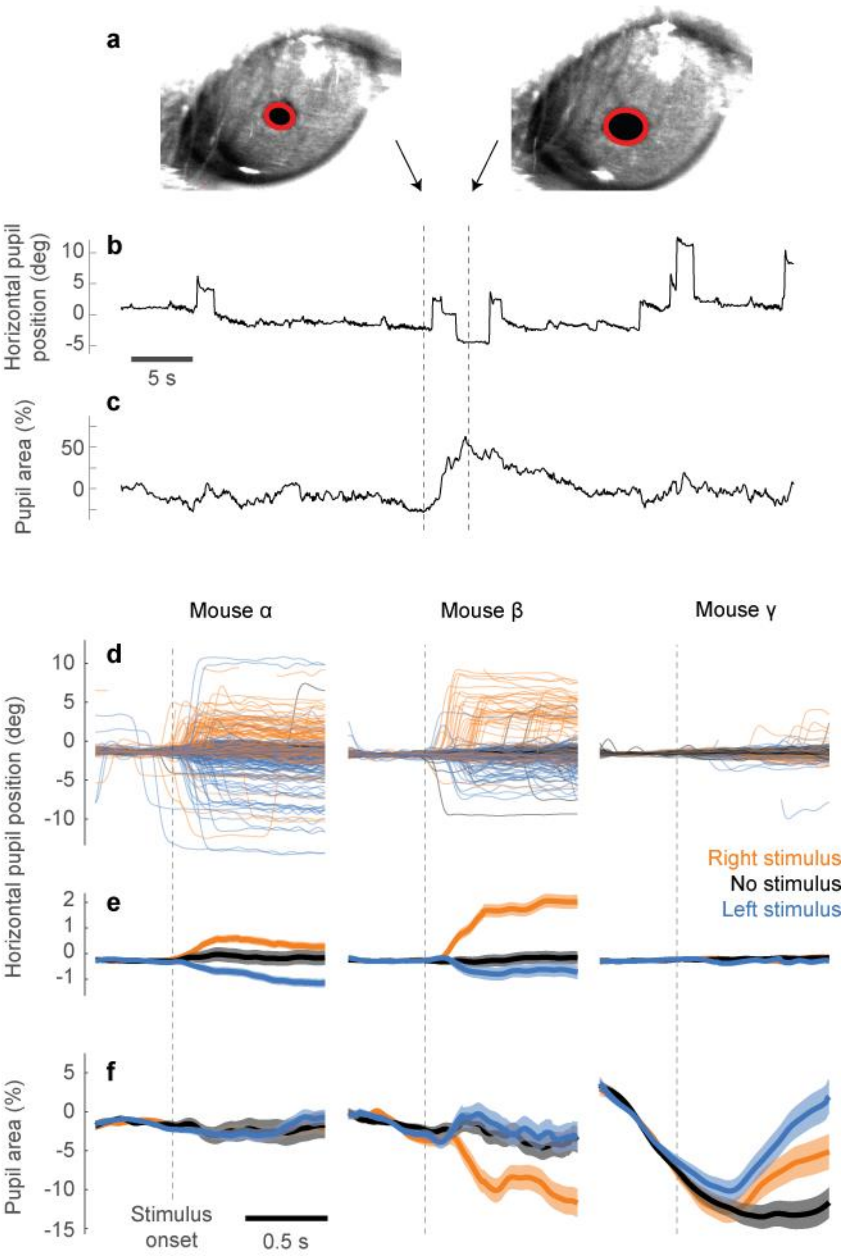

Supplementary Figure 3. Eye movements during task performance. Related to Figure 1.

a: Two example frames showing ellipses fit to the pupil.

b: Example traces of horizontal position calculated from movies of the eye. Saccades as small as ~2 deg are clearly visible. Dashed lines indicate the times of the two frames in **a**.

c: Same as **b**, for the pupil area (proportion change relative to the mean).

d: Traces of pupil position for each trial from three example mice. Traces are aligned to stimulus onset and colored according to stimulus condition: stimulus on left (blue), right (orange), or no stimulus (black).

e: Average of the traces in **d**. Notice different y-scale. Shaded area represents two s.e.m.

f: Same as **e**, for the pupil area.

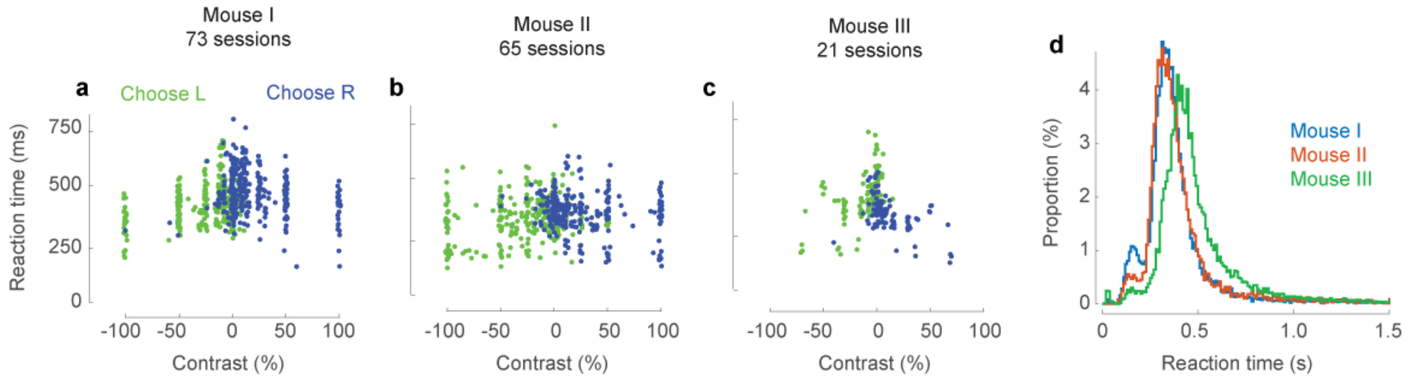

Supplementary Figure 4 Reaction times in the 2AUC task. Related to Figure 3.

a-c. Median reaction time for the example mice in Figure 3. Each dot indicates the median reaction time measured for stimuli of a given contrast in a single session, grouped by decisions made on the left (*green*) or on the right (*blue*).

d: Distribution of reaction times for all trials in the three mice. The standard deviations of the reaction time distribution were 206, 181, and 198 ms respectively. The proportions of trials with reaction times longer than 1 s were 3.6%, 2.1%, and 2.8% respectively. The vast majority of reaction times, therefore, are much shorter than the 1,500 ms that would result in a No-go response

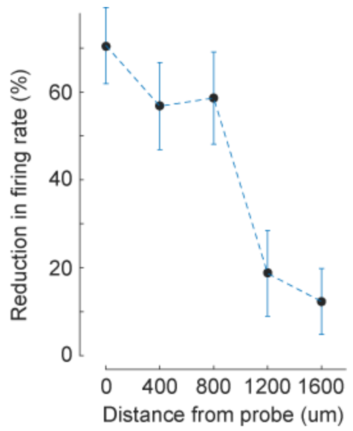

Supplementary Figure 5. Control electrophysiological measurements show optogenetic inactivation of visual cortex was spatially focused, with a radius of ~1 mm. Related to Figure 4. We inserted custom multisite electrodes in visual cortex, and pooled responses from n = 110 single-unit and multiunit clusters with broad waveforms. We moved the laser at different distances from the electrode (abscissa) and measured the reduction in firing rate relative to control firing rate (modulation index, ordinate). The spot size and laser power (1.5 mW) were the same as in the behavioral experiments.

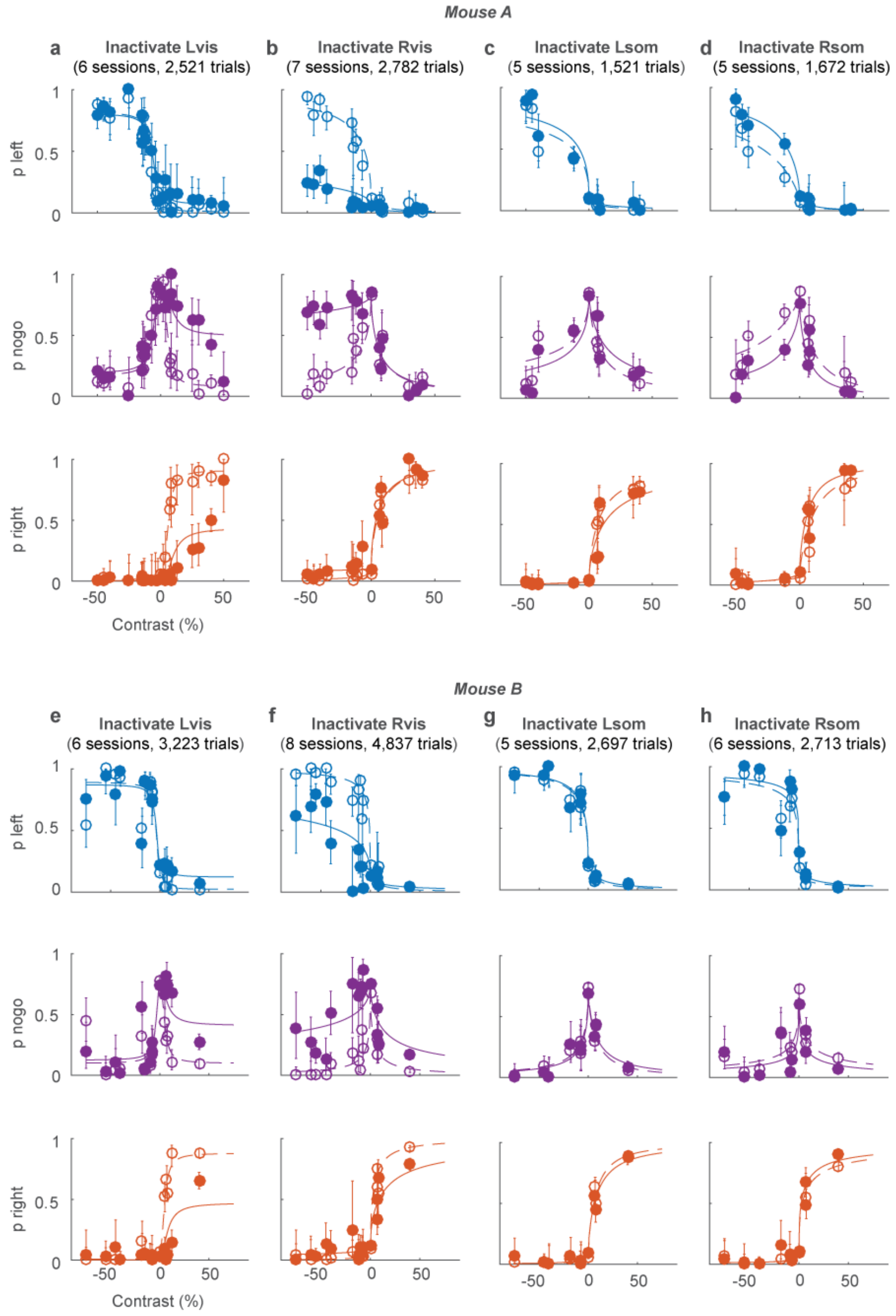

Supplementary Figure 6. Results of inactivation in 4 regions in two mice. Related to Figure 4.

a,**b**: Effects of inactivation of left and right visual cortex in mouse A (same data as Figure 4**b**,**c**).

c,**d**: Effects of inactivation of left and right somatosensory cortex in mouse A.

e-**h**: Same as **a**-**d**, for mouse B.

Supplemental Experimental Procedures

All experiments were conducted according to the UK Animals Scientific Procedures Act (1986). Male and female mice between the ages of 8-24 weeks were used for all experiments. Mice were C57BL/6J or transgenics with a C57BL/6J background. Measurements were made during the day (9 am to 8 pm). The daylight cycle for the mice was normal (8 am - 8 pm) except for some mice in the basic task (Figure 1), where it was inverted (9 pm – 9 am). We did not investigate the effect of daylight cycle on performance. Mice were housed on their own or in pairs.

Head-plate implant

Mice were implanted with metal plate on the cranium to enable their heads to be fixed. To perform this surgery, mice were injected with an anti-inflammatory drug (4 mg/kg Carprofen, subcutaneously) and anaesthetized using isoflurane (1–2%). Body temperature was maintained at 37°C using a heating pad and the eyes were protected with artificial tears to prevent drying (Viscotears). The head - plate was implanted chronically by fixing it to the cranium with dental cement (Sun Medical). After surgery, mice were allowed at least 4 days to recover before water control and behavioral training began.

Apparatus

The response wheel was a Lego part with a rubber tire (a cylinder 19 mm wide and 31 mm in diameter). Its angle was measured using a rotary encoder (typically, a Kübler 05.2400.1122.0100, with resolution 0.9° or about 0.5 mm of wheel circumference) whose signal was acquired using a data acquisition device (National Instruments USB-6212). Water was dispensed by opening a solenoid valve (Neptune Research 161T011) for a calibrated duration of time.

Stimuli were presented on an LCD monitor (refresh rate 60 Hz) placed in front of the animal. Monitor intensity values for each color channel were linearized by using measurements from a photodiode. This procedure, however, was generally carried out in experimental rigs but not in training rigs. Moreover, LCD panels are difficult to linearize because intensity varies strongly with viewing angle: if the line of sight is orthogonal to the screen at the center of the screen, the sides of the screen, and especially the corners, are substantially darker. Only towards the end of this project we realized how to overcome this difficulty: by placing plastic Fresnel lenses in front of the screens.

The initial apparatus used for these experiments involved multiple parts custom-built by a machine shop. Later versions rely entirely on off-the-shelf components and 3D-printed parts. The design for the latest version, together with a detailed parts list, is described at www.ucl.ac.uk/cortexlab/tools/wheel.

The task was managed by custom MATLAB software, through an open-source package called Signals (github.com/dendritic/signals). It uses a dataflow-style paradigm to allow concise and intuitive specification of stimulus presentation, task structure, and control of data acquisition. To control graphics presentation for visual stimulation, we used the Psychophysics Toolbox (Brainard, 1997; Pelli, 1997).

Stimuli were typically Gabor patches, i.e. sinusoidal gratings (typically, vertical, with wavelength 10°) in a Gaussian window. The Gaussian typically had standard deviation of ~10°. However, there was great variation across mice in these and other parameters, including position. In different mice we generally used different visual stimuli (different spatial frequency, size, temporal frequency, position, etc.) and all these factors contribute to the visibility of the stimuli. A future study could vary these attributes in a controlled manner and use our techniques to measure properties of mouse vision.

Training procedure

Before training, mice were acclimatized daily with being handled and with being head-fixed in the steering wheel rig, with its forepaws resting upon the wheel. The mouse was able to turn the wheel with left or right movements of its forepaws. It was able to consume droplets of water dispensed via a spout close to its mouth. This acclimatization phase lasted for 3 days, with the duration of restraint gradually increasing from 10-30 min in the first day to up to 3 hours in the third day.

Mice were then trained in the task typically in daily one -hour sessions over a period of weeks. During the first few sessions mice were trained on a simplified version of the task, with 100% or 50% contrast, no inter-trial delays, quiescent period, or open loop period. Once they began to start turning the wheel in both directions, the delays were increased to their final values. Once performance was

above chance level, lower contrasts were gradually introduced. Typically, mice were running on the final task parameters by week 2-3.

The default distance to move the wheel at the start of training was ~2 cm, or about a 45° turn. This was adjusted during training when it appeared that a mouse made a consistent movement in the correct direction but the movement was too short. This could happen, for instance, if the mouse's position relative to the wheel was inadvertently set differently one day than on previous days.

In this study, however, we used no quantitative criteria for advancement from one stage to the next. Different experimenters used different methods based on personal intuition and experience. For instance, some experimenters found it useful to move the wheel by hand in occasional trials on the first day of training, in case the mouse was making no effort to turn it on its own. Moreover, to break possible patterns of stereotyped responses, many experimenters introduced "correction trials": if an animal failed to give the correct response to a high-contrast stimulus, the stimulus was presented in the same location in all subsequent trials until the animal gave the correct answer. The responses given during correction trials were not used to calculate psychometric curves or fit the probabilistic model. Some experimenters also found it useful to provide stimuli more frequently on one side than on the other, to correct for side biases (i.e. provide more stimuli on the side where the mouse performs worse). In a future study, it would be useful to develop an automated training schedule, perhaps using the quantitative measures of performance shown in Figure 1 (which were made post-hoc, not during training), and perhaps tailoring the stimuli to defeat superstitious strategies that weigh past decisions and outcomes (Abrahamyan et al., 2016; Bak et al., 2016; Busse et al., 2011; Licata et al., 2017).

Most mice were trained using water as a reward. They were placed on a water control schedule in which they received a minimum daily amount of 40 ml/kg/day (1 ml/day for a typical 25 g mouse). For this purpose, however, it would not be appropriate to use the mouse's actual weight, because animals on water control tend to lose weight. Similarly, it would not be appropriate to use the weight on the first day of training, because animals grow with age. We thus estimated the weight that the animal would have had if it had not been on water control. To do this, we weighted the mouse on the first day of the water control schedule and referenced this weight to a standard curve *W(sex,age)* relating sex and age to body weight in animals that are not on water control (www.jax.org/jax-mice-and-services/strain-data-sheet-pages/body-weight-chart-000664). This procedure established that the mouse's weight was a fraction x of the mean of other mice of same sex and age (with $x<1$ or $x>1$ for mice lighter or heavier than average). From then on, the mouse's age- and animal-adjusted weight was taken to be $w = x\ W(sex,age)$. The minimum required water was estimated based on w.

The mouse was then weighted again on each training/testing day (typically 5-7 days/week), and signs of dehydration were monitored: skin tension, sunken eyes, and marked variations in general behavior (no mice showed any of these signs). The animal spent at most 3 hours/day in training/testing, typically in a single session/day (occasionally, two sessions/day). At the end of each session, the amount of water received was logged by software and controlled visually by the experimenter. At the end of the day, the animal received top-up fluids (in the form of appropriately weighted Hydrogel packages, to prevent accidental spilling and to minimize the perceived equivalence to the fluids received during the task) to ensure that it received the minimum daily amount. On days in which no training/testing took place, the mouse received the entire minimum daily amount in the form of Hydrogel. If the mouse weight dropped below 80% of the age- and animal-adjusted weight w, the minimum daily amount was increased, and if the weight dropped below 70% of w (a very rare event), the animal was given ad-lib water until weight recovered. Similarly, if signs of dehydration had ever been positive, the mouse would have been placed on ad-lib water until recovered.

Task reward was also calibrated throughout the training process. When mice were naïve and did few trials they would be given more per correct trial (~3 µL), and as they became proficient and were completing >300 trials they would typically be given ~2 µL.

Eye tracking

On many sessions (typically imaging, inactivation, and some training sessions) we recorded eye position. We used a camera (DMK 21BU04.H or DMK 23U618, The Imaging Source) with a zoom lens (ThorLabs MVL7000) focused on one of the eyes. When fully zoomed and placed ~20 cm from the mouse, this setup provided ~73 pixels/mm. To avoid contamination of the image by reflected monitor light relating to visual stimuli, the eye was illuminated with a focused infrared LED (SLS-0208A, Mightex; driven with LEDD1B, ThorLabs) and an infrared filter was used on the camera (FEL0750, ThorLabs; with adapters SM2A53, SM2A6, and SM1L03, ThorLabs). We acquired videos with MATLAB's Image Acquisition Toolbox (MathWorks).

For each video frame, we determined pupil size and location with the following steps: 1) Smooth the image with a 2D Gaussian filter of manually-selected width; 2) Manually select an intensity threshold that discriminates between pixels inside vs. outside the pupil; 3) Find the contour corresponding to this intensity value; 4) Fit a 2D ellipse to this contour by minimizing the mean squared error of:

$$Ax_i^2 + Bx_iy_i + Cy_i^2 + Dx_i + Ey_i = 1$$

where $(x_{i,}y_i)$ are coordinates of points on the contour. Pupil area and center position were calculated directly from this fit ellipse. Frames for which no contour could be detected or for which the fit ellipse was outside the range of possible values (typically due to blinks or grooming) were assigned NaN values. Relative pupil area A was then quantified as a proportion change relative to mean: $A = (a - \hat{a})/\hat{a},$ where a is the absolute area (in pixels) and $\hat{a}$ is the mean area across all frames. Position values were converted to deg of visual angle α by first converting from pixels to mm, then assuming that the pixel center moved on the surface of a sphere: $\alpha = 360m/\pi d$. Here, m m is the position in mm and d is the diameter of the eye. We did not measure this diameter but rather assumed it to be the customary $d = 3.4$ mm (Remtulla and Hallett, 1985).

Imaging V1 responses

The imaging experiments were performed in three 10-12 week old C57BL/6J female mice. During the initial surgery, in addition to implanting the head -plate we performed a 1 mm^2 craniotomy in the middle of a circular aperture in the head -plate. The craniotomy was centered in the right primary visual cortex. We then injected them with a GCaMP6m virus under the human synapsin promoter (AAV2/1-*syn*-GCaMP6m-WPRE, 50 nL undiluted 2x10^{13} genome copy/ml) from Penn Vector Core (Chen et al., 2013) into the center of the craniotomy (stereotaxic coordinates 2.8 mm lateral and 3.3 mm caudal to Bregma) at a depth of 250 µm beneath the dura. We then covered the craniotomy with a two-layer glass coverslip construction, and sealed it with dental cement. The mice were allowed to recover for 1 week before water control and head-fixed training began.

We began calcium imaging 3 weeks after virus injection. Imaging was performed using a Sutter two-photon movable objective microscope controlled by ScanImage (Pologruto et al. 2003). A Coherent Chameleon laser running at 1000 nm provided excitation, with power level controlled by a Conoptics Pockels cell. Images were acquired continuously at 12 Hz with a resolution of 128×128 pixels. An Olympus 20X objective was used for focusing. Imaging data was synchronized with behavioral and stimulus events by simultaneously acquiring signals with imaging frame events and screen refresh events. The latter were measured using a photodiode directly measuring the screen.

In each mouse, we chose a field of view with good GCaMP expression and mapped the preferred stimulus position of the field of view by repeatedly presenting a grating stimulus on a gray screen for 1 s with 1-2 s inter-stimulus intervals. Stimuli were presented at random positions in a 5×5 grid in the left hemifield. The mean stimulus response across the field of view was calculated at each stimulus position. The position evoking the largest response was taken as the field of view's position preference. Before behavioral imaging commenced, we shifted the position of the task stimulus to the preferred position of the chosen field of view (the shift was typically < 10°). Stimulus orientation and size were not optimized.

We first registered the raw calcium movies using an algorithm that aligns each frame to the peak cross-correlation with a reference frame using the discrete Fourier transform (Guizar-Sicairos et al., 2008). We found cell regions of interest (ROIs) by using a semi-automated algorithm that selected nearby pixels that are significantly correlated with each other. $\Delta F/F$ calcium signals of ROI traces were computed as in Jia et al. (2011). Briefly, from calcium traces F, we obtained a measure of baseline F_0 by smoothing F in time (0.75 s causal moving average) and finding the minimum over a (causal) sliding window (20 s). $\Delta F/F$ is computed by applying a causal exponentially weighted filter (τ = 0.2 s) to the fractional change $(F\text{-}F_0)/F$.

Measures of 2AFC performance

To characterize psychometric performance in the 2-alternative forced-choice task (2AFC, Figure 1**c**, Figure 2**a**,**e**, and Figure 5**e**) we fitted a classical psychometric functions of contrast. We calculated the proportion of trials with rightward choices (ignoring repeat trials that were sometimes introduced after errors), and we fitted them with a standard psychometric function (e.g. Busse et al., 2011):

$$\Psi(c) = \lambda + (1 - 2\lambda)Erf(\frac{c - \mu}{\sigma})$$

where c is signed stimulus contrast (positive values for stimuli on the right, negative for stimuli on the left), and Erf is the cumulative Gaussian function. The parameters μ and σ are the bias and slope of the psychometric function, and λ is the lapse rate, i.e. the fraction of trials that are guessed independently of contrast. In this formulation, we used similar lapse rates for left and right choices. In other cases it was preferable to allow two different biases. We performed the fitting via maximum likelihood estimation, using the MATLAB function *fminsearch* over the log likelihood function.

To measure task performance as a function of trial number (Figure 1**d**,**e**) we used the model of Smith et al. (2004). This model prescribes a state-space smoothing algorithm to characterize a learning curve (probability of a correct response as a function of trial) and its confidence intervals. We applied this analysis to easier (contrast ≥ 40%) trials. Daily performance was estimated by taking the mean performance across each day's trials. This procedure was performed after the experiments, to analyze performance, but could in principle also be integrated into an automated system that advances the mouse to subsequent training stages based on estimates of learning.

2AUC version

In the 2AUC version of the task we did not use an auditory cue at stimulus onset, and the mouse was required to be still for 0.5-1 s after stimulus onset. This period of no movement was followed by an auditory Go cue (12 kHz pure tone lasting 100 ms with a 10 ms onset and offset ramp (Figure 3**b**). If the animal did not respond within 1.5 s of the go cue, this was considered a No-go response. No go responses were rewarded for trials with zero contrast stimuli or were met with a 2 s white noise burst for all other stimuli.

Zero-contrast stimuli were presented in ~20% of the trials. A series of ~5 consecutive No-go responses drew the attention of the experimenter. If the animal had stopped turning the wheel even following high-contrast stimuli, this was taken to indicate that the session was finished.

Training mice in this 2AUC version was done by first training them in the 2AFC version (at least with high contrast), and then introducing zero-contrast (No-go) trials. This was done only after the mouse's reaction times were mostly <1 s (so that, if there really is a stimulus, the mouse responds in time). In 3 of 37 mice (8%), reaction times stayed too long so we did not attempt to train the 2AUC version. No-go trials were repeated when incorrect but the repeats are not included in further analyses.

Of 34 mice trained on the 2AUC task, five (15%) initially had difficulty with the No-go trials, and this difficulty suggested that they monitored only one side of the screen. Indeed, these mice chose the ignored side (instead of giving a No-go response) also when there were no stimuli. To overcome this difficulty, we typically increased the proportion of zero-contrast trials and of trials with stimulus on the ignored side, even to the point of entirely removing trials with stimuli in the monitored side. Once performance improved, we progressively rebalanced the stimulus presentation. This approach worked well in 4 of the 5 mice with this initial difficulty. In the fifth mouse, a major bias persisted even after 15 sessions, and training was abandoned.

In general we presented stimuli with probability 1/3 to appear on the left, 1/3 to appear on the right, and 1/3 to be zero contrast (requiring a no-Go response). In some cases, we wished to reduce the number of no-Go responses, so that the mice would incorrectly choose left or right when in fact the stimulus was absent. We achieved this by reducing the proportion of zero-contrast stimuli. This made the mouse less likely to give a no-Go response.

Fits of the probabilistic model

To fit 2AUC data (Figure 3, Figure 4, and Figure 6) we used the probabilistic model defined in Equations 1-3. We fit the 4 parameters of the decision variables (Equation 2) to the data obtained in individual sessions through multinomial logistic regression, and optimized the additional two parameters describing contrast sensitivity (Equation 1). The resulting model has 6 parameters. For the data in Figure 3, these values are listed in Table 1.

	b_L	b_R	s_L	s_R	c_{50}	n	Classified
Mouse I	-0.6	-0.6	7.1	6.3	6	1.6	79%
Mouse II	-1.6	-0.5	9.5	6.9	25	0.8	76%
Mouse III	-0.9	-1.1	3.4	5.0	4	1.6	75%

Table 1. Fit parameters and fit quality for the three data sets illustrated in Figure 3. The first six columns are the parameters of the model. The seventh column is the percentage of trials that was correctly classified by the model.

Cross-validation indicated that for those data sets there would be no loss in fit quality if one imposed $s_L = s_R = s$, thus removing one free parameter. In those fits the bias parameters b_L and b_R changed by <0.3 and the values for s for the 3 mice were 6.6, 8.4, and 4.0, intermediate between the values found for s_L and s_R.

The model was fit by maximum likelihood estimation, using either MATLAB's inbuilt *fmincon* function or the *GLMNET* package (Qian,et al. 2013). The parameters c_{50} and n in Equation 1 were constrained to the ranges 0.1-80%, and 0-3.

In a logistic model, there is no established method to quantify fit quality. The natural approach would be to compare alternative models, which is not our goal here. As an alternative, one can simply calculate how well the model classifies the choice of each trial by taking the model's "choice" in each trial to be the one for which it predicts the maximum probability. By this measure, the model did well, correctly predicting >75% of the choices (Table 1).

When measuring the effects of inactivation (Figure 4 and Supplementary Figure 6), we fitted the different inactivation conditions independently, while imposing that the parameters of Equation 1, c_{50} and n, were constant across conditions. This allowed us to capture the effects of inactivation with changes in the 4 parameters of Equation 2.

Cortical inactivation

Inactivation experiments were performed with transgenic mice expressing ChR2 in Pvalb-positive inhibitory interneurons, obtained by crossing a $Pvalb^{tm1(cre)Arbr}$ driver (Jax #008069) with an Ai32 reporter (Jax #012569). Mice were prepared with a clear skull cap similar to that of Guo et al. (2014b) but with UV-curing optical adhesive (Norland Optical Adhesives #81, Norland Products Inc., Cranbury, NJ; from ThorLabs) instead of clear dental acrylic, and metal head -plate for head-fixation. In brief, the implantation surgery proceeded as follows. The dorsal surface of the skull was cleared of skin and periosteum and prepared with a brief application of green activator (Super-Bond C&B, Sun Medical Co, Ltd, Japan). A thin layer of cyanoacrylate was applied to the skull and allowed to dry. Two to four thin layers of UV-curing optical glue were applied to the skull and cured (~10 s per layer) until the exposed skull was covered (thin layers were used to prevent excessive heat production). Super-Bond polymer was applied around the edges to join the skin and the clear skull cap and enhance stability. A head-plate was attached to the skull over the interparietal bone with Super-Bond polymer.

Light for inactivation was produced by a 473 nm diode laser (LuxX diode laser, Photon Lines Ltd) coupled to a fiber and collimated to a circle of approximately 0.3 mm diameter on the skull. Total laser power at the surface of the skull was ~1.5 mW. The laser was mounted on a manipulandum, which was manually positioned at stereotaxic coordinates for the cortical regions, defined relative to Bregma: 3.3-3.7 mm posterior, 2.1 mm lateral for visual cortex; 0.8 mm posterior, 2.5 mm lateral for somatosensory cortex. Light was delivered as a 40 Hz sinusoid beginning 33.2±5.5ms (mean ± standard deviation) before the visual stimulus onset and lasting until the mouse made a response. The task was the 2AUC detection variant, but responses could be made immediately upon stimulus onset. During individual sessions, inactivation was performed on approximately 30% of trials, randomly selected. One session out of 34 was excluded because performance on trials without laser inactivation was poor (max percent correct <50% for highest contrast stimuli on one side).

Optogenetic dopamine stimulation

For optogenetic dopamine stimulation we used DAT-Cre mice that were heterozygous for Cre recombinase under the control of DAT gene (B6.SJLSlc6a3tm1.1(cre)Bkmn/J, Jackson Laboratory) backcrossed with C57/BL6J mice. We injected 1 µL of diluted virus (AAV5.EF1a.DIO.hChr2(H134R)-eYFP.WPRE, 2.8x10^{12} unit/ml) into VTA and SNc (injection coordinates, from Bregma: AP = -3 mm, lateral: 0.5 mm and dorsal-ventral: 4.4 mm). An optic fiber was implanted over the same stereotaxic coordinate but with the fiber tips 0.5 mm above the virus injection site. The fiber and the head -plate were secured with dental cement. We waited 3 weeks for virus expression before starting behavioral training. These mice had free access to food and water in their home cages and were trained in the 2AFC version of the task. In each trial, upon making a correct choice, animals received a short train of laser stimulation (473 nm, 12 pulses, pulse duration: 10 ms, inter pulse interval: 40 ms, laser power: 10-15 mW, measured at the tip of the fiber that was implanted in the brain) and a simultaneous click sound.

To quantify the specificity of ChR2 expression in dopamine neurons, animals were anesthetized (with sodium Pentobarbital) and perfused with 1X PBS followed by 4% formaldehyde in PBS. The brains were post-fixed in the same solution overnight and then kept in PBS containing 30% sucrose until settling. 50 µm coronal sections were collected and washed in PBS. Localization of fiber optic, DA cell bodies as well as ChR2-EYFP was confirmed using immunohistochemical methods (Tsai et al., 2009). Sections were immunostained with antibodies to TH (New Market Scientific, catalog No. 22941) and EYFP (Abcam, catalog No. AB6556) and secondary antibodies labeled with Alexa Fluor 488 and 594, respectively (Life Tech, catalog Nos. A11034 and A11032). We quantified infection efficiency and specificity by counting cells (1,460 neurons) from 121 confocal images collected from 11 animals.

Contrast discrimination task

This task is based on the 2AUC task above, but gratings could be presented on both sides of the screen simultaneously, and the mice were rewarded for choosing (i.e. centering) the grating with the highest contrast, or rewarded 50% of the time if grating contrasts were equal. As in the 2AUC task, no response after 1.5 seconds was registered as a no-go response and rewarded only if no stimulus was present.

Discriminations are introduced incrementally starting with easy discriminations and ending with equal contrasts on both sides. Adding harder discriminations was done at the discretion of the experimenter, typically on the very next session; no quantitative criteria were used. All mice that we attempted to train on this version learned it within a few days, starting from the 2AUC detection task.

References

Brainard, D.H. (1997). The Psychophysics Toolbox. Spatial Vision 10, 433-436.;

Chen, T.W., Wardill, T.J., Sun, Y., Pulver, S.R., Renninger, S.L., Baohan, A., Schreiter, E.R., Kerr, R.A., Orger, M.B., Jayaraman, V., et al. (2013). Ultrasensitive fluorescent proteins for imaging neuronal activity. Nature 499, 295-300.

Guizar-Sicairos, M., Thurman, S.T., and Fienup, J.R. (2008). Efficient subpixel image registration algorithms. Opt Lett 33, 156-158.

Pelli, D.G. (1997). The VideoToolbox software for visual psychophysics: Transforming numbers into movies. Spatial Vision 10, 437-442.

Pologruto, T.A., Sabatini, B.L., and Svoboda, K. (2003). ScanImage: flexible software for operating laser scanning microscopes. Biomed Eng Online 2, 13.

Qian, J., Hastie, T., Friedman, J., Tibshirani, R., and Simon, N. (2013) Glmnet for Matlab.

Remtulla, S., and Hallett, P.E. (1985). A schematic eye for the mouse, and comparisons with the rat. Vision Res 25, 21-31.
